# Fluorinated Block Copolymer: An Important Sorbent Design Criteria for Effective PFOA Removal from Its Aqueous Solution

**DOI:** 10.1021/acsapm.4c03792

**Published:** 2025-02-03

**Authors:** Sadifa Anjum, Michael Arik, Arya Patel, Nyalah Abasali, Laying Wu, Amrita Sarkar

**Affiliations:** † Department of Chemistry & Biochemistry, Montclair State University, Montclair, New Jersey 07043, United States; ‡ College of Science and Mathematics, Montclair State University, Montclair, New Jersey 07043, United States; § Sokol Institute for Pharmaceutical Life Sciences, Montclair State University, Montclair, New Jersey 07043, United States

**Keywords:** linear triblock copolymer, fluoropolymer, PFOA removal, fluorophilic interactions, sorbent

## Abstract

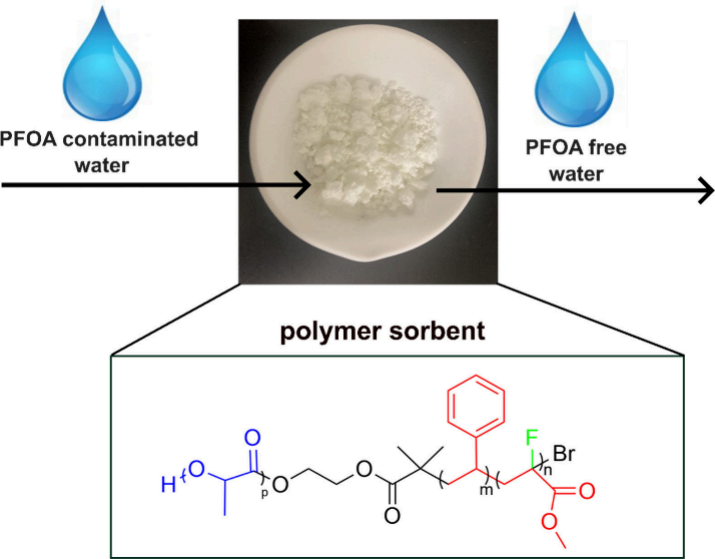

An important question remains unresolved, despite extensive studies on polymer-based sorbents for adsorbing poly and perfluoro alkyl substances (PFAS): How does the positioning of the fluorine-rich segment in polymer affect PFAS removal? Herein, we designed a linear, uncharged triblock copolymer incorporating a fluorinated moiety in the polymer backbone, which effectively removed perfluorooctanoic acid (PFOA) from water. In contrast, polymers without fluorogenic moiety or with it in the side chains showed significantly poorer PFOA removal. Our finding suggests that PFOA adsorption is more influenced by C–F**···**F–C interactions when the fluorinated segment is in the polymer backbone, not in the side chain.

Per- and polyfluoroalkyl substances (PFAS), present in a diverse array of industrial and household products, including cookware, coatings, aqueous firefighting foam, and adhesives, have emerged as a significant environmental concern.^1^ Their detection in natural water sources raises alarms regarding the potential threats to human health. PFAS persist in the environment due to the presence of chemically resistant hydrophobic carbon–fluorine (C–F) bonds, which are challenging to degrade.^1−3^ Consequently, developing efficient, environmentally friendly PFAS removal techniques has become an essential and evolving area of research.^4^ It has been reported that the combined contributions of fluorophilic interactions (an intermolecular dispersion force C–F**···**F–C)^4^ and electrostatic attraction within a sorbent are more effective for PFAS sorption than hydrophobic forces alone.^1,2,5−12^ For instance, Trabolsi and colleagues^10^ designed a fluorine-rich calix[4]-arene-based porous polymer utilizing host–guest chemistry, which demonstrated excellent adsorption of perfluorooctanoic acid (PFOA) via establishing a favorable C–F**···**F–C interactions between the fluorinated linkers and the polymer’s tail and PFOA. Likewise, a block copolymer incorporating perfluoropolyether (PFPE) and quaternized ammonium groups established both C–F**···**F–C and electrostatic interactions with PFOA, enabling effective removal of PFOA from aqueous solutions.^5^ Additionally, a fluorinated redox-active amine functionalized copolymer, developed by Su and colleagues,^2^ successfully achieved short-chain PFAS sorption through the combined effects of electrostatic and fluorophilic interactions. Separately, Choudhary et al.^13^ reported that PFOA adsorption was driven by electrostatic and hydrophobic interactions between ammonium ions and fluorinated linkers in β-cyclodextrin. Recent molecular dynamic (MD) simulations have indicated that the proximity of fluorinated atoms to cationic sites in a polycationic hydrogel creates an optimal environment for efficiently adsorbing short-chain PFAS, such as GenX (ammonium perfluoro-2-propoxypropionate), which has raised various health concerns.^11^ Collectively, these studies confirm that the interplay of hydrophobic, fluorophilic, and electrostatic interactions within a sorbent can significantly influence its sorption capacity, selectivity, and removal kinetics. Conversely, Whittaker and colleagues designed aqueous-soluble polymers incorporating a fluorinated moiety (PFPE) and a hydrophilic segment (poly(oligo(ethylene glycol)methyl ether acrylate), without including electrostatic interactions in their design.^6^ Despite this, these polymers exhibited a high affinity for PFOA, suggesting that high fluorine content in a polymer is critical for selective interaction with PFAS. Nevertheless, an important question regarding sorbent design remains unresolved: how does the position of the fluorine-rich segment within a polymer affect the binding and removal of PFOA? To facilitate understanding, we designed an uncharged linear triblock copolymer system that combines fluorophilic and hydrophobic interactions for PFAS binding in the presence of a short segment exhibiting hydrophilic forces. Four distinct polymers are designed: poly(hexyl acrylate-*block*-styrene-*block*-lactide) (**HSL)**, poly(hexyl acrylate-*block*-2,3,4,5,6-pentafluorostyrene-*block*-lactide) (**HFSL)**, poly(2,2,3,4,4,4-hexafluorobutyl methacrylate-*block*-styrene-*block*-lactide) (**HFBuMaSL)**, and poly(methyl-2-fluoroacrylate-*block*-styrene-*block*-lactide) (**MeFSL)**. Each polymer represents varying combinations of fluorinated and hydrophobic segments, incorporated in a polymeric backbone or in the side chains. Structures of the proposed sorbent candidates are presented in Scheme 1. A control nonfluorinated polymer **HSL** (**Candidate 1**) is designed with a backbone of hydrophobic hexyl acrylate (H) and styrene (S), while the mid and terminal blocks are highlighted in red. No F-rich moiety is incorporated in **HSL**, allowing for comparison with the other fluorinated candidates to assess the necessity of fluorination for PFAS removal. Conversely, fluorophilic segments including 2,3,4,5,6-pentafluorostyrene (FS) (middle block, shown in green) and 2,2,3,4,4,4-hexafluorobutyl methacrylate (HFBuMa) (terminal block on the right, also shown in green) are incorporated into the side chains of **Candidate 2 HFSL** and **Candidate 3 HFBuMaSL**, respectively, with alteration of the fluorophilic/hydrophobic block sequences. Lastly, **Candidate 4 MeFSL** is designed incorporating methyl-2-fluoroacrylate (MeF) in the polymer backbone, as illustrated in Scheme 1.

**Scheme 1:**
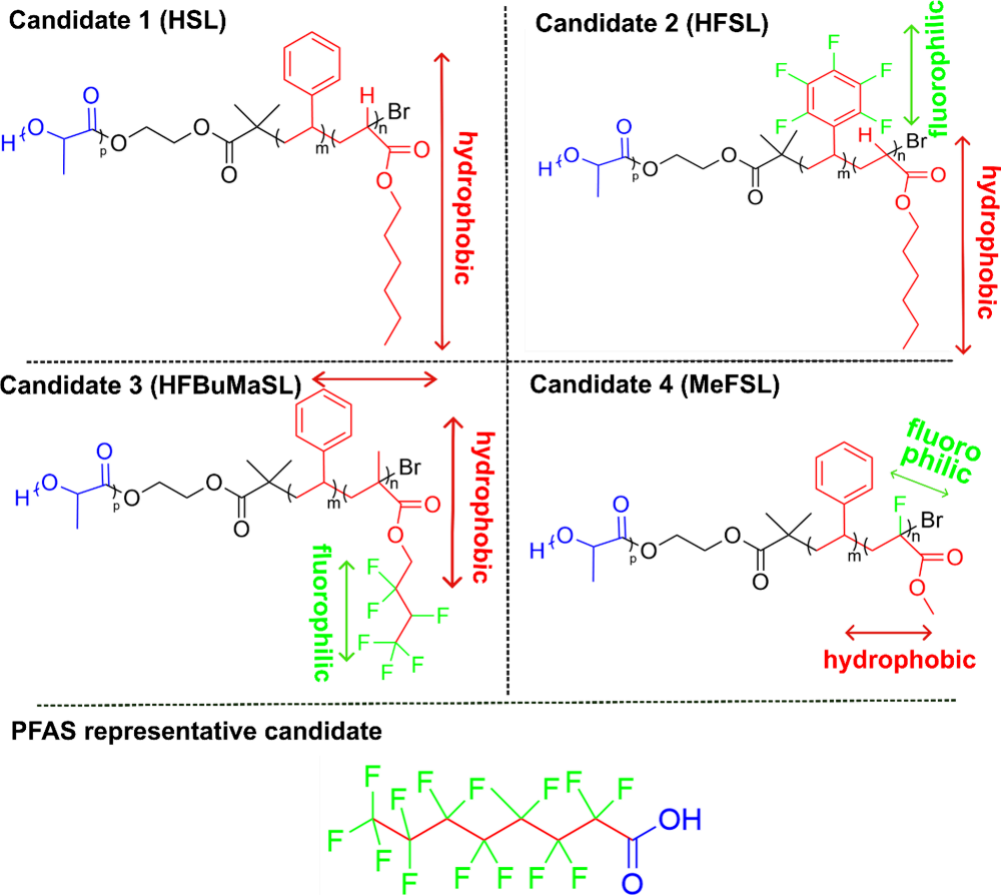
Chemical Structures of Polymer Sorbent Candidates and PFOA, a Model Representative Long-Tail PFAS Color code represents different interactions.

F-rich segments were introduced to **Candidates 2–4** to enhance the C–F**···**F–C interactions between the polymers and PFOA, a representative long-chain PFAS. A short segment of polylactide (terminal block on the left, depicted in blue), featuring a hydrophilic alcohol end group, was chosen for all four candidates, as it is relatively less hydrophobic compared to the described fluorinated moieties including FS, HFBuMa, and MeF. Nonfluorinated polylactide (lacking fluorophilic interactions) is anticipated to facilitate the sorption of PFOA by establishing hydrogen bonds between the carboxyl group of PFOA and the hydroxyl group or oxygen-rich lactide. However, please note that no traditional hydrophilic segment (e.g., poly(ethylene oxide)) was attached to this design to minimize the hydrogen bond formation and maximize the efficacy of hydrophobic and fluorophilic interactions for PFOA sequestration. Lastly, electrostatic interactions were excluded from the current study to elucidate the exclusive role of fluorophilic forces in the PFOA removal process, despite the acknowledged significance of electrostatic interactions in PFAS sorption.^1,2,11−14^ Proposed polymer candidates were synthesized via controlled radical and organolactide ring opening polymerizations (ROP).^15−18^ Synthesis of a representative **Candidate 2 HFSL** is outlined in Scheme 2.

**Scheme 2:**
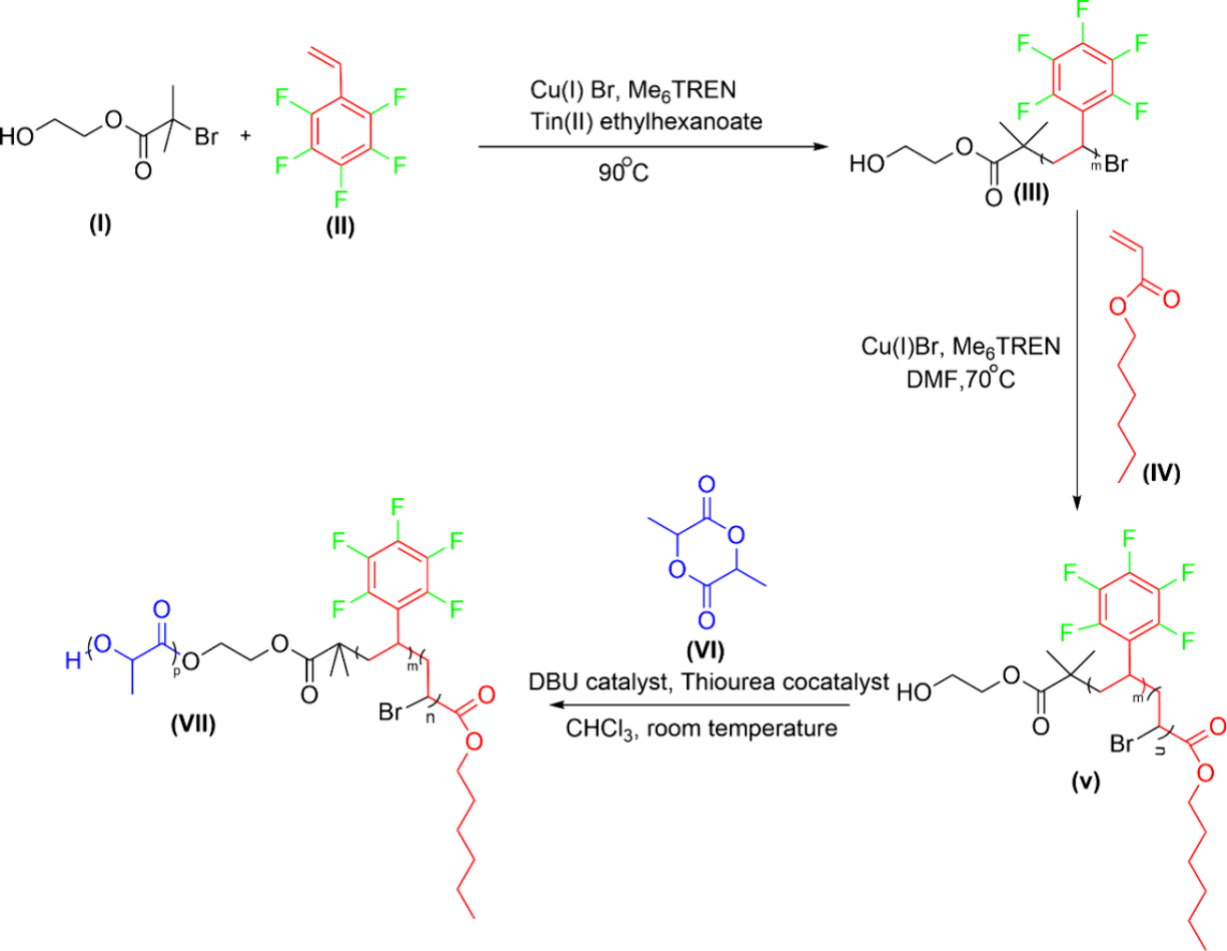
Schematic Illustration for the Synthesis of **Candidate 2 HFSL**

Briefly stated, we prepared macroinitiator poly(2,3,4,5,6-pentafluorostyrene) (FS) **(III)** via activators regenerated by electron transfer atom transfer radical polymerization (ARGET ATRP)^15^ using dual initiator 2-hydroxyethyl-2-bromoisobutyrate **(I)** and monomer 2,3,4,5,6-pentafluorostyrene **(II)**. This macroinitiator was extended to poly(hexyl acrylate-*block*-2,3,4,5,6-pentafluorostyrene) (HFS) **(V)** through the polymerization of hexyl acrylate via ATRP.^16−18^ This diblock was chain extended to triblock HFSL **(VII)** following organolactide ROP using monomer lactide **(VI)**, catalyst 1,8-diazabicyclo[5.4.0]undec-7-ene (DBU) and cocatalyst thiourea.^15^ Synthesized polymers were characterized by proton and fluorine nuclear magnetic resonance (^1^H NMR, ^19^F NMR), and size exclusion chromatography (SEC). Characterization data for a representative polymer **HFSL** is shown in Figure 1.

**Figure 1:**
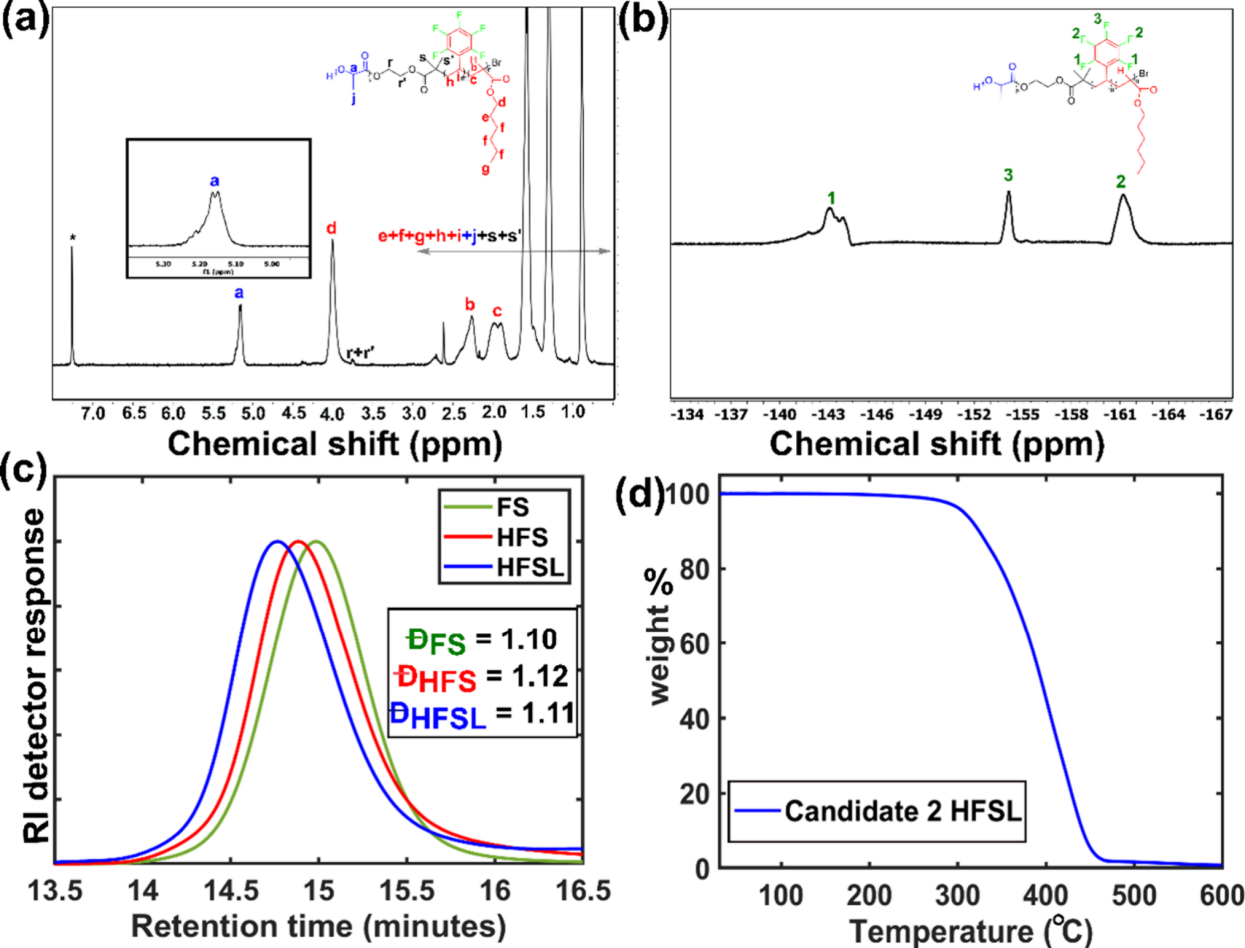
(a) ^1^H NMR, (b) ^19^F NMR, (c) SEC, and (d) TGA for a representative polymer **Candidate 2 HFSL**. NMR was obtained in CDCl_3_. A strong peak at 7.26 ppm (denoted by an asterisk, *) is due to the residual CHCl_3_ in the NMR solvent. SEC was performed in tetrahydrofuran (THF), calibrated with PS standards. For TGA analysis, the polymer sample was heated from 30 °C to 600 °C at a heating rate of 10 °C/min.

In the ^1^HNMR spectrum (Figure 1a), the chemical shifts (δ) at 1.80–2.05, and 2.26 ppm are assigned to the −CH_2_ and −C(H)Br protons in the polyhexyl acrylate (labeled as “c” and “b”, respectively). Whereas the signal for −CH_2_ protons (labeled as “d”) for polyhexyl acrylate was found at 4.01 ppm. A broad signal observed at 5.10–5.24 ppm is linked to the −CH protons (labeled as “a”) for polylactide. The sum of the aliphatic −CH–CH_2_ protons for pentafluorostyrene (labeled as “h” and “i”) was noted to be in the range of 1.70–3.0 ppm. Since all aromatic protons in pentafluorostyrene are substituted with F atoms, no signals were observed at ∼7 ppm. **HFSL** was further characterized by ^19^F NMR (Figure 1b); F atoms located at the *ortho* (1), *para* (3), and *meta* (2) positions of styrene contribute to the broad peaks at −144.50, −154.14, and −161.18 ppm, respectively. This confirms the successful incorporation of pentafluorostyrene into **HFSL**.^19^ The SEC profile (Figure 1c) is symmetric and shows a clear shift toward higher molecular weight (lower elution time). The narrow molar mass dispersity (*Đ*) suggests a well-controlled polymerization. Please note that the number-average molar masses (*M*_n_) estimated by SEC are not always accurate. We often noticed additional peaks for fluorinated polymers at the lower elution time (Figure S24, Figure S31). This is attributed to the formation of micelles or aggregate in the selective solvent (THF, eluent in our SEC system), which renders the SEC data unreliable.^20^ Moreover, the *M*_n,SEC_ value of fluorinated polymers differ significantly from that of *M*_n,NMR_, likely due to the difference in hydrodynamic volume between the fluorinated segments and the calibration agent polystyrene used in the SEC column.^21,22^ Detail synthesis and characterization of polymer candidates are described in Section S2 and Figures S1–S33 in the Supporting Information (SI). **HFSL** was further analyzed by thermogravimetry (TGA) in a nitrogen atmosphere, revealing its stability up to 300 °C (Figure 1d). It is noteworthy that all fluorinated polymers with similar molar masses exhibit greater thermal stability than the nonfluorinated polymer **HSL** (Figure S34). This enhanced stability is attributed to the presence of fluorine substitutions^23^ in the polymer backbone and side chains, as discussed in Section S3 in the SI. The differential scanning calorimetry (DSC) trace of **HFSL** revealed three distinct glass-transition temperatures (*T*_g_), at −56, 104.5, and 30 °C, confirming the existence of phase-separated, immiscible blocks comprising polyhexyl acrylate, polypentafluorostyrene, and polylactide, respectively (Figure S35). Polymer candidates with comparable molar masses, investigated for removal of PFOA from aqueous solutions, are presented in Table 1.

**Table 1:** Polymer Candidates Explored for the PFOA Removal Process^a^

sample ID	*M*_n,NMR_^b^ (kg/mol)	*M*_n,SEC_^c^ (kg/mol)	*Đ*^c^	block wt %^d^	*T*_d,50%_^e^ (°C)	*T*_g_^f^ (°C)
**Candidate 1: HSL7**	32	40	1.24	Wt%_PS_ = 39	328	*T*_g,PHA_ = −52.8
				Wt%_PHA_ = 50		*T*_g,PS_ = 108
				Wt%_PLA_= 11		*T*_g,PLA_ = 30.1
						
**Candidate 2:HFSL5**	29	49.3	1.28	Wt%_FS_ = 45	401	*T*_g,PHA_ = −56
				Wt_pPHA_= 32		*T*_g,FS_ = 104.5
				Wt%_PLA_ = 23		*T*_g,PLA_ = 30
						
**Candidate 3: HFBuMaSL5**	25	–g	–g	Wt%_PS_= 49	382	*T*_g,PS_ = 96.5
				Wt%_HFBuMa_ = 28		*T_g,PLA_**=** 33.5–49.5
				Wt%_PLA_= 23		*T_g,HFBuMa_ = 33.5–49.5
						
**Candidate 4: MeFSL2**	29	–g	–g	Wt%_PS_ = 43	393	*T_g,MeF_ = 80–135.7
				Wt%_MeF_ = 35		*T_g,PS_**=** 80–135.7
				Wt%_PLA_ = 22		*T*_g,PLA_**=** 25

a An asterisk prefix symbol (*) indicates that broad peaks were found. Note that the *T*_g_ values for PLA and HFBuMa in HFBuMaSL5 and MeF and PS in MeFSL2 closely coincide.

b Estimated by ^1^H NMR.

c Obtained by SEC performed in THF against PS standards.

d The weight fraction percentages (*W*_f_) for each block were calculated based on *M*_n,NMR_. For example, for HSL7, each block wt % was calculated using the terms *M*_n,NMR,PS_/(*M*_n,NMR,total_), (*M*_n,NMR,HS_ – *M*_n,NMR,PS_)/(*M*_n,NMR,total_), and (*M*_n,NMR,HSL_ – *M*_n,HS_)/(*M*_n,NMR,total_). Here, PS = polystyrene, PHA = polyhexyl acrylate, PLA = polylactide, FS = polypentafluorostyrene, HFBuMa = polyhexafluorobutyl methacrylate, and MeF = polyfluoromethacrylate.

e Thermal stability was determined by TGA.

f *T*_g_ measured by DSC.

g Not determined/confirmed via SEC measurements due to the formation of polymer micelles in SEC solvent THF.

Apart from molecular characterization, we also examined the self-assembly of these polymers. Our observations revealed several higher-order reflections (*q*/q* values of 1, √3, √4, where q* represents the scattering vector of the first-order reflection) for a 0.2-μm-thick **HFSL5** film analyzed using transmission small-angle X-ray scattering (SAXS) (see Figures 2a and 2b). SAXS data were consistent with a hexagonal lattice and further validated through scanning electron microscopy (SEM) after the selective degradation of polylactide block from **HFSL5** (see Figures 2c and 2d). The first peak in the SAXS spectrum was recorded at a *q*-value of 0.0203 Å^–1^, corresponding to the *d*-spacing of 30.9 nm. This finding is in good agreement with the measured pore-to-pore distance of 31.7 ± 3.6 nm in the porous **HFSL5** bulk film (Figure S38). In contrast, the nonfluorinated counterpart **HSL7** exhibited a single peak at a *q*-value of 0.0245 Å^–1^, corresponding to a *d*-spacing of 25.6 nm, with no indication of higher-order peaks (Figure S37). This observation suggests a significant increase in the Flory–Huggins interaction parameter (χ) in the fluorinated polymer, likely due to the pronounced incompatibility between the polymer blocks.^24,25^ To probe the microphase separation behavior in thin film, we performed atomic force microscopy (AFM) for **HFSL5**, which confirmed the presence of nonoriented cylindrical domains (Figures 2e–h). Whereas we observed poorly ordered microstructures for **HSL7** (Figure S39), which was consistent with the respective polymer bulk film morphology findings.

**Figure 2:**
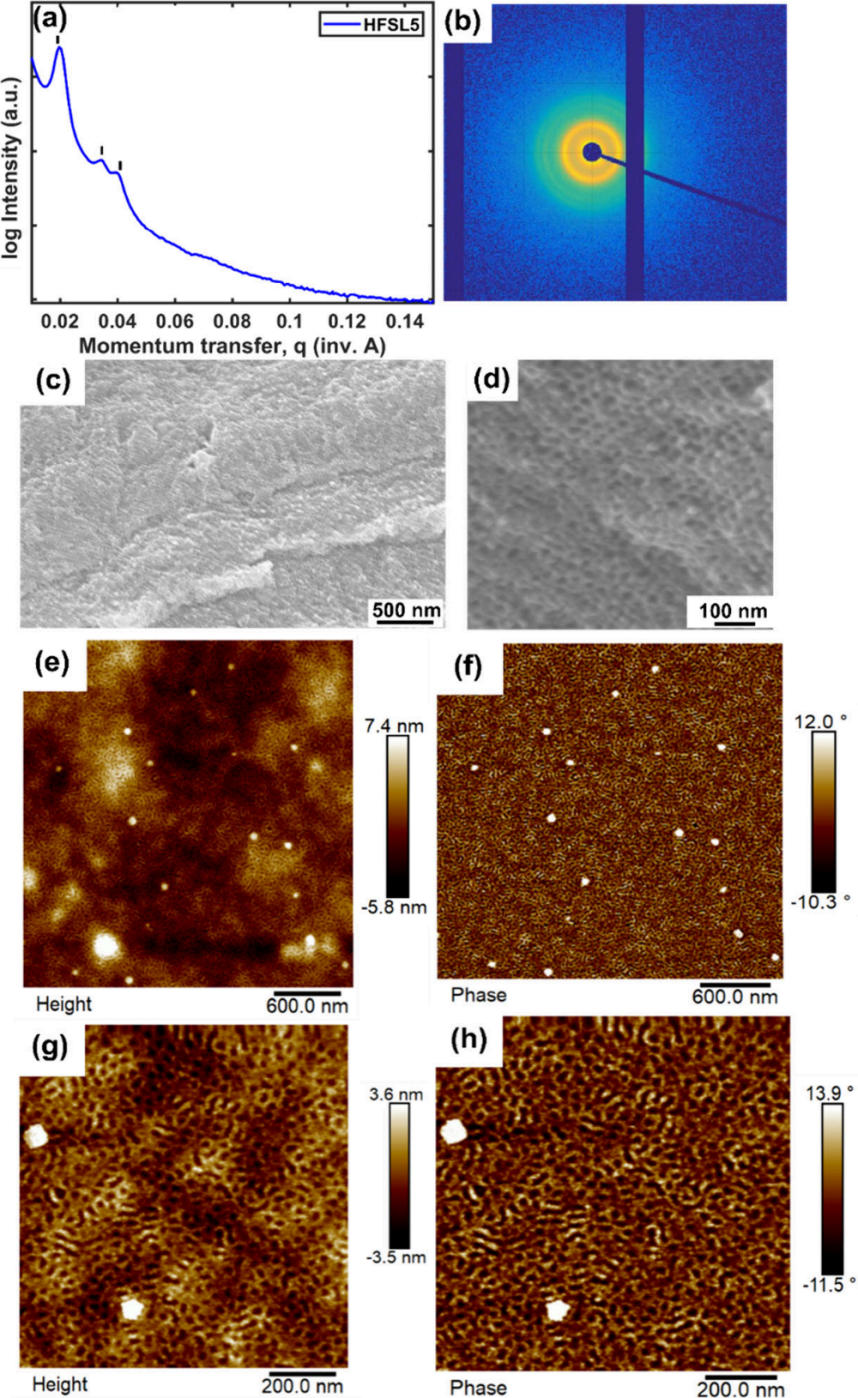
Transmission SAXS (panels (a) and (b)) of **HFSL5** bulk film (0.2 μm thick) suggests expected hexagonal morphology with indicated arrows of possible reflections at *q*/q* spacings of 1:√3:√4. SAXS data are consistent with a hexagonal lattice that is visible in FESEM image (panels (c) and (d)) of the **HSFL5** bulk film after selective degradation of polylactide from it by applying a trifluoroacetic acid etching technique.^15^ Tapping-mode AFM height and phase images of **HFSL5** thin film on silicon substrate confirms the presence of nonoriented cylindrical domain (panels (e)–(h)).

Given that the solution self-assembly of fluorinated block copolymers leads to the formation of diverse nanostructures with attractive architecture, we conducted the self-assembly process on these polymers via dialysis and slow solvent exchange between dimethylformamide and water, as described in Section S6 in the SI. The resultant self-assembled nanostructures were investigated by transmission electron microscopy (TEM) (Figure 3). The TEM images of **HFBuMaSL5** in this figure, with a sequence of fluorophilic–hydrophobic–hydrophilic blocks, revealed the presence of expected *core–shell–corona*(26) morphology (Figures 3a–d) with an average diameter of 134.1 ± 56.4 nm. Spherical particles exhibited a dark central region of much higher contrast, which may correspond to the electron dense fluorophilic core (Figure 3c). Given that the interaction between the fluorinated core and water is less favorable than the interaction between the hydrophobic styrene domain and water, it is anticipated that HFBuMa will localize in the inner region of the aggregates.^26^ The hydrated domain of the polylactide block was visible as a corona in a higher-magnification TEM image (see Figure 3d, as well as Figure S40). Then again, **HFSL5** with a sequence of hydrophobic–fluorophilic–hydrophilic blocks exhibited a markedly different morphology, similar to a soccer-ball-like structure.^27^ These structures were measured to have an average diameter of 108.3 ± 15.3 nm and distinguished by the presence of several dark fluorinated spheres with an average diameter of 25.2 ± 2.5 nm on the surface of the hydrophobic micellar core (Figures 3e–h). This mutual arrangement is anticipated as the fluorinated block (pentafluorostyrene) is affixed to the corona forming polylactide block; however, the corona was not directly visible here. In contrast, its nonfluorinated counterpart **HSL7** exhibits a core–shell–corona morphology (Figure S41), wherein phase separation between polystyrene and polyhexyl acrylate occurs within the sphere, leading to the formation of the core–shell structures. Lastly, to our surprise, **MeFSL2** with a sequence of fluorophilic–hydrophobic–hydrophilic blocks demonstrate no trace of isolated spherical micelles, rather showed formation of network by stacking spherical micelles into long *bamboo-like undulated cylinders*(28) (Figures 3i–k). These cylindrical aggregates span several micrometers in length and display branching points and terminal caps (Figure 3k, Figure S42). It was also evident that the ends of the cylinders were formed by the aggregation of several micelles (Figure S43). The diverse nanostructures are not solely governed by block sequences; additional factors, including block weight fractions and the *T*_g_ value of the core-forming blocks, also play a critical role in modulating these morphologies.

**Figure 3:**
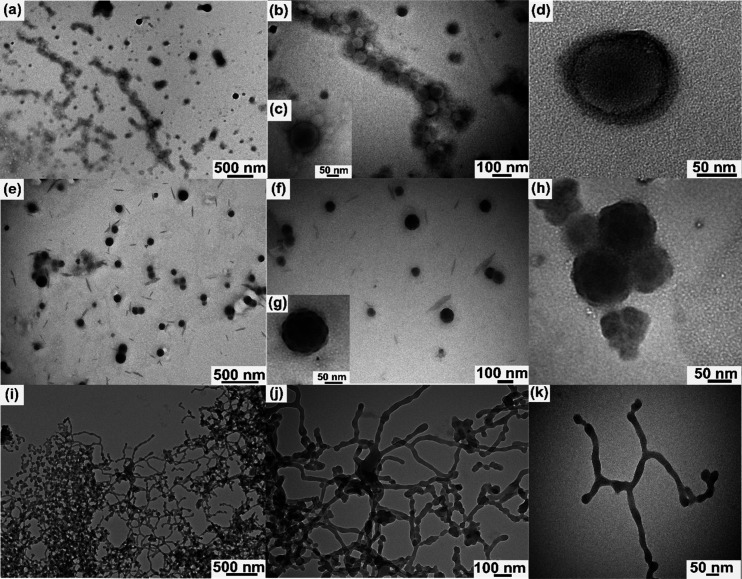
TEM images of solution self-assemblies for polymer candidates (unstained) including (a–d) **HFBuMaSL5**, (e–h) **HFSL5**, and (i–k) **MeFSL2**.

Finally, these polymers were utilized for the removal of PFOA from its aqueous solution. To evaluate the PFOA removal efficiency, 1 mg of each candidate was used to treat a 1 mg L^–1^ aqueous solution of PFOA at a neutral pH for 36 h. The percentage removal efficiency was determined by quantifying the remaining PFOA in the aqueous solution through high-performance liquid chromatography tandem mass spectrometry (HPLC-MS/MS) (details are given in Section S7 in the SI). It was observed that **Candidate 4 MeFSL2** achieved a PFOA removal efficiency exceeding 99.99%, while the nonfluorinated **HSL7** demonstrated no detectable PFOA removal, as shown in Figure 4.

**Figure 4:**
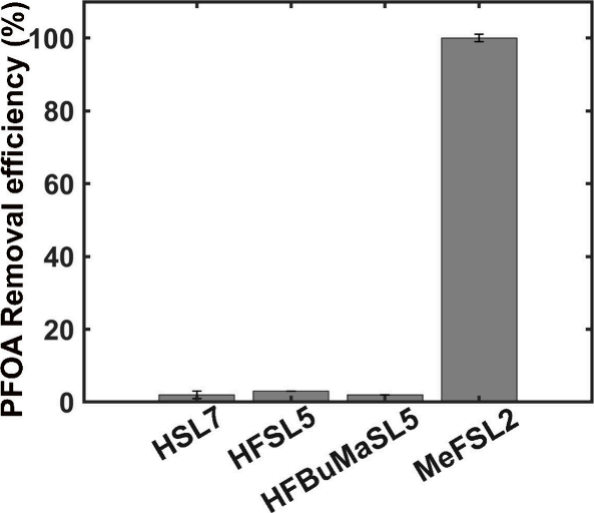
Removal efficiency of 1 mg L^–1^ PFOA by our designed polymer sorbent candidates. Sorbent concentration was maintained at 1 mg/mL.

This observation aligns with the previous report by Whittaker and colleagues,^6^ which demonstrated that a high fluorine content in the polymeric backbone enhances PFOA sorption via favorable C–F**···**F–C interactions. However, we noted a slow PFOA removal rate for **MeFSL2** (33.7% and 99.99% in 6 and 36 h, respectively, determined by HPLC-MS/MS) compared to the polymers developed by Whittaker et al., which achieved over 90% removal within just 5 min (determined by ^19^F NMR diffusion-ordered spectroscopy, DOSY).^6,12^ To better understand the prolonged removal time of **MeFSL2**, we carefully examined its structure. Notably, we observed that each repeat unit of **MeFSL2** contains a single F atom attached to the polymer backbone, while the polymer designed by Whittaker and co-workers^6^ incorporates a higher fluorine content per repeat unit. This suggests that increasing the number of fluorinated segments in the polymer backbone could potentially enhance the PFOA removal rate. Additionally, their polymer design featured a water-soluble hydrophilic segment, poly(oligo(ethylene glycol)methyl ether acrylate,^6^ which likely enhances the kinetics of PFOA sorption. Surprisingly, replacing all protons of the styrene moiety with F atoms in **Candidate 2 HFSL5** did not improve PFOA capture efficiency (Figure 4, Figure S46). Structure analysis revealed that the F atoms are linked to the aromatic ring, not the polymer backbone. Likewise, the incorporation of fluorinated moieties in the side chains of the **Candidate 3 HFBuMaSL5**, did not lead to any notable adsorption (Figure 4, Figure S46). These results are consistent with prior finding^14^ where nondetectable PFOA adsorption was reported by uncharged polystyrene-based anion exchange resin, even when their side chains were functionalized with fluoroalkyl groups. The low PFOA removal observed for **HFSL5** and **HFBuMaSL5** suggests that without electrostatic interaction the fluorophilic influence on the sorption process is reduced, especially when the fluorogenic moieties are positioned in the side chains of the polymers. Another key observation was that altering the fluorophilic/hydrophobic block sequences in **HFSL5** and **HFBuMaSL5** had little to no effect on the PFOA sorption process. Interestingly, TEM images showed no significant structural differences in the self-assembled aggregates of all three fluorinated candidates when dispersed in pure water (see Figures S44 and S45). We suggest that MD simulations in future studies could provide valuable molecular-scale insights into how the location of the fluorinated segment within the polymer structure affects the strength and stability of C–F**···**F–C interactions.

In summary, our designed triblock copolymer with a fluorinated moiety inserted into the polymer backbone demonstrated a near-complete removal of PFOA from the aqueous solution. In contrast, other polymer candidates, which lacked a fluorogenic moiety or incorporated a fluorinated segment in the side chains, exhibited poor PFOA removal. These results highlight an important design consideration: the C–F**···**F–C interactions, which play a key role in PFOA sorption, are more effective when F atoms are attached to the polymer backbone, rather than to the side chains. However, it is important to note that, while **MeFSL2** effectively removed long-chain PFAS through fluorophilic interactions, its efficiency may be insufficient for removing PFAS at low parts per billion concentrations in aqueous environments or in the presence of large number of co-contaminants. Given that both electrostatic and fluorophilic interactions influence adsorption,^5,12,14^ our future work will focus on enhancing the sorption efficiency of polymer candidates and measuring their complete adsorption isotherms for both short- and long-chain PFAS at environmentally relevant concentrations. This will involve further modification of the polymer system, incorporating additional F atoms into their backbone, and introducing electrostatic interactions by integrating cationic quaternized ammonium groups.

## Supporting Information Available

Experimental methods (sorbents candidate synthesis strategies), characterization methods including ^1^H and ^19^F NMR, electrospray ionization mass spectrometry (ESI-MS), SEC, thermal analysis (TGA, DSC), SAXS, FESEM, AFM, TEM, polymer solubility, percentage (%) PFOA removal by polymer sorbents (PDF)
