# Supplementary material for: Fluorinated Block Copolymer: An Important Sorbent Design Criteria for Effective PFOA Removal from Its Aqueous Solution
Source: ACS Appl Polym Mater. 2025 Feb 3;7(3):1187–93. doi: 10.1021/acsapm.4c03792 (PMC11833762; doi:10.1021/acsapm.4c03792)
Supplement: Supplementary file 1 — ap4c03792_si_001.pdf [file ap4c03792_si_001.pdf]

Supporting Information

Fluorinated Block Copolymer: An Important Sorbent Design Criteria for Effective PFOA Removal from its Aqueous Solution

Sadifa Anjum,[a,#] Michael Arik,[a,#] Arya Patel,[a,#] Nyalah Abasali,[a] Laying Wu,[b] Amrita Sarkar [a,c*]

[a]*Department of Chemistry & Biochemistry, Montclair State University, Montclair, NJ 07043, USA.*
[b]*College of Science & Mathematics, Montclair State University, Montclair, NJ 07043, USA*
[c]*Sokol Institute for Pharmaceutical Life Sciences, Montclair State University, Montclair, NJ 07043, USA*
**Corresponding author: sarkara@montclair.edu, #Equal contribution*

Table of Contents

Section 1. Materials & Methods……………………………………………….……S2-S4
Section 2. Polymer Sorbent Candidates Synthesis & Characterization…………S5-S33
Section 3. Thermal Analysis (TGA & DSC)……………………………………….S34-S35
Section 4. Bulk Polymer Self-Assembly (SAXS, SEM)……………..…………….S36-S37
Section 5. Polymer Thin Film Phase & Topography Image (AFM)…………………………………………………………….………..….S38
Section 6. Polymer Aqueous Solution Self-Assembly (TEM)……………………..S39-S43
Section 7. Percentage Removal of PFOA by Polymer Sorbent Candidates………S44-S45
Section 8. References……………………………………………………………….S45-S46

Section 1. Materials & Methods

Materials: Trifluoroacetic acid (99%, Alfa Aesar), aluminum oxide (99%, Thermo Scientific Chemicals), styrene (99%, stabilized, Thermo Scientific Chemicals), 2,3,4,5,6-pentafluorostyrene (98%, stabilized with TBC, TCI America), hexyl acrylate (96%, stabilized with HQ, TCI America), 2,2,3,4,4,4-hexafluorobutylmethacrylate (Thermo Fischer), methyl-2-fluoroacrylate (95% stabilized with 1% BHT, Thermo Scientific Chemicals), benzoic acid (99.5%, Aldrich), 1,8-Diazabicyclo[5.4.0]undec-7-ene (98%, Thermo Scientific Chemicals), dimethyl formamide (>99.98%, Fisher), tris[2-(dimethylamino)ethyl]amine (98%, Thermo Scientific Chemicals), copper(I) bromide (99.998%, Thermo Scientific Chemicals), copper (I) chloride (99.99%, Thermo Scientific Chemicals), tin(II) ethyl hexanoate (92.5-100%, Millipore sigma), 3,6-Dimethy-1,4-dioxane-2,5-dione (99%, Acros Organics), 2-hydroxyethyl-2-bromoisobutyrate (95%, Aldrich), cyclohexylamine (99%, Alfa Aesar), 3,5-bis(trifluoromethylphenyl isothiocyanate) (99%, Thermo Scientific), anhydrous chloroform (stabilized with amylene, 99.9%, Acros Organics), anhydrous THF (inhibitor free, 99%, Fisher), and methanol (>99.8%, VWR). All reagents were used without further purification unless otherwise noted.

Analytical Characterization

Nuclear Magnetic Resonance (NMR): All NMR experiments were performed at 500 MHz Bruker Avance III HD NMR spectrometer equipped with 5 mm H/F-11B selective probe. All spectra were recorded either in $CDCl_3$ or DMSO-d6. The number of scans was set to 64.

Mass Spectrometry (MS): High resolution MS was performed using a Thermo Orbitrap MS, which is connected to Thermo Vanquish UPLC system via an electrospray ionization (ESI) source as interface. Mobile phase is 50% Acetonitrile + 0.2% Formic acid (v/v) at 200ul/min for the direct injection. The conditions of MS analysis are as follows: the mass spectrometer parameters are positive ion mode, ion spray voltage at 4000 V, capillary voltage at 41V, capillary temperature at 275 °C, sheath gas flow rate at 40 psi and auxiliary gas flow rate at 20 psi. The scan spectra include m/z 400 to 2000. The instrument m/z values were calibrated using the manufacturer's ESI Positive Ion calibration mixture. All data analysis was performed using Thermo Xcalibur™ software. Accurate mass measurements were performed at high resolution (resolving power of 60,000 FWHM at m/z 400).

Size Exclusion Chromatography (SEC): Polymers number and weight-average molecular weights, and dispersity were determined by a Waters Alliance e2695 gel permeation chromatography or size exclusion chromatography equipped with a 717 plus autosampler, a 1525 HPLC pump, and a 2414 refractive index (RI) detector. Columns of PLgel 5 µm guard, PLgel 10 µm MIXED-B and PLgel 5 µm MIXED-C were set at temperature of 30 °C. The columns were calibrated with narrow polystyrene (PS) standards in the molecular weight range of 580 to 371,100 Da. The measurements were carried out with the sample concentrations of 1 mg/mL eluted by THF with a flow rate of 0.5 mL/min. Instrument operation and data analysis were performed using Empower software.

Differential Scanning Calorimetry (DSC) and Thermogravimetry Analysis (TGA): Thermal analyses of the synthesized polymers were performed with a Differential Scanning

Calorimeter TA Instrument Discovery DSC in a temperature range of 25 to 300 ºC at a heating rate of 10 ºC min^{-1} under a nitrogen flow of 60 mL min^{-1}. The glass transition temperature (T_g) was determined from the second heating trace and is reported as the midpoint of the thermal transition. Thermal degradation of the polymers was investigated by thermogravimetric analysis (TGA) performed with a TA Instruments Discovery TGA. Measurements were conducted from 30 to 600 ºC at a rate of 10 ºC min^{-1} in a nitrogen flow of 60 mL min^{-1}.

Small angle X-ray scattering (SAXS): SAXS experiments were conducted using a SAXSLab Ganesha system at the South Carolina SAXS Collaborative. A Xenocs GeniX3D microfocus source was used with a Cu target to generate a monochromatic beam with 1.54 Å wavelength. The instrument was calibrated using National Institute of Standards and Technology (NIST) reference material, 640c silicon powder with the peak position at 2θ = 28.44º where 2θ is the total scattering angle. A Pilatus 300 K detector (Dectris) was used to collect the two-dimensional (2D) scattering patterns with the incident beam normal to the planar sample surface. 2D images were azimuthally integrated into 1D data of intensity versus momentum transfer. All data were acquired after 20-30 minutes measurement with an X-ray flux of ~4.1 M photons per second incident upon the sample.

Scanning Electron Microscopy (SEM): The lactide degraded porous polymer bulk film was imaged by Zeiss Sigma VP scanning electron microscopy (FESEM). Polymer samples were mounted on brass shims using carbon adhesive (Electron Microscopy Sciences) and imaged normal to the planar film surface. Samples were sputter coated with gold–palladium alloy using a Cressington 108 Manual Sputter Coater. Top-view images of polymer films were acquired using an acceleration voltage of 5 keV using an in-lens secondary electron detector. The working distance was maintained at ~3-4 mm.

Atomic Force Microscopy (AFM): Polymer samples (5 wt% solution in chloroform) were spin coated onto Si wafer at 1500 rpm for 30 seconds and annealed at 120 °C for 20 minutes before imaging. The silicon wafers were subsequently cleaned using ethanol and DI water in an ultrasonic bath and were dried by a nitrogen stream before use. Phase and topography images of the coated films were obtained using a Bruker Dimension Icon and Bruker MultiMode AFM operating in tapping mode. Tip used for imaging: RTESPA-150 (Bruker) with a nominal F0 of 150 kHz and spring force constant, k = 6 N/m.

Transmission Electron Microscopy (TEM): TEM images were acquired using a Hitachi H-7500 tungsten/LaB6TEM operated at 100 kV. The resultant dialyzed content (one drop) was placed on a carbon coated copper grid (Electron Microscopy Sciences, USA), and then placed on a piece of filter paper to remove excess solvent, and air dried before imaging. Samples were not stained before imaging.

High-performance liquid chromatography tandem mass spectrometry (HPLC-MS/MS): The percentage (%) removal of Perfluorooctanoic acid (PFOA) from its aqueous solution using the polymer sorbent candidates were measured via HPLC-MS/MS. Prior to analysis, sorbent treated samples were diluted to fall within the calibration curve, then transferred to polypropylene autosampler vials via polypropylene pipettes. 1 μL injections of samples were run on a Sciex ExionLC followed by an Applied Biosystems/SCIEX Triple Quadrupole 7500 mass spectrometer using an Xbridge HPLC BEH C18 2.5 µm 2.1x50 mm column coupled with an Acquity UPLC Hybrid Reversed Phase 2.1x50 mm isolator column. The column

temperature was held at 40°C. Separation of the analytes are achieved using gradient elution chromatography. 2mM ammonium acetate in H_2O was used as mobile phase A and HPLC grade Acetonitrile was used as mobile phase B. The LC method steps are given in **Table S1**. After elution from the HPLC column, the analyte PFOA was detected with a turbo ion spray triple quadrupole mass spectrometer using multiple reaction monitoring (MRM) in negative ionization mode and quantitated using isotope dilution, see **Tables S2** and **Table S3** for MRM parameters.

Table S1: LC Step Table

Step	Time (min)	Flow (mL/min)	A (%)	B (%)
0	0	0.300	95	5
1	0.2	0.300	95	5
2	1.0	0.300	50	50
3	8.0	0.300	5	95
4	9.5	0.300	0	100
5	10	0.300	95	5

Table S2: Exion UPLC/SCIEX 7500A MRM Parameters, where the primary (quantitation) ion transition is denoted by "-1" and secondary (confirmation) ion transition is denoted by "-2"

Compound	Internal Standard Used for Quantitation	Q1 Mass (Da)	Q3 Mass (Da)	Dwell Time (ms)	EP (V)	CE (V)	CXP (V)
Experiment 1, Ion Spray Voltage (IS): 4000							
PFOA-1	13C8-PFOA	413	369	60	-10	-14	-20
Experiment 2, Ion Spray Voltage (IS): 2000							
PFOA-2	13C8-PFOA	413	169	60	-10	-26	-12
13C8-PFOA	---	421	376	60	-10	-15	-24

Table S3: Additional MRM parameters

CUR:	40 psi
GS1:	40 psi
GS2:	70 psi
IS:	See tables above
TEM:	300°C
CAD:	14
CEM	1900 V (variable)

Section 2. Polymer Candidates Synthesis and Characterizations

Sorbent Candidate 1 HSL

Control Polymer poly(hexylacrylate)-*block*-(styrene)-*block* (lactide) (HSL)

Step 1. Polystyrene (PS) macroinitiator synthesis

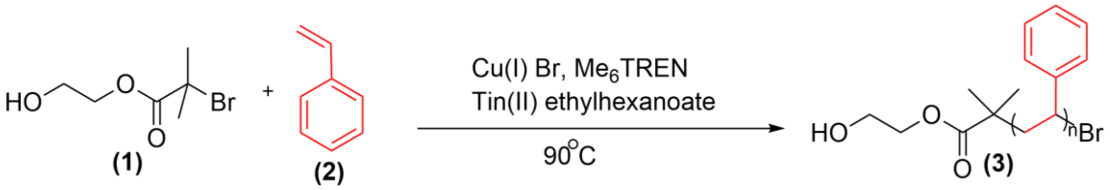

Scheme S1. Synthesis of Polystyrene **(**PS) macroinitiator **(3)**.

Polystyrene, PS **(3)** macroinitiator was synthesized via Activator Regenerated Electron Transfer Atom Transfer Radical Polymerization (ARGET ATRP) following procedure described elsewhere.[1] Briefly stated, 110 mL (960 mmol) alumina column treated inhibitor free styrene monomer **(2)** was mixed with 464 µL (3.2 mmol) initiator 2-hydroxyethyl-2-bromoisobutyrate **(1)** in a Schlenk flask. The flask was degassed via three cycles of freeze-pump-thaw (FPT). Separately, a catalyst stock solution of 2.3 mg (0.016 mmol) Cu(I)Br, 90 µL (0.336 mmol) Me_6TREN ligand and 104 µL (0.32 mmol) tin (II) ethyl hexanoate reducing agent prepared in toluene was added into the flask under nitrogen gas flow. The reaction flask was placed in a pre-heated oil bath set at 90 °C and the reaction continued for 26 hours. Upon completion, the reaction medium was diluted with tetrahydrofuran (THF) and precipitated in 5x excess chilled methanol. The obtained PS was washed with chilled methanol twice and dried at 60 °C under vacuum for 48 hours. Product **(3)** was characterized by ^{1}HNMR (**Figure S1**) and SEC (**Figure S2**). In the ^{1}HNMR spectrum (**Figure S1**), broad peaks are found at the range of 6.3-7.5 ppm, and 1.3-2.5 ppm, responsible for aromatic (assigned by a) and aliphatic region (assigned by c and d), respectively. Signal of initiator methylene proton (-CH_2, assigned by b) was noted at 3.5-3.8 ppm. Signal at 4.35-4.65 was assigned to the proton (c') on the carbon adjacent to bromine of the growing PS chain. Chain end-functionality is calculated as 97%. The number average molas mass (M_n) was estimated as 12.4k gmol^{-1} by ^{1}HNMR end group analysis. The M_n 13.6k gmol^{-1}, determined by SEC (**Figure S2**) found to be comparable with the M_n estimated value by ^{1}HNMR. Narrow disparsity (Đ) 1.09 indicates controlled polymerization.

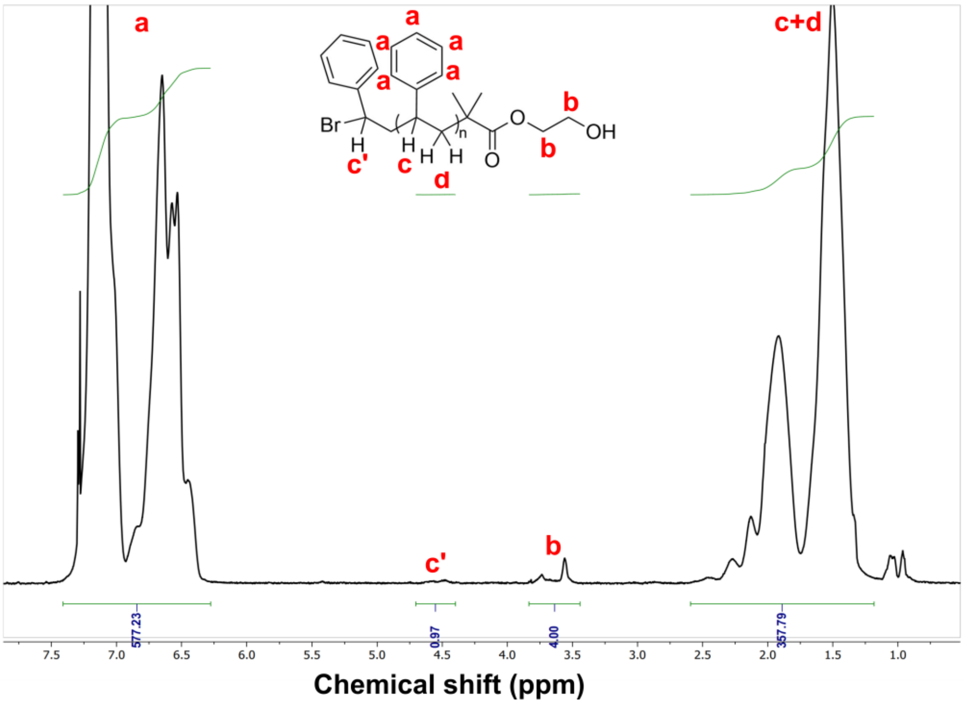

Figure S1. ^{1}HNMR of PS macroinitiator (**3**), spectrum was obtained in $CDCl_3$. A strong peak at 7.26 ppm was found due to the residual $CHCl_3$ in the NMR solvent.

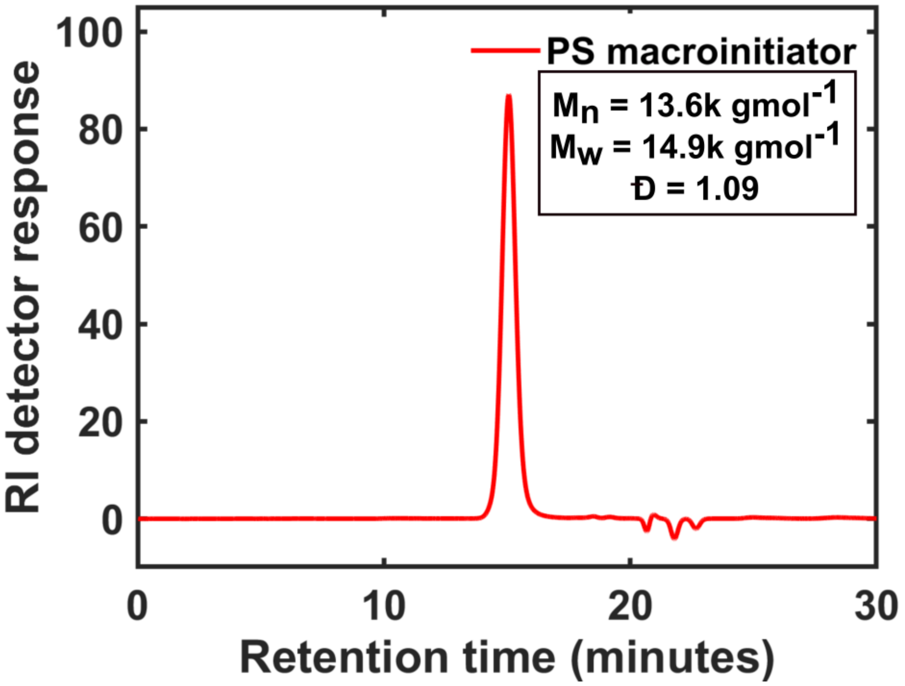

Figure S2. SEC for macroinitiator PS **(3)**, performed in THF and calibrated with polystyrene (PS) standards.

Step 2. Poly(hexylacrylate-*block*-styrene) (HS) diblock synthesis

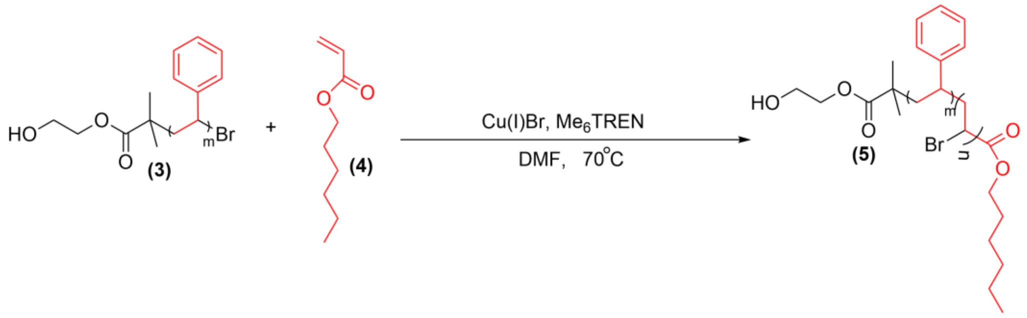

Scheme S2. Synthesis of HS diblock polymer **(5)**.

HS diblock polymer **(5)** was synthesized via atom transfer radical polymerization (ATRP) following procedure described elsewhere.[2-4] Briefly stated, 20g (1.61 mmol) PS macroinitiator **(3)** was dissolved in 60 mL dimethyl formamide (DMF). 34.3 mL (193 mmol) monomer hexyl acrylate **(4)** was added into the reaction mixture kept in a Schlenk flask. The flask was degassed via three cycles of FPT. Separately, a catalyst stock solution of 115 mg (0.805 mmol) Cu(I)Br and 223 µL (0.805 mmol) Me_6TREN ligand prepared in DMF was added into the flask via a syringe under flowing nitrogen gas. The reaction flask was placed in a pre-heated oil bath set at 70 °C and the reaction continued for 72 hours. Upon completion, the reaction medium was diluted with DMF and precipitated in 5x excess chilled methanol. The obtained HS diblock **(5)** was washed with chilled methanol twice and dried for 48 hours at 40 °C under vacuum. The product **(5)** was characterized by ^{1}HNMR (**Figure S3**) and SEC (**Figure S4**). In the ^{1}HNMR (**Figure S3**), peaks found at 1.80-2.05, and 2.26 ppm are associated with $-CH_2$ and -CH(Br) on the backbone of hydrophobic poly(hexylacrylate), assigned as c and b, respectively. Likewise, the signal for $-CH_2$ protons for side chain (peak d) was found at 4.01 ppm. M_n estimated for HS diblock polymer from the ^{1}HNMR was 28.5k gmol^{-1}. The M_n of the hexyl acrylate block was calculated from the ^{1}HNMR spectrum of the HS diblock polymer based on the ratio of integration values of the aromatic protons (a) in the polystyrene block and the ethyl hydrogen in the hexyl acrylate block. The monomodal SEC curve (**Figure S4**) suggests the formation of diblock polymer without any homopolymerization.

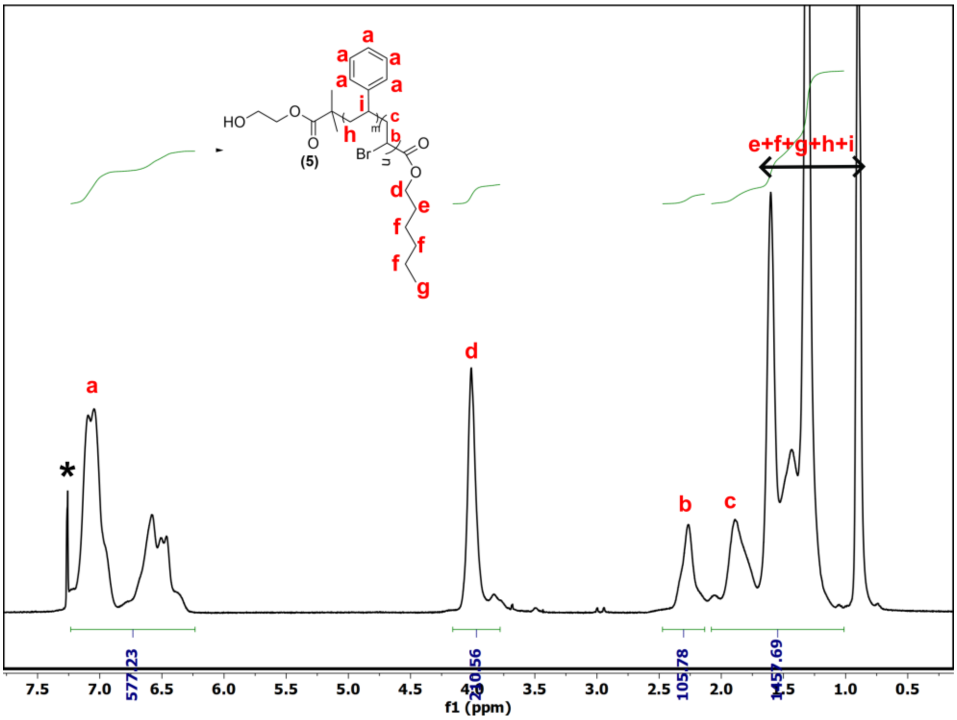

Figure S3. ^{1}HNMR of HS diblock polymer (**5**), spectrum was obtained in $CDCl_3$. A strong peak at 7.26 ppm was found due to the residual $CHCl_3$ in the NMR solvent, marked by *.

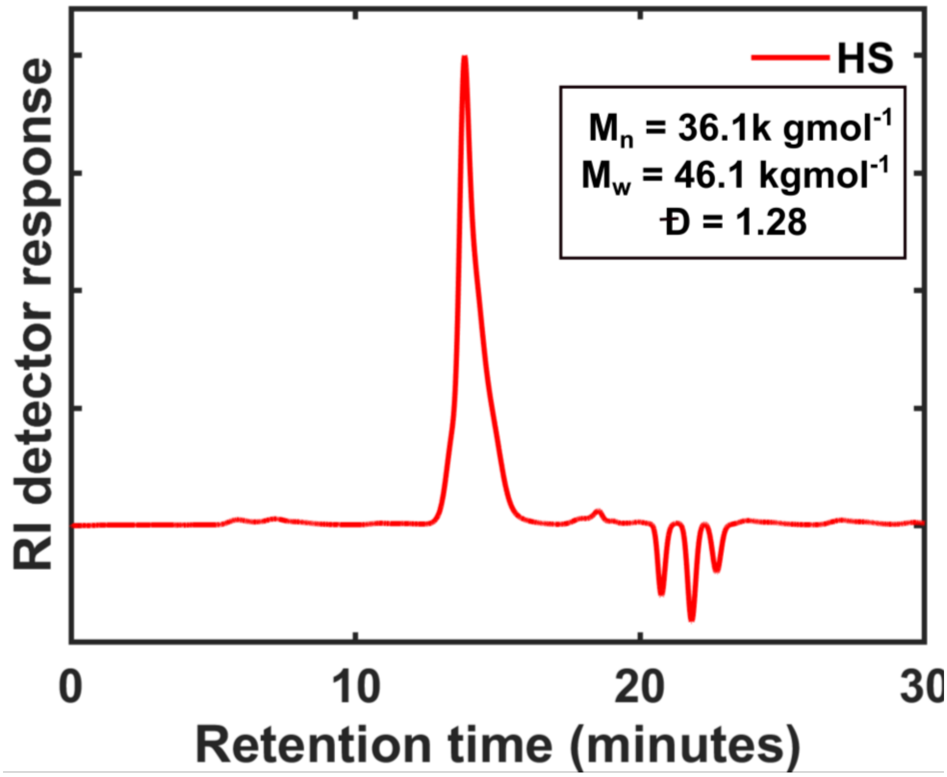

Figure S4. SEC for HS diblock polymer **(5)**, performed in THF and calibrated with PS standards.

Step 3. Poly(hexylacrylate-*block*-styrene-*block*-lactide) (HSL) triblock polymer synthesis

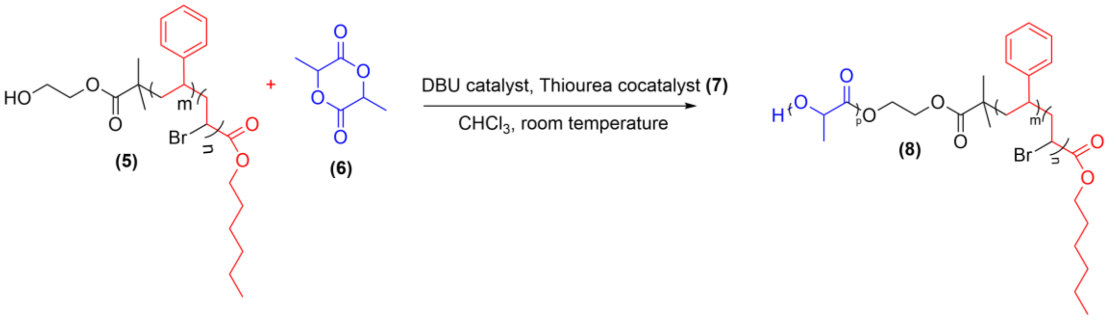

Scheme S3. Synthesis of HSL triblock polymer **(8)**.

HSL triblock polymer **(8)** was synthesized via organolactide ring opening polymerization (ROP) following procedure described elsewhere.[1] Briefly stated, 5g (0.176 mmol) HS diblock polymer **(5)** was dissolved in 10 mL anhydrous chloroform. This was mixed with 2.5 g (17.6 mmol) lactide monomer **(6)** in a flame-dried Schlenk flask. Immediately after that 35 µL 1,8-diazabicyclo[5.4.0]undec-7-ene (DBU) (0.236 mmol) and 87.3 mg thiourea cocatalyst **(7)** (0.236 mmol) were added into the reaction flask. The reaction continued for 1 hour at room temperature under flowing nitrogen gas. The reaction was stopped by adding 28.8 mg benzoic acid (0.236 mmol). The crude mixture was precipitated in 5x excess chilled methanol. The obtained HSL triblock polymer **(8)** was washed with chilled methanol twice and dried overnight at 40 °C under vacuum. The product **(8)** was characterized by ^{1}HNMR (**Figure S5**) and SEC (**Figure S6**). Along with the other signals originating from HS diblock, a broad signal observed in the ^{1}HNMR (**Figure S5**) at 5.10-5.24 ppm, that is linked to the -CH protons (peak h) for polylactide. A series of HSL polymers with the M_n range of 20-40k gmol^{-1} was synthesized. The M_n for a representative HSL7 triblock polymer estimated from the NMR spectrum is 32k gmol^{-1}. Clear shift of the monomodal SEC trace (**Figure S6**) for HSL towards higher molecular weight (lower retention time) suggests the successful synthesis of triblock polymer. No overlapping between the SEC traces of block polymers (HS and HSL) and PS macroinitiator were observed. The SEC estimated $M_{n,total}$ 40k gmol^{-1} is higher than NMR estimated value 32k gmol^{-1}, possibly due to the differences in hydrodynamic volume of acrylate, lactide and calibration agent PS.

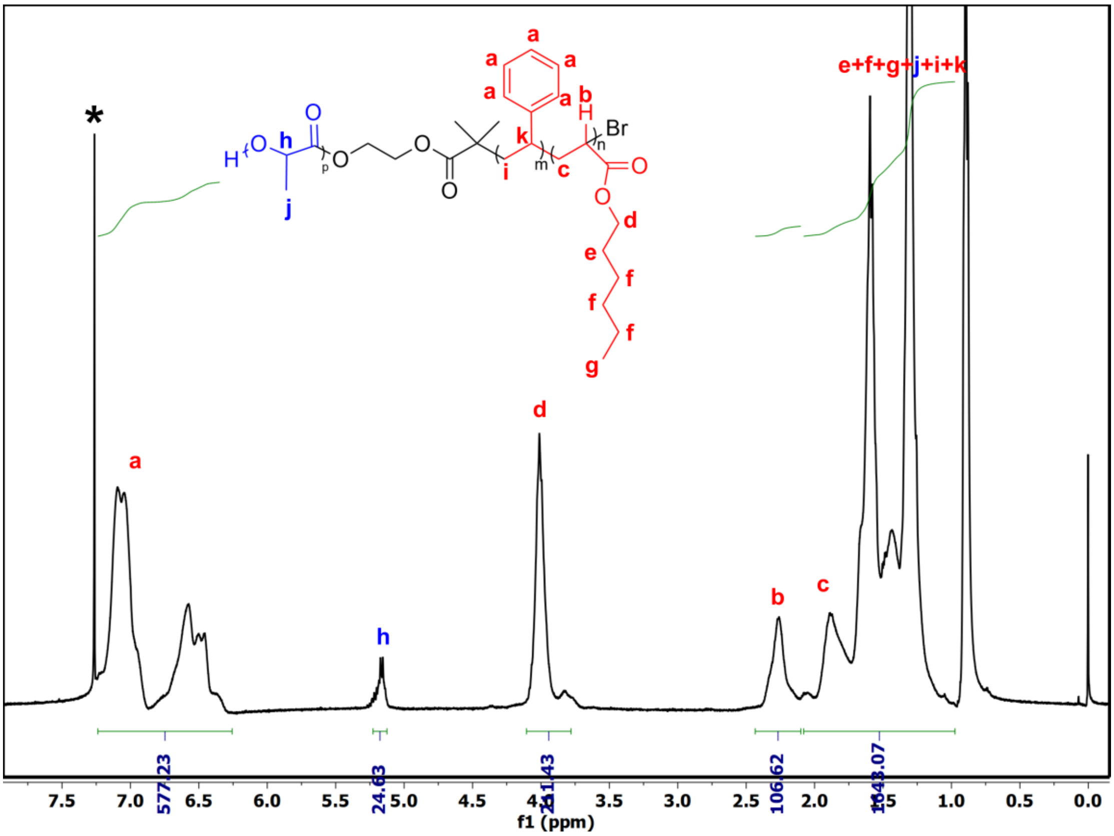

Figure S5. ^{1}HNMR of HSL triblock polymer (**8**), spectrum was obtained in $CDCl_3$. A strong peak at 7.26 ppm was found due to the residual $CHCl_3$ in the NMR solvent, marked by *.

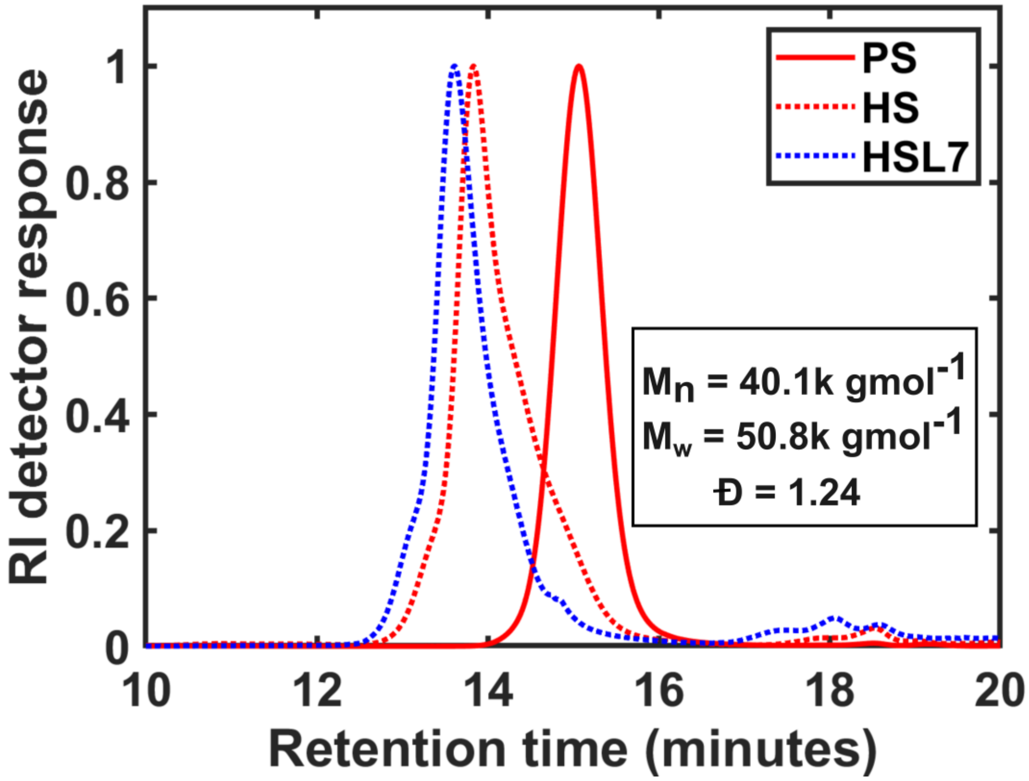

Figure S6. SEC profile for triblock polymer HSL7 **(8)** along the macroinitiator PS **(3)** and the diblock HS **(5).**

Scheme S4. Co-catalyst bis(3,5-trifluoromethyl)phenylcyclohexyl thiourea (7) synthesis.

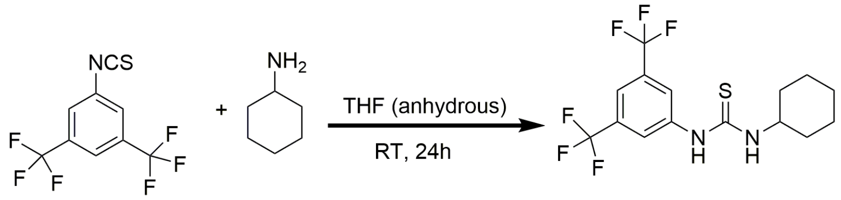

Scheme S4. Synthesis of cocatalyst bis(3,5-trifluoromethyl)phenylcyclohexyl thiourea **(7)**.

Cocatalyst (**7**) was synthesized following procedure described elsewhere.[1] Briefly stated, 25 mL 3,5-bis(trifluoromethyl)phenylisothiocyanate and 75 mL anhydrous THF were combined in a round bottom flask that was previously dried using a heat-gun and contained a magnetic stir bar. 15.7 mL cyclohexylamine was added to this stirring solution dropwise *via* a syringe. The reaction continued at room temperature for 24 h under constant nitrogen gas flow and then THF was removed using a rotary evaporator. White residue was recrystallized twice from chloroform and dried under vacuum at 40 °C for 48 h. The product **(7)** was characterized by ^{1}HNMR (**Figure S7**) and ESI-MS (**Figure S8**).

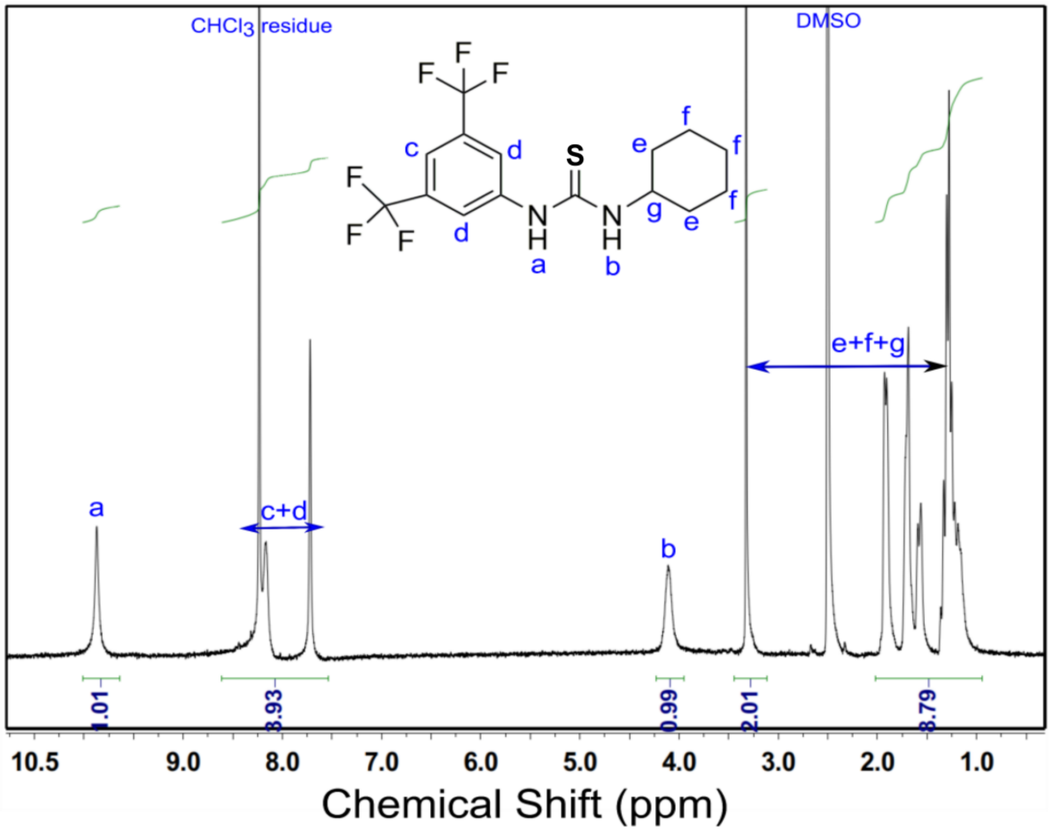

Figure S7. ^{1}HNMR of cocatalyst (**7**), spectrum was obtained in DMSO-d6.

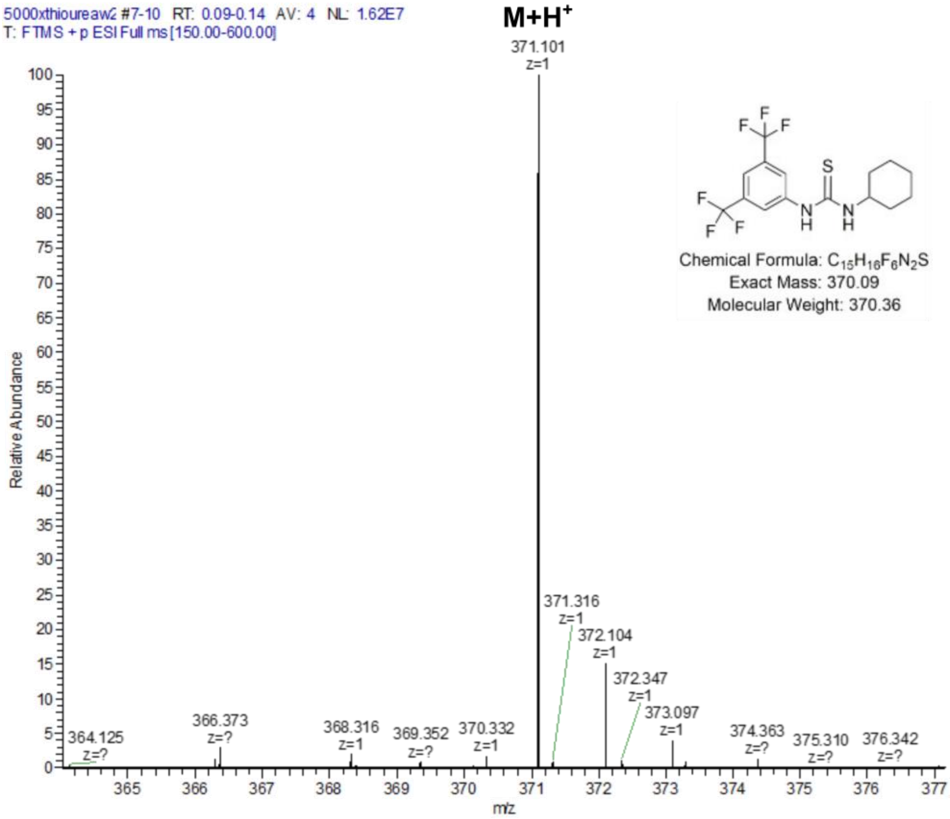

Figure S8. ESI-MS for cocatalyst (**7**)

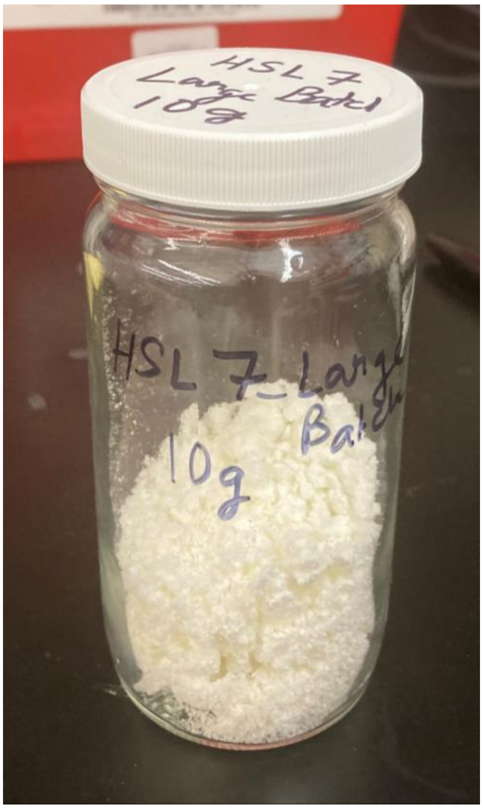

Figure S9. A photograph of the sorbent **Candidate 1** HSL7, used in the PFOA removal experiment.

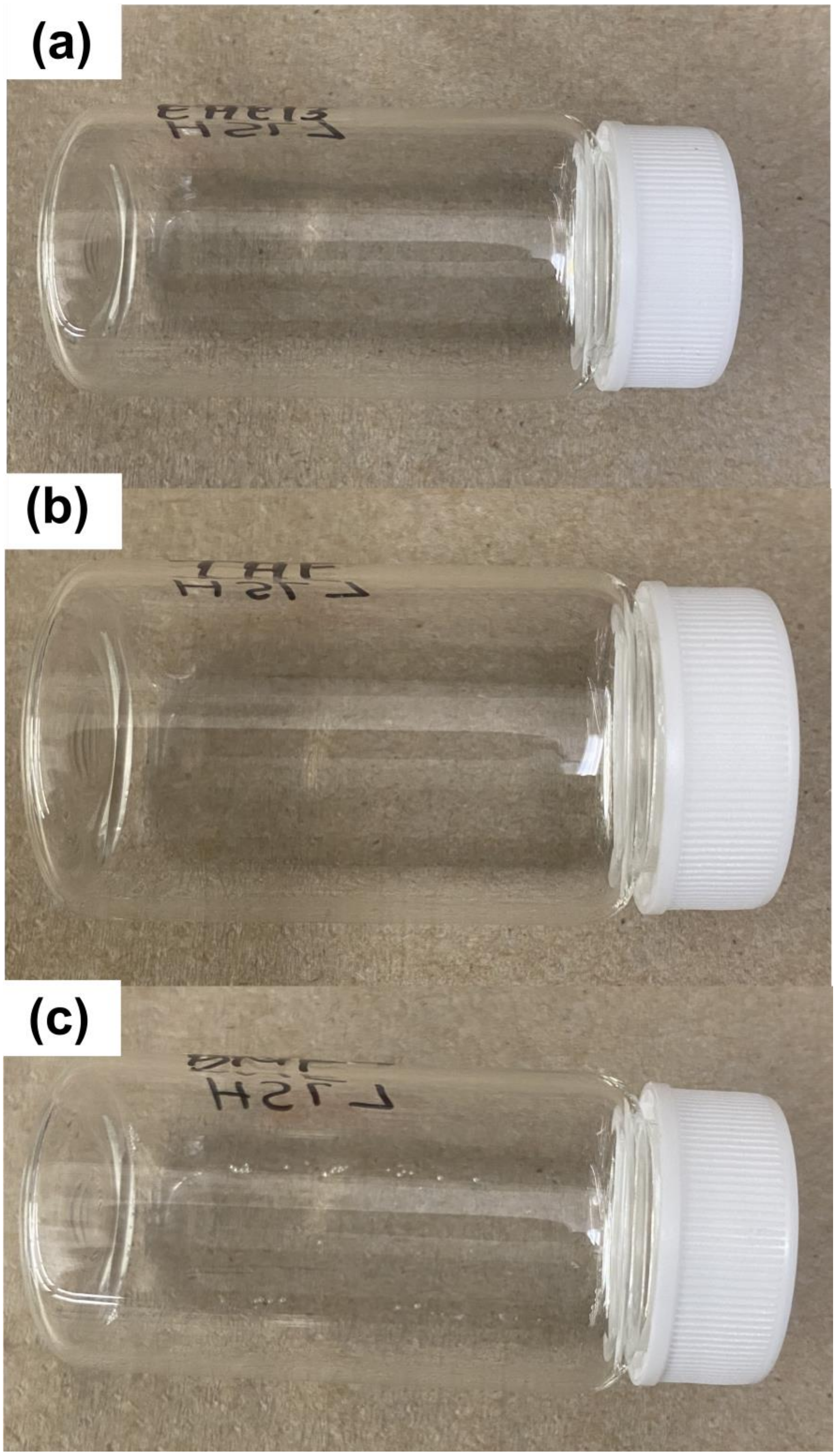

Figure S10. A photograph of 1 wt/vol% solution of **Candidate 1** HSL7 in chloroform (a), THF (b) and DMF (c). Photographs were taken after 2 minutes vortex of polymer solutions at room temperature. All solutions demonstrate clear appearance and confirm excellent solubility of HSL7 in common organic solvents.

Sorbent Candidate 2 HFSL

Sorbent Candidate poly(hexylacrylate)-*block*-(2,3,4,5,6-pentafluorostyrene)-*block* (lactide) (HFSL).

Step 1. Poly(pentafluorostyrene) (PFS) macroinitiator synthesis

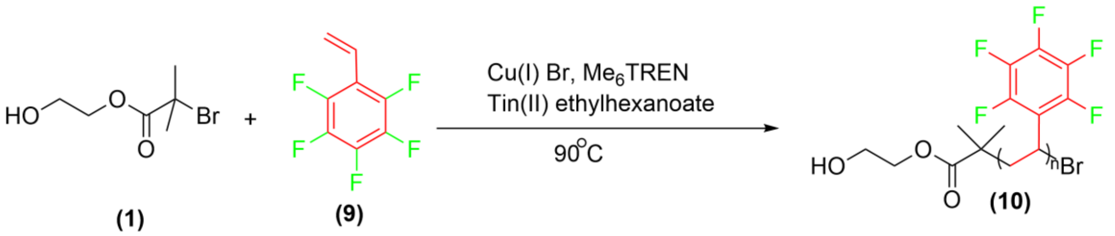

Scheme S5. Synthesis of PFS macroinitiator **(10)**.

Polypentafluorostyrene, PFS **(10)** macroinitiator was synthesized via ARGET ATRP. 35.5 mL (258 mmol) alumina column treated inhibitor free 2,3,4,5,6-pentafluorostyrene monomer **(9)** was mixed with 35 mL DMF. After that, 311 µL (2.15 mmol) initiator 2-hydroxyethyl-2-bromoisobutyrate **(1)** was added to the Schlenk flask. The flask was degassed via three cycles of FPT. Separately, a catalyst stock solution of 1.5 mg (0.011 mmol) Cu(I)Br, 60 µL (0.225 mmol) Me_6TREN ligand and 70 µL (0.215 mmol) Sn (II) ethyl hexanoate reducing agent prepared in toluene was added into the flask under nitrogen gas flow. The reaction flask was placed in a pre-heated oil bath set at 90 ºC and continued for 48 hours. Upon completion, the reaction medium was diluted with THF and precipitated in 5x excess chilled methanol. The product was washed with chilled methanol twice and dried overnight at 60 ºC under vacuum. The product **(10)** was characterized by ^{1}HNMR (**Figure S11**) and SEC (**Figure S12**). Since all aromatic protons of pentafluorostyrene are replaced with fluorine, no proton signal responsible for aromatic protons appears at ~7 ppm in ^{1}HNMR (**Figure S11**). The sum of the aliphatic -CH-CH_2 protons for PFS (peaks d and e) was noted in the range of 1.70-3.0 ppm. The protons signal from initiator (peak a and b) was observed at 3.66-4.10 ppm. The number average molas mass (M_n) was estimated as 5.8k gmol^{-1} by ^{1}HNMR end group analysis. The M_n 6.6k gmol^{-1} determined by SEC, agrees to the M_n of 5.8k gmol^{-1} estimated by NMR. Additionally, monomodal SEC curve (**Figure S12**) with narrow Đ of 1.13 indicates controlled polymerization. We synthesized various PFS macroinitiator (**10**) with a range of different M_n. For example, a high molecular weight PFS was synthesized that we were unable to determine the molar mass via HNMR, as it was hard to evaluate the integration area of the signal (e') found at the 4.62-4.98 ppm.[5] However, the monomodal SEC suggests a controlled synthesis of high molecular weight PFS (**Figure S13**).

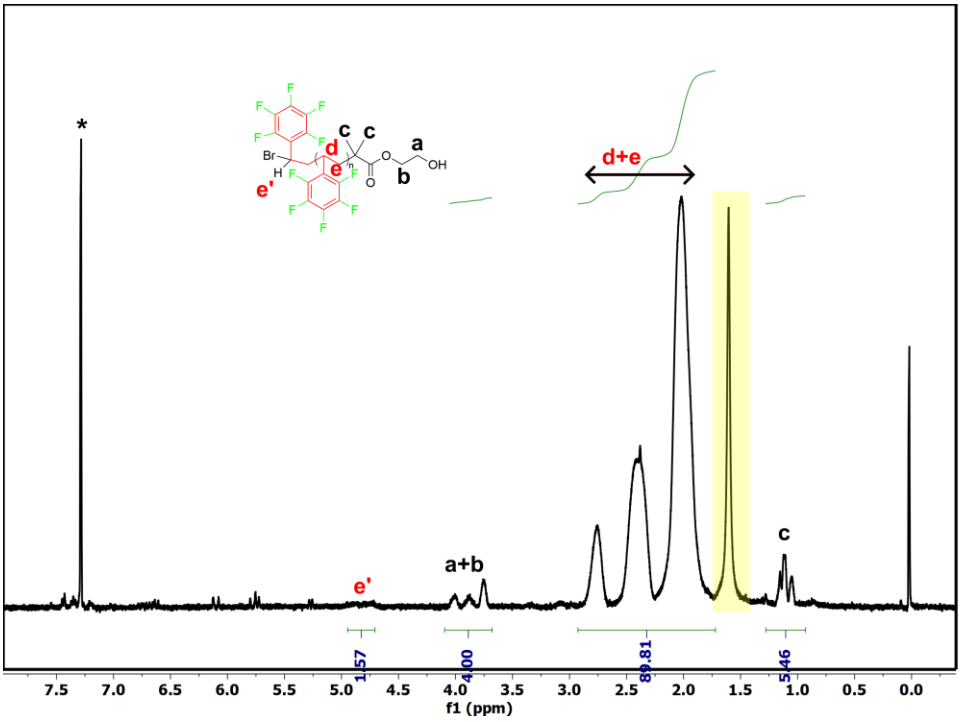

Figure S11. ^{1}HNMR of PFS macroinitiator (**10**), spectrum was obtained in $CDCl_3$ (marked by *). A strong peak at 7.26 ppm was found due to the residual $CHCl_3$ in the NMR solvent. A trace of water was found at 1.55 ppm, highlighted in yellow color.

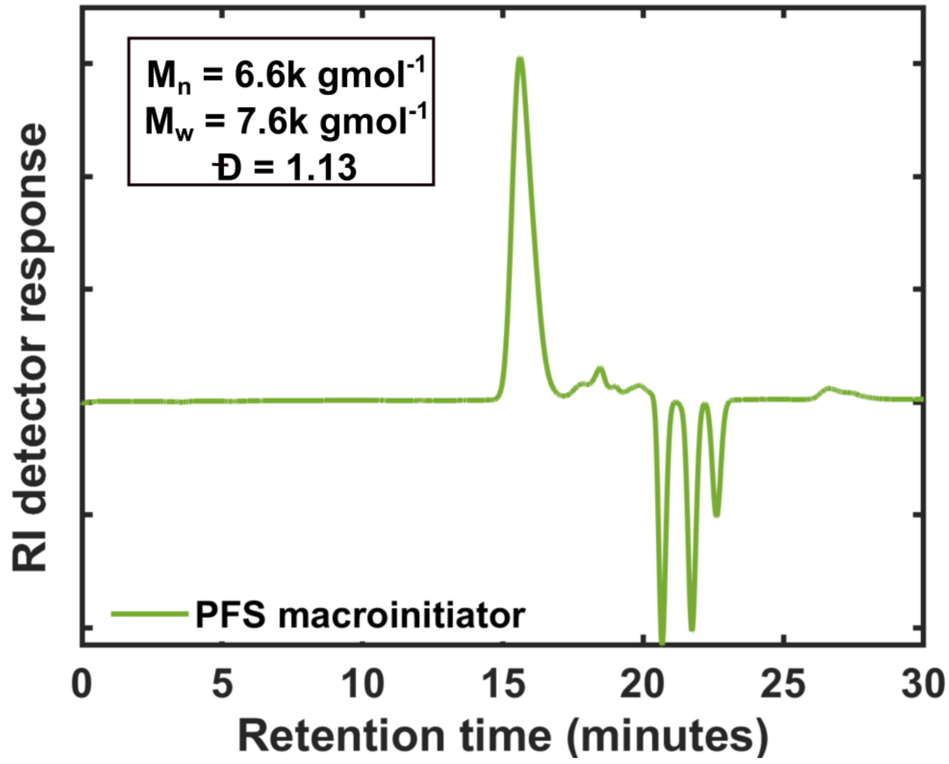

Figure S12. SEC for macroinitiator PFS **(10)**, performed in THF and calibrated with PS standards.

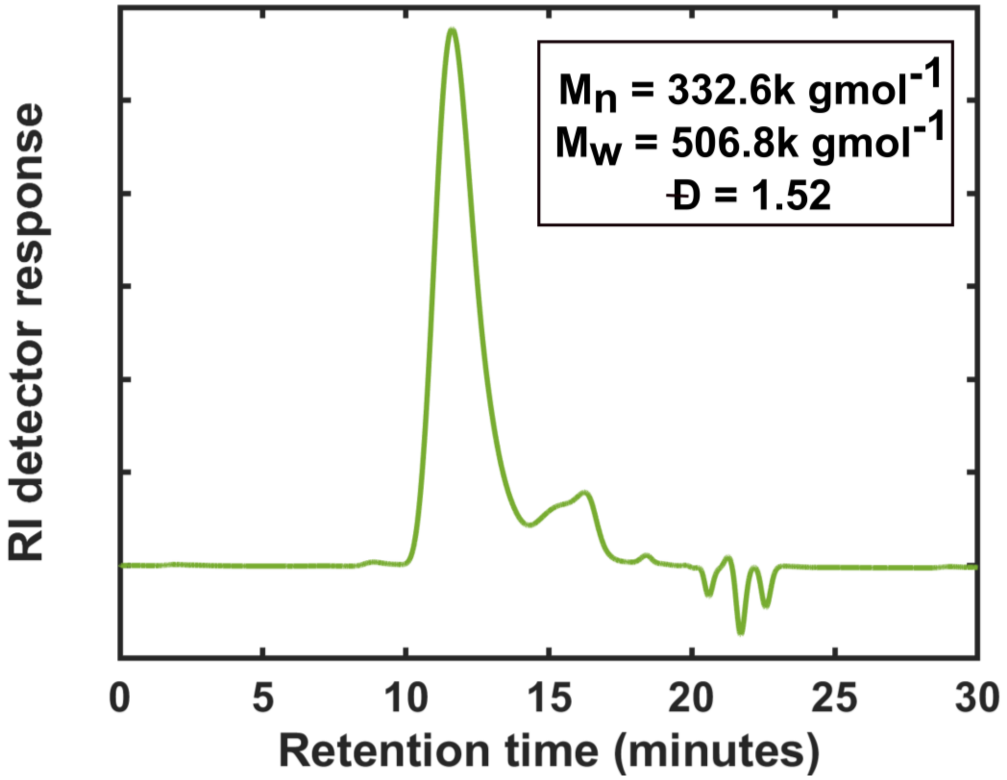

Figure S13. SEC for a high molecular weight macroinitiator PFS, performed in THF and calibrated with PS standards.

Step 2. Poly(hexylacrylate-*block*-2,3,4,5,6-pentfluorostyrene) (HFS) diblock polymer synthesis

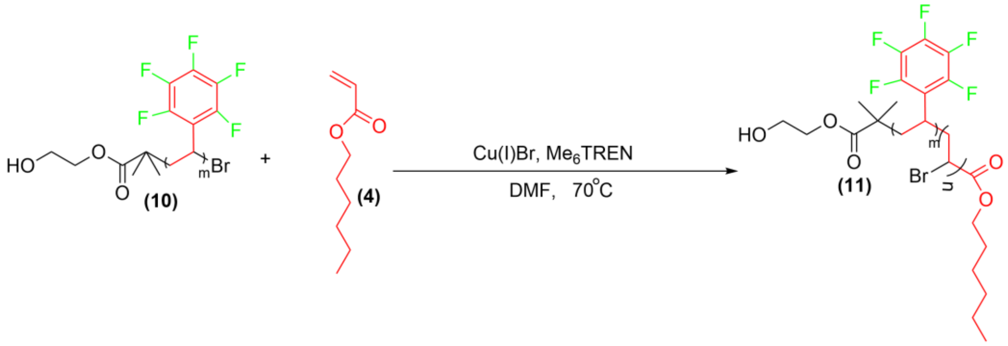

Scheme S6. Synthesis of HFS diblock copolymer **(11)**.

HFS diblock polymer **(11)** was synthesized via ATRP. Briefly stated, 5g (0.862 mmol) PFS macroinitiator **(10)** was dissolved in 10 mL DMF. This dissolved mixture was mixed with 18.4 mL (103 mmol) monomer hexyl acrylate **(4)** in a Schlenk flask. The flask was degassed via three cycles of FPT. Separately, a catalyst stock solution of 61.8 mg (0.431 mmol) Cu(I)Br and 120 μL (0.431 mmol) Me_6TREN ligand prepared in DMF was added into the flask via a syringe under flowing nitrogen gas. The reaction flask was placed in a pre-heated oil bath set at 70 ºC and continued for 48 hours. Upon completion, the reaction medium was diluted with DMF and precipitated in 5x excess chilled methanol. The obtained HFS diblock **(11)** was washed with chilled methanol twice and dried overnight at 40 ºC under vacuum. The product **(11)** was characterized by ^{1}HNMR (**Figure S14**). ^{1}HNMR peaks found at 1.80-2.05, and 2.26 ppm are

associated with $-CH_2$ and -CH(Br) on the backbone of hydrophobic polyhexylacrylate, assigned as f and g, respectively. Likewise, the signal for $-CH_2$ protons for side chain (peak h) was found at 4.01 ppm. NMR estimated M_n for HFS was 7.3k $gmol^{-1}$.

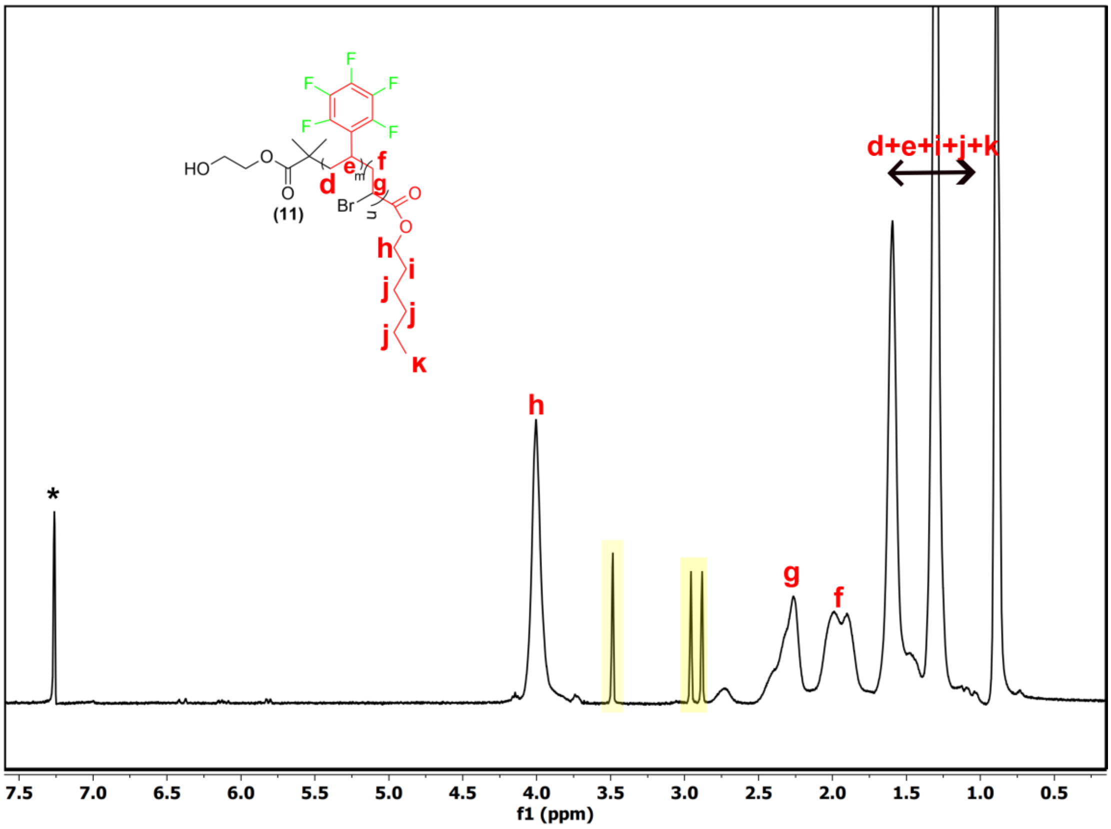

Figure S14. ^{1}HNMR of HFS diblock polymer (**11**), spectrum was obtained in $CDCl_3$. A strong peak at 7.26 ppm was found due to the residual $CHCl_3$ in the NMR solvent (marked by a *). Traces of precipitating solution methanol and reaction solvent DMF were observed at 3.49, 2.96 and 2.88 ppm respectively, highlighted in yellow color.

Step 3. Poly(hexylacrylate-*block*-petafluorostyrene-*block*-lactide) (HFSL) triblock polymer synthesis

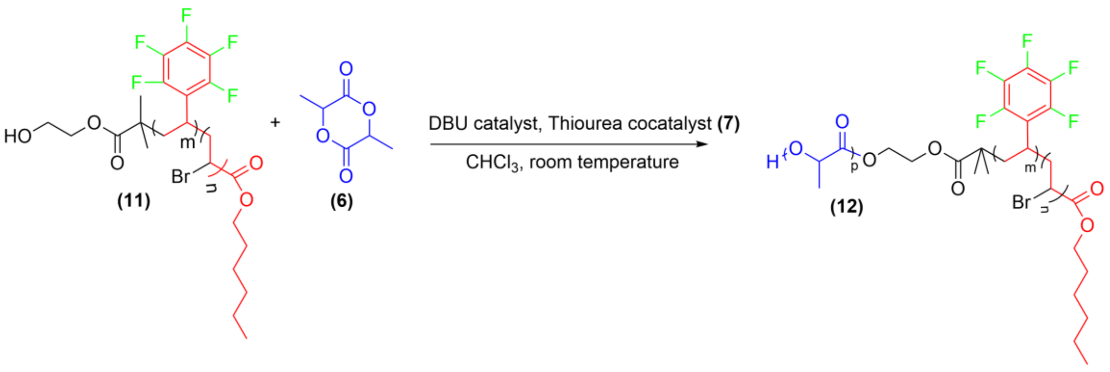

Scheme S7. Synthesis of HFSL triblock polymer **(12)**.

HFSL triblock polymer **(12)** was synthesized via organolactide ROP. Briefly stated, 5g (0.667 mmol) HFS diblock polymer **(11)** was dissolved in 10 mL anhydrous chloroform. This dissolved mixture was mixed with dried 4.8 g (33.3 mmol) lactide monomer **(6)** in a flame-dried Schlenk flask. Immediately after that 133.5 µL 1,8-diazabicyclo[5.4.0]undec-7-ene (DBU) (0.893 mmol) and 330 mg thiourea cocatalyst **(7)** (0.893 mmol) were added into the reaction flask. The reaction continued for 1 hour at room temperature under flowing constant nitrogen gas. The reaction was stopped by adding 109 mg benzoic acid (0.893 mmol). The crude mixture was precipitated in 5x excess chilled methanol. The obtained HFSL triblock polymer **(12)** was washed with chilled methanol twice and dried overnight at 40 ºC under vacuum. The product **(12)** was characterized by ^{1}HNMR, ^{19}FNMR and SEC. In the ^{1}HNMR spectrum (**Figure S15**), the chemical shifts (δ) found at 1.80-2.05, and 2.26 ppm have been assigned to the $-CH_2$ and -C(H)Br protons in the polyhexylacrylate, labeled as c and b, respectively. Whereas signal for $-CH_2$ protons (peak d) for polyhexylacrylate was found at 4.01 ppm. A broad signal observed at 5.10-5.24 ppm is linked to the -CH protons (peak a) for polylactide. The sum of the aliphatic $-CH-CH_2$ protons for pentafluorostyrene (peaks h and i) was noted in the range of 1.70-3.0 ppm. Since all aromatic protons have been substituted with fluorine atoms in pentafluorostyrene, consequently, no signals responsible for aromatic protons are observed in the vicinity of approximately 7 ppm. Additionally, the protons signal from initiator (peak r and r') was observed at 3.73-3.78 ppm. The NMR estimated M_n for a representative HFSL triblock polymer was 8.4k $gmol^{-1}$. HFSL was further characterized by ^{19}FNMR (**Figure S16**), which shows 3 distinct peaks for 3 different fluorine environments at 143.1, -154.1 and -161.1 ppm, that suggests successful synthesis.[6] SEC profile for macroinitiator PFS **(10)**, FSH diblock polymer **(11)** and HFSL1 **(12)** is shown in **Figure S17**. The discrepancy of M_n measured by HNMR ($M_{n,NMR}$ = 8.4k $gmol^{-1}$) and SEC ($M_{n,SEC}$ = 27.5k $gmol^{-1}$) was attributed to the difference in hydrodynamic volume of fluorinated styrene-based block polymer and the SEC calibrant non-fluorinated styrene.[7] A series of HFSL polymers with the M_n range of 8k to 30k $gmol^{-1}$ was synthesized.

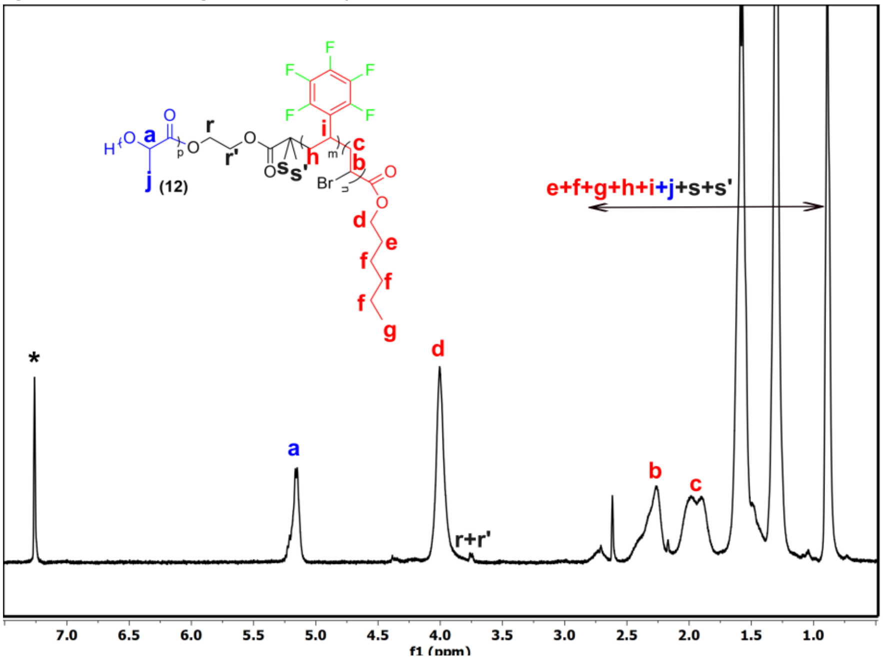

Figure S15. ^{1}HNMR of HFSL triblock polymer (**12)**, spectrum was obtained in $CDCl_3$. A strong peak at 7.26 ppm was found due to the residual $CHCl_3$ in the NMR solvent (marked by *).

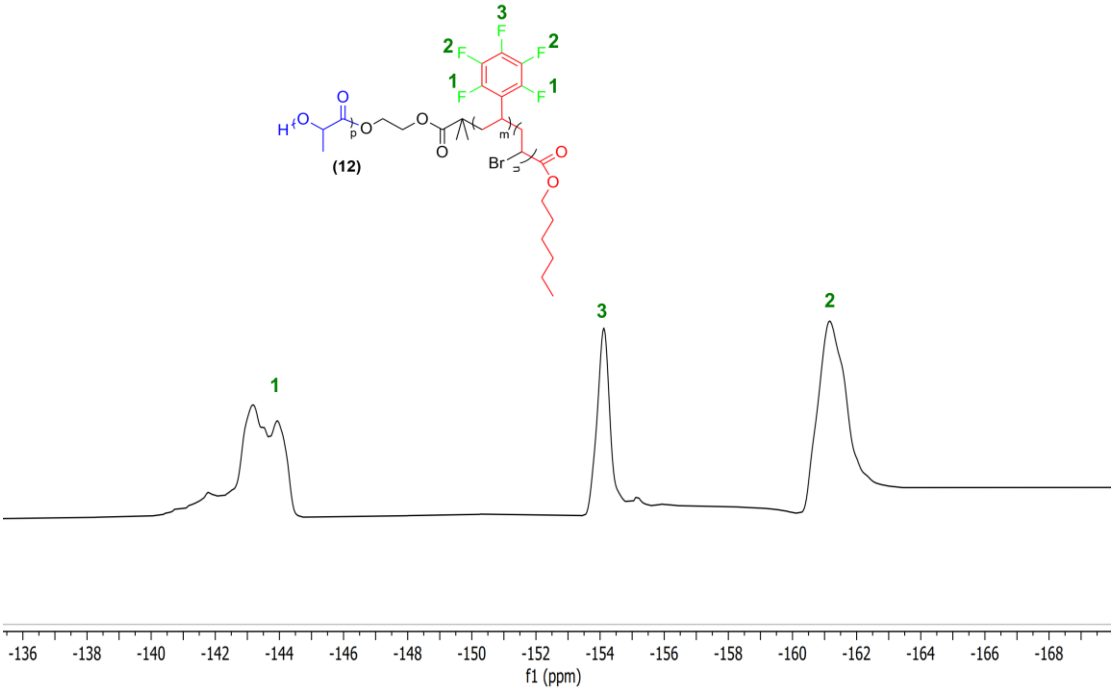

Figure S16. ^{19}FNMR spectrum of HFSL triblock polymer.

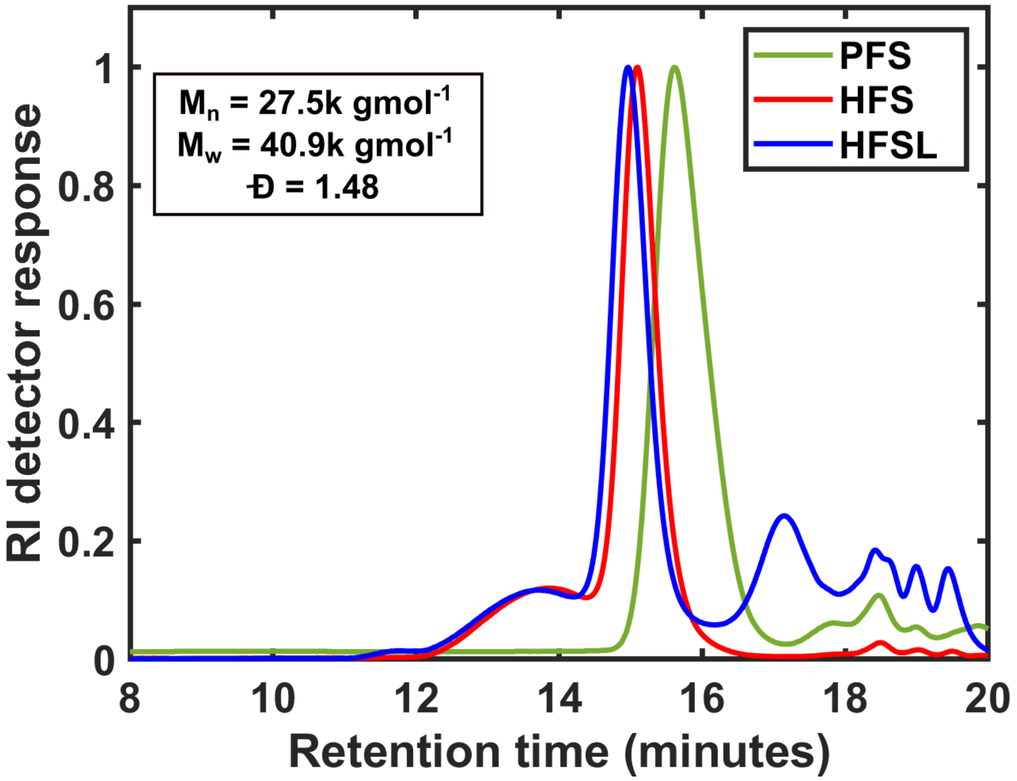

Figure S17. SEC profile for macroinitiator PFS **(10)**, HFS diblock polymer **(11)** and triblock polymer candidate HFSL **(12)**. A peak of a higher molecular weight compared to the main peak was noted, that causes the increase in Đ. The bimodal SEC distribution for HFS diblock polymer suggests termination reaction via coupling during ATRP reaction. The discrepancy of M_n measured by HNMR ($M_{n,NMR}$ = 8.4k gmol^{-1}) and SEC ($M_{n,SEC}$ = 27.5k gmol^{-1}) is attributed

to the difference of hydrodynamic volume of fluorinated styrene-based block polymer and the SEC calibrant non-fluorinated styrene.[7]

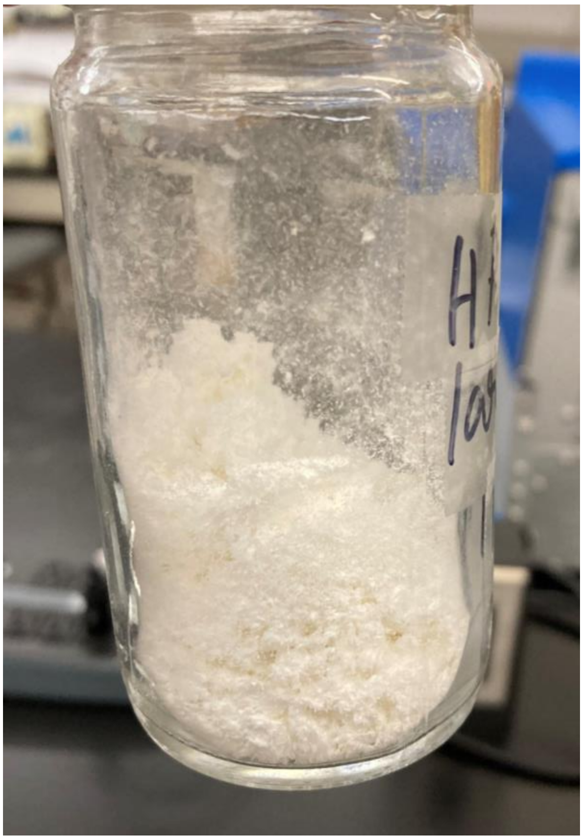

Figure S18. A photograph of the sorbent **Candidate 2** HFSL5, that is used in the PFOA adsorption process.

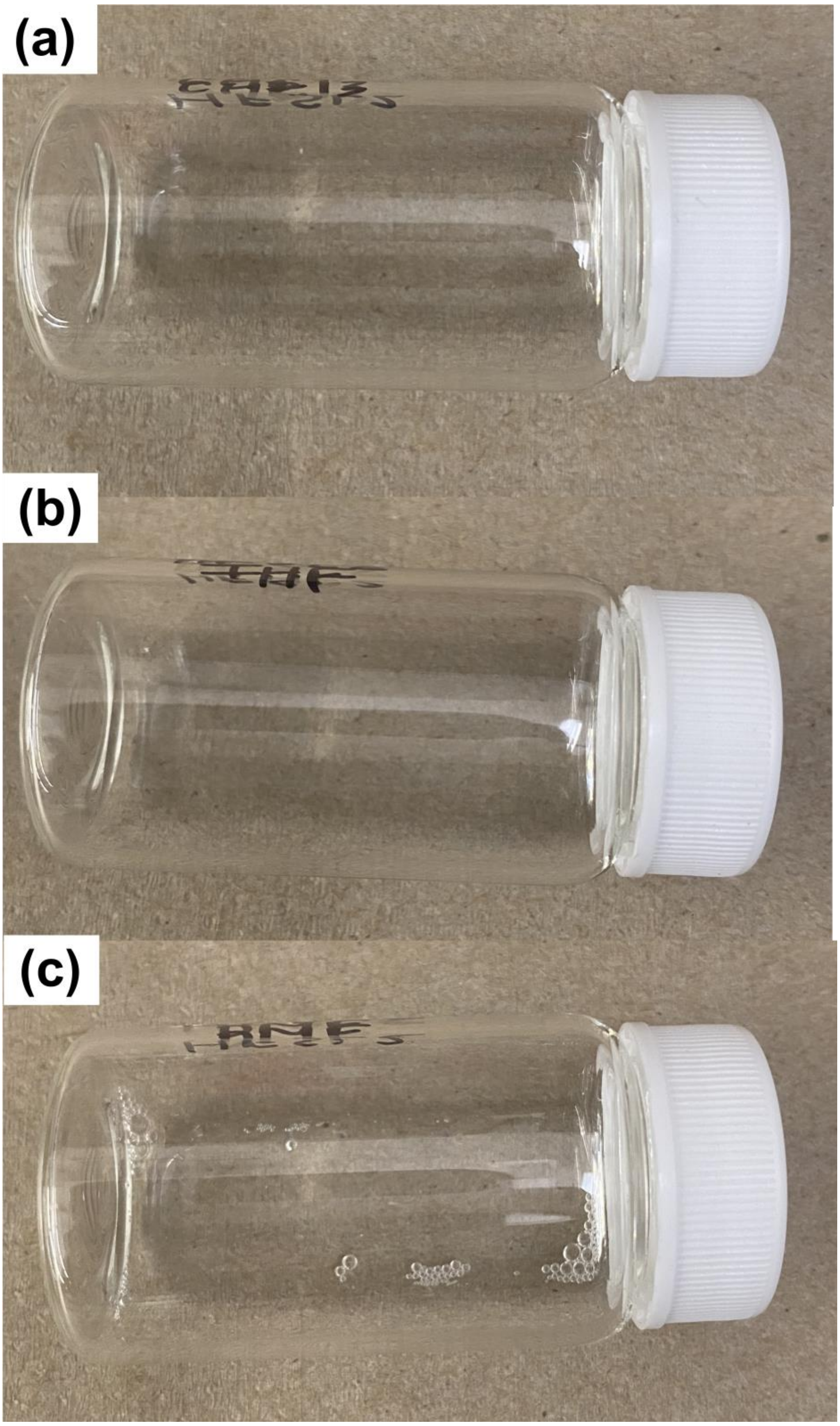

Figure S19. A photograph of 1 wt/vol% solution of **Candidate 2** HFSL5 in chloroform (a), THF (b) and DMF (c). Photographs were taken after 2 minutes vortex of polymer solutions at room temperature. All solutions demonstrate a clear appearance and confirm excellent solubility of HFSL5 in common organic solvents.

Sorbent Candidate 3 HFBuMaSL

Polymer Candidate 3 poly(hexafluorobutylmethacrylate)-*block*-(styrene)-*block* (lactide) (HFBuMaSL).

Step 1. Polystyrene (PS) macroinitiator synthesis via ARGET ATRP

Polystyrene **(3)** macroinitiator was synthesized via ARGET ATRP, described in **Scheme 1**. The number average molas mass (M_n) was estimated as 12.4k gmol^{-1} by ^{1}HNMR (**Figure S1**) and as 13.6k gmol^{-1} by SEC (**Figure S2**).

Step 2. Poly(2,2,3,4,4,4-hexafluorobutylmethacrylate-*block*-styrene) (HFBuMaS) diblock polymer synthesis

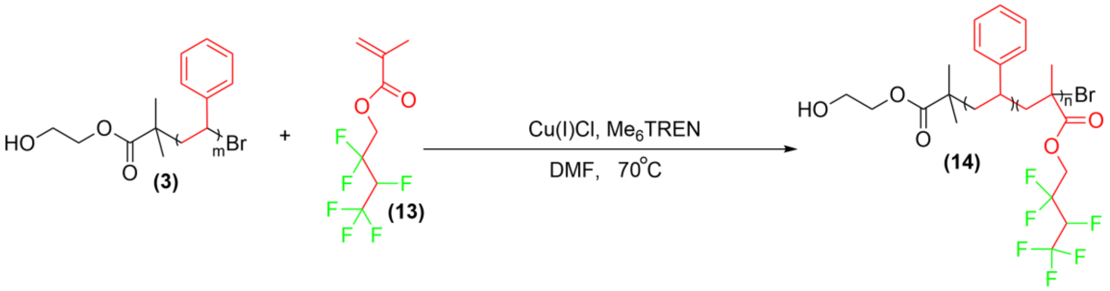

Scheme S8. Synthesis of HFBuMaS diblock polymer **(14)**.

HFBuMaS diblock polymer **(14)** was synthesized via ATRP. Briefly stated, 5g (0.403 mmol) PS macroinitiator **(3)** was dissolved in 20 mL DMF. This dissolved solution was mixed with 9 mL (48.3 mmol) monomer 2,2,3,4,4,4-hexafluorobutylmethacrylate **(13)** in a Schlenk flask. The flask was degassed via three cycles of FPT. Separately, a catalyst stock solution of 20 mg (0.201 mmol) Cu(I) Cl and 54 µL (0.201 mmol) Me_6TREN ligand prepared DMF was added into the flask via a syringe under flowing nitrogen gas. The reaction flask was placed in a pre-heated oil bath set at 70 °C and continued for 46 hours. Upon completion, the reaction medium was diluted with DMF and precipitated in 5x excess chilled methanol. The obtained HFBuMaS diblock **(14)** was washed with chilled methanol twice and dried overnight at 40 °C under vacuum. The product **(14)** was characterized by ^{1}HNMR (**Figure S20**) and SEC (**Figure S21**). Aromatic protons signal for polystyrene (labeled as a) was observed at ~7 ppm in ^{1}HNMR (**Figure S20**). Peaks responsible for -CH_3 (b) and -CH_2 (c) from HFBuMa were noted at 0.7-1.2 ppm and 1.8-2.1 ppm, which overlapped with peaks of polystyrene backbone (labeled as f, g). In addition, peaks appear at 4.1-4.4 ppm and 4.7-5.1 ppm correspond to the protons of -OCH_2 (d) and -$CHFCF_3$ (e).[7] The splitting of peak e is attributed to the coupling of proton with nuclei of fluorine atoms. The M_n for the HFBuMaS diblock polymer estimated from the NMR was 19.3k gmol^{-1}, where the M_n for HFBuMa is calculated as 6.9k gmol^{-1}. Clear shift of the monomodal SEC trace (**Figure S21**) for HFBuMaS towards higher molecular weight (lower retention time) suggests the successful synthesis of diblock polymer. The SEC estimated M_n value of 14.7k gmol^{-1} with a narrow dispersity (Đ) of 1.15 suggests a controlled polymerization.

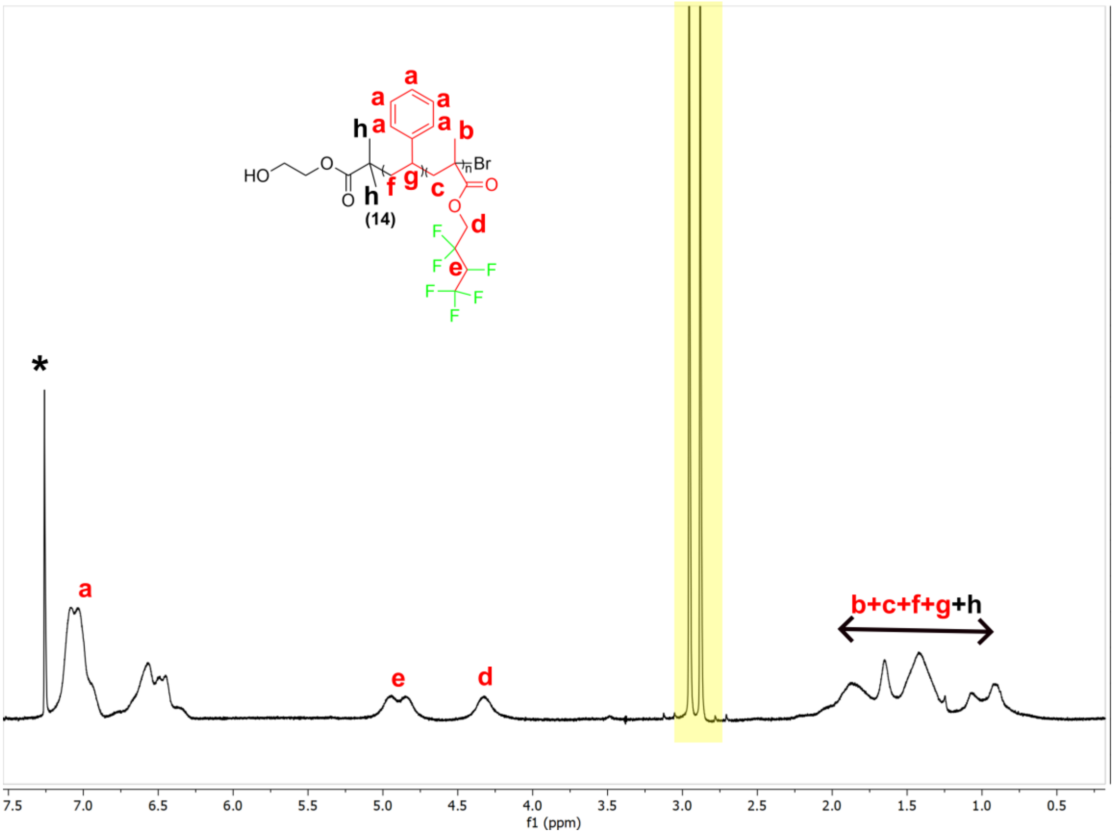

Figure S20. ^{1}HNMR of HFBuMaS diblock polymer (**14)**, spectrum was obtained in $CDCl_3$. A strong peak at 7.26 ppm was found due to the residual $CHCl_3$ in the NMR solvent. Strong peaks obtained at 2.96 and 2.88 ppm are due to DMF, highlighted in yellow.

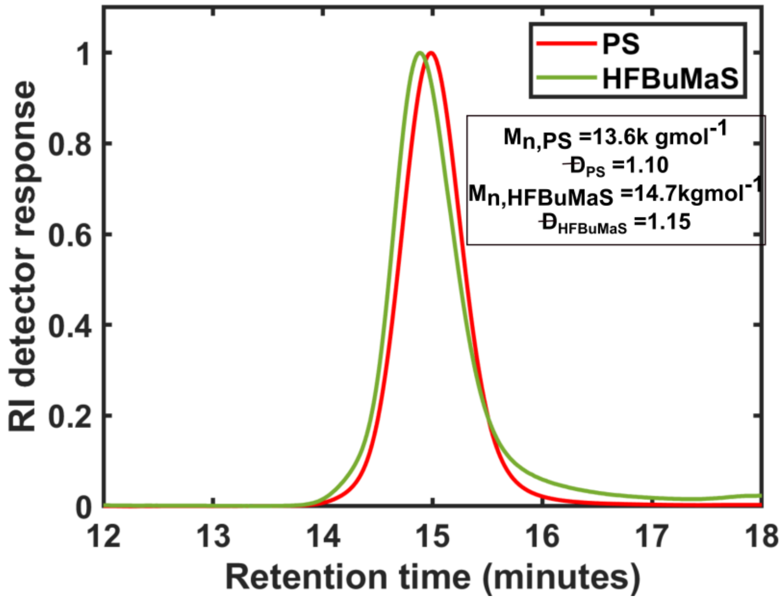

Figure S21. SEC profile for diblock polymer HFBuMaS **(14)** along the macroinitiator PS **(3)**.

Step 3. Poly(hexafluorobutylmethacrylate-*block*-styrene-*block*-lactide) (HFBuMaSL) triblock polymer synthesis

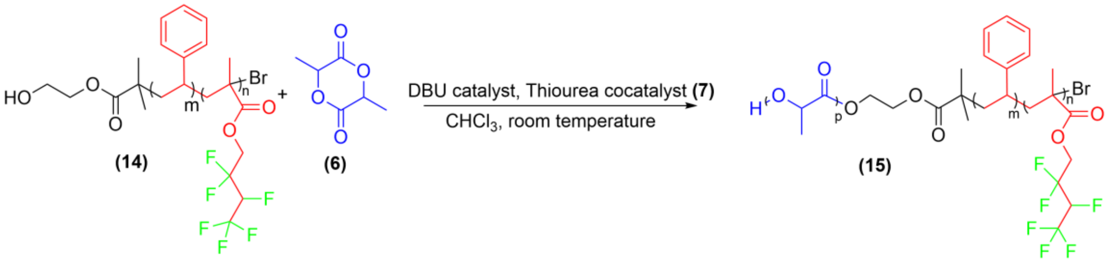

Scheme S9. Synthesis of HFBuMaSL triblock polymer **(15)**.

HFBuMaSL triblock polymer **(15)** was synthesized via lactide ROP. Briefly stated, 5g (0.26 mmol) HFBuMaS diblock polymer **(14)** was dissolved in 10 mL anhydrous chloroform. This dissolved mixture was mixed with dried 3.7 g (25.9 mmol) lactide monomer **(6)** in a flame-dried Schlenk flask. Immediately after that 52 µL 1,8-diazabicyclo[5.4.0]undec-7-ene (DBU) (0.347 mmol) and 128 mg thiourea cocatalyst **(7)** (0.347 mmol) were added into the reaction flask immediately after that. The reaction continued for 1 hour at room temperature under flowing constant nitrogen gas. The reaction was stopped by adding 42 mg benzoic acid (0.347 mmol). The crude mixture was precipitated in 5x excess chilled methanol. The obtained HFBuMaSL triblock polymer **(15)** was washed with chilled methanol twice and dried overnight at 40 °C under vacuum. The product was characterized by ^{1}HNMR (**Figure S22**), ^{19}F NMR (**Figure S23**), and SEC (**Figure S24**). In ^{1}HNMR, in addition to the peaks from polystyrene and polyhexafluorobutylmethacrylate, a broad signal observed at 5.10-5.24 ppm is linked to the -CH protons (peak i) for polylactide. The M_n for the HFBuMaSL triblock polymer estimated was 25.1k gmol^{-1}. ^{19}FNMR shows 3 distinct peaks for 3 different F environments including terminal CF_3 group (labeled as 3) in the low-field region at -75 ppm. Middle -CHF peak (labeled as 2) appears at the high field region of -212.6 ppm. Lastly, the CF_2 group (labeled as 1) adjacent to the -OCH_2 group splits into two peaks and appears at -114 and -120 ppm. ^{19}FNMR of HFBuMaSL is consistent to the prior litertaure,[8] that confirms the successful polymerization of fluorinated block. SEC estimated molecular weights for HFBuMaSL are unreliable, as polymer aggregate of high molecular weight was found in lower retention time at SEC curve (**Figure S24**). This phenomenon was reported earlier where fluorinated block polymers form micelle structures in SEC solvent THF.[9] A series of HFBuMaSL polymers with the M_n range of 20k to 40k gmol^{-1} is synthesized.

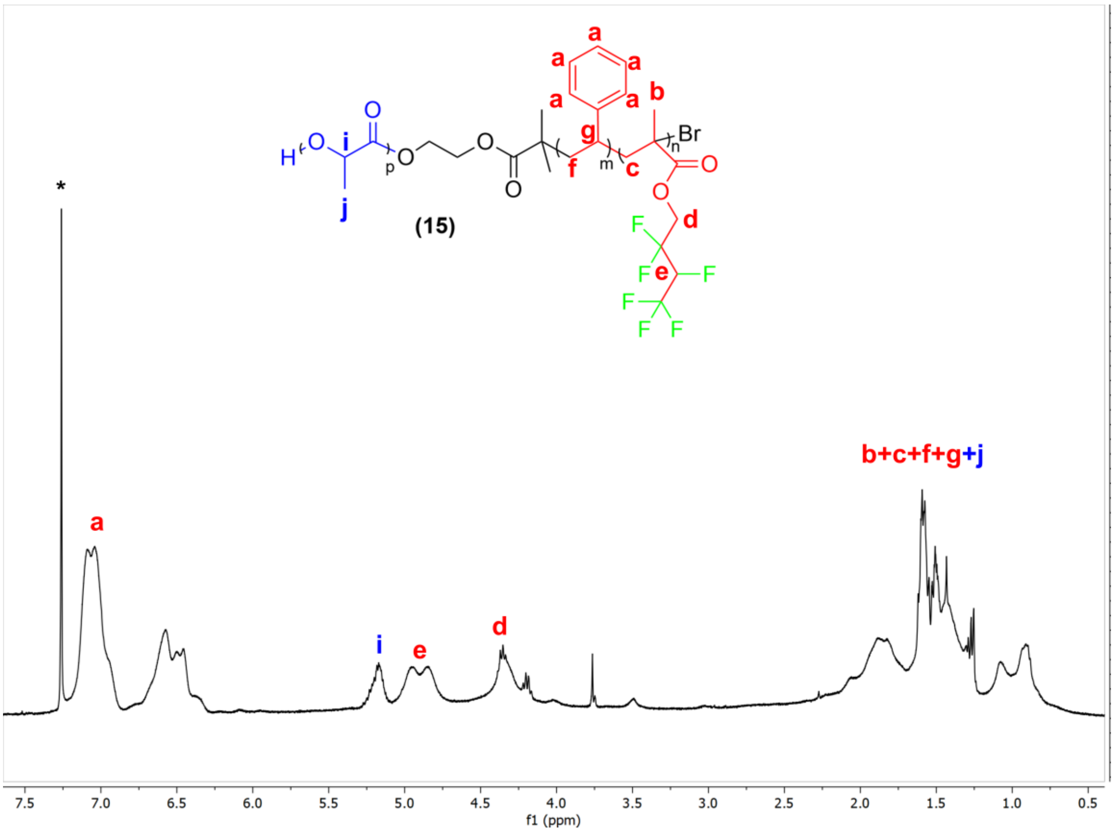

Figure S22. ^{1}HNMR of HFBuMaSL triblock polymer (**15)**, spectrum was obtained in $CDCl_3$. A strong peak at 7.26 ppm was found due to the residual $CHCl_3$ in the NMR solvent.

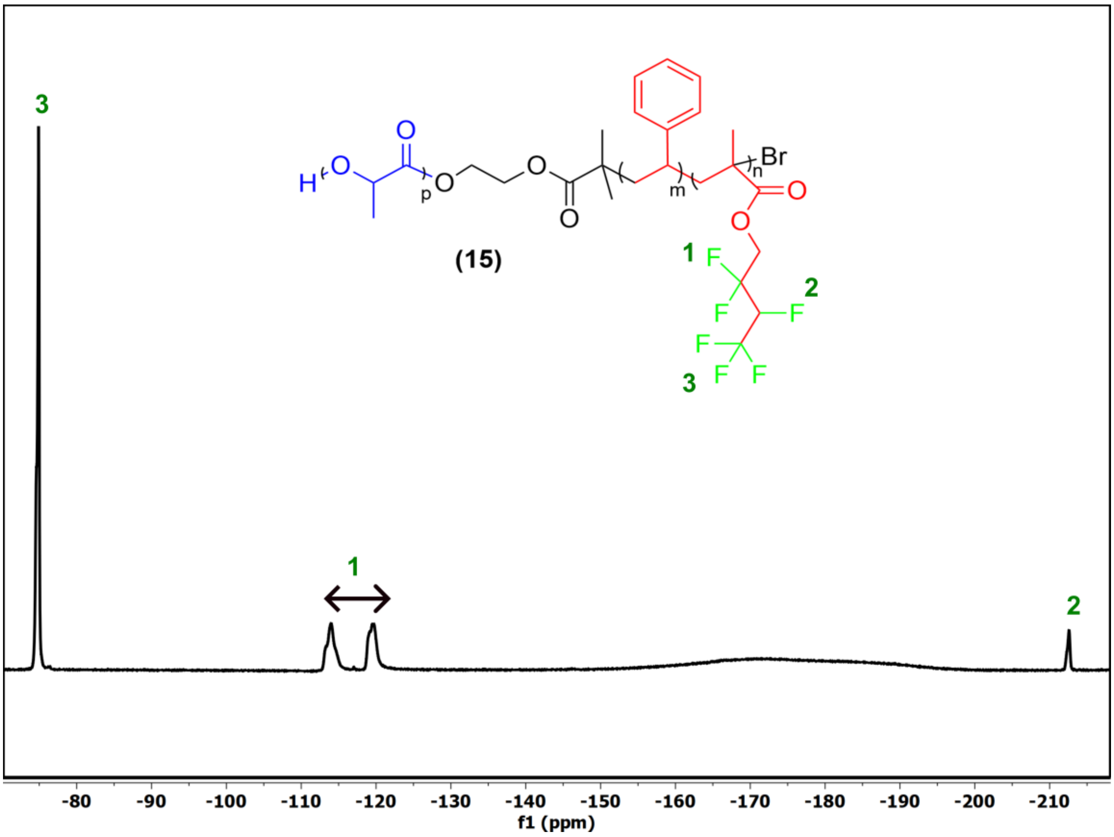

Figure S23. ^{19}FNMR spectrum of **Candidate 3** HFBuMaSL triblock polymer.

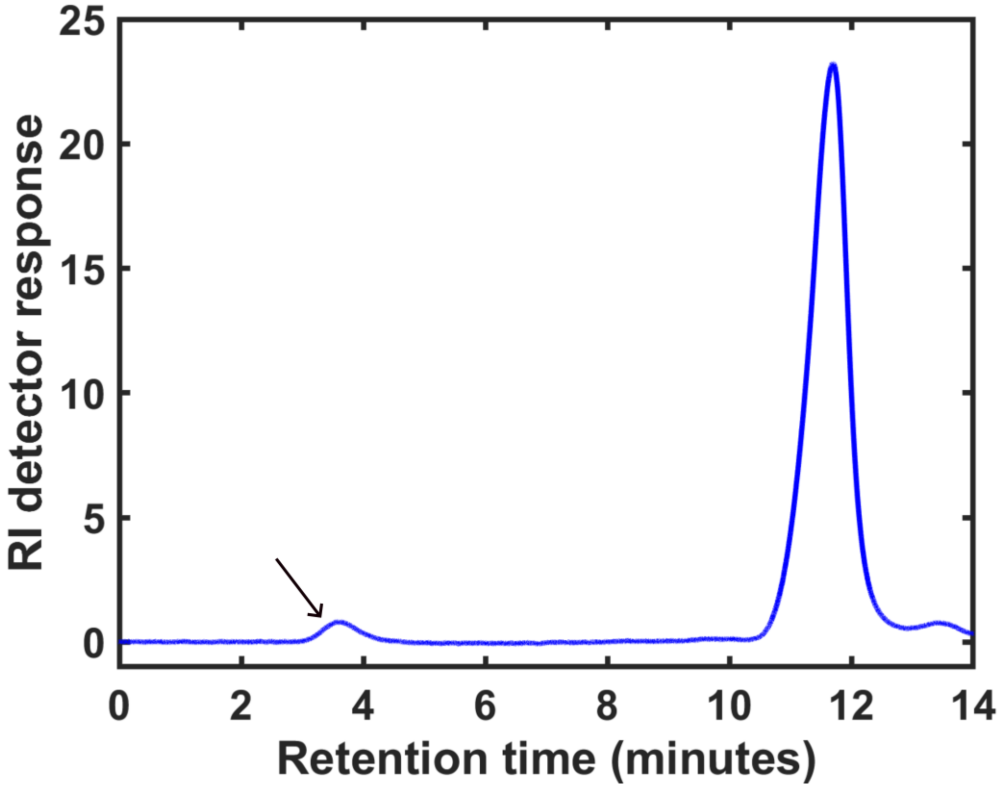

Figure S24. SEC profile for HFBuMaSL (**15**). The peak found at the lower elution time (~4 minutes) possibly originates due to the formation of aggregate or micelles of triblock polymer in the SEC solvent THF (shown by arrow).[9] Thus, the SEC estimated molecular weights for HFBuMaSL is unreliable.

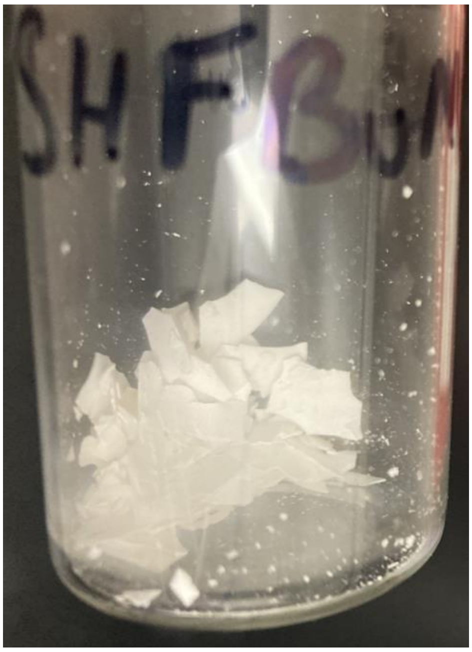

Figure S25. A photograph of the sorbent **Candidate 3** HFBuMaSL5.

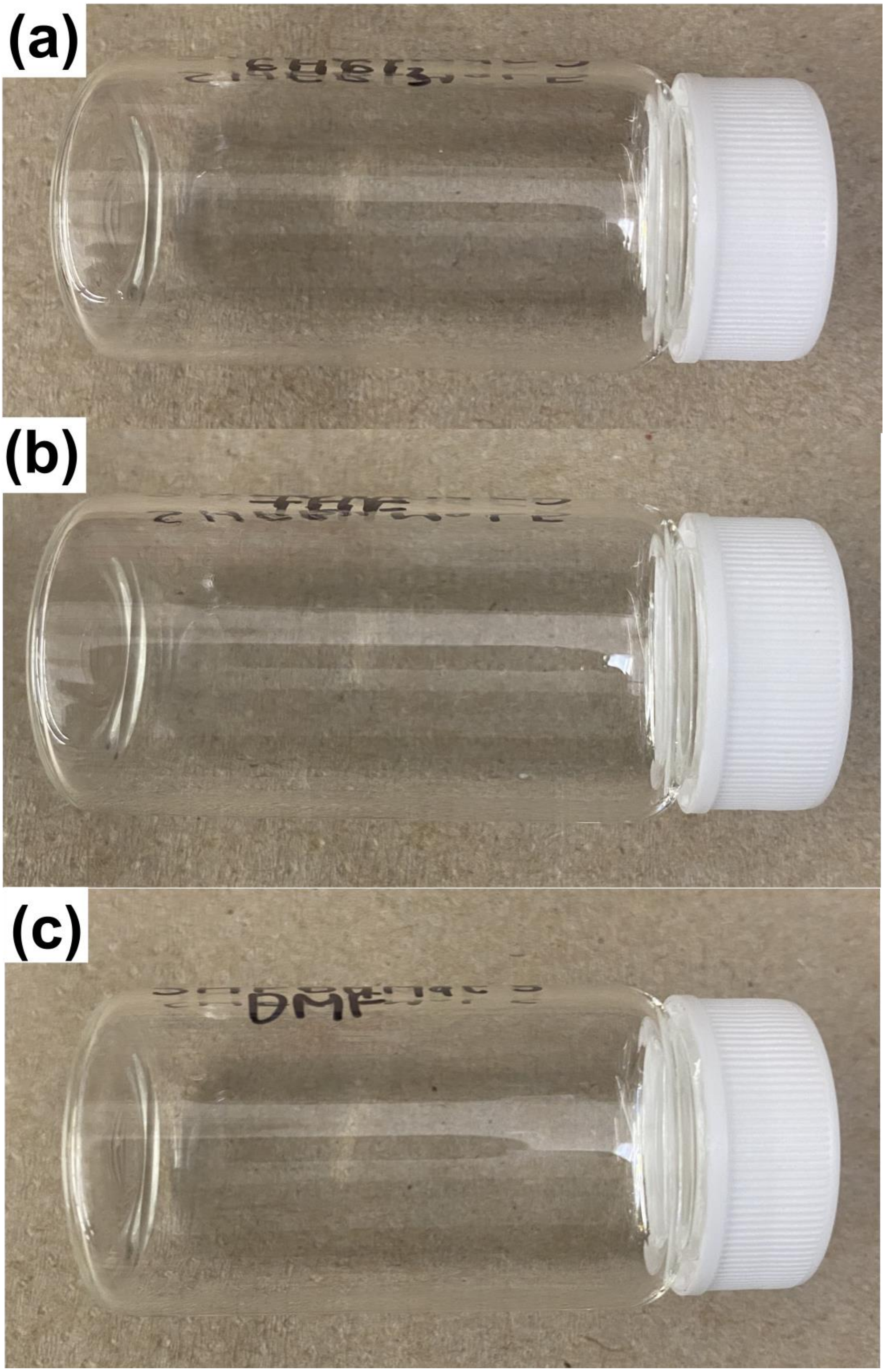

Figure S26. A photograph of 1 wt/vol% solution of **Candidate 3** HFBuMaSL5 in chloroform (a), THF (b) and DMF (c). Photographs were taken after 2 minutes vortex of polymer solutions at room temperature. All solutions demonstrate clear appearances and confirm excellent solubility of HFBuMaSL5 in common organic solvents.

Sorbent Candidate 4

Polymer Candidate 4 poly(methyl-2-fluoroacrylate)-*block*-(styrene)-*block* (lactide) (MeFSL).

Step 1. Polystyrene (PS) macroinitiator synthesis via ARGET ATRP

Polystyrene **(3)** macroinitiator was synthesized via ARGET ATRP, described in **Scheme 1**. The number average molas mass (M_n) is estimated as 12.4k gmol^{-1} by ^{1}HNMR (**Figure S1**) and as 13.6k gmol^{-1} by SEC (**Figure S2**).

Step 2. Poly(methyl-2-fluoroacrylate-*block*-styrene) (MeFS) diblock polymer synthesis

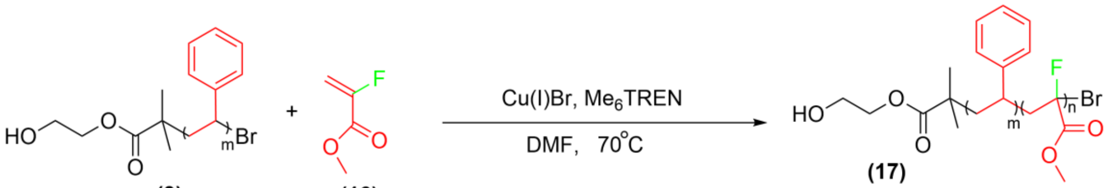

(3) **(16)** **(17)**

Scheme S10. Synthesis of MeFS diblock copolymer **(17)**.

MeFS diblock polymer **(17)** was synthesized via ATRP. Briefly stated, 5g (0.403 mmol) PS macroinitiator **(3)** was dissolved in 20 mL DMF. This dissolved solution was mixed with 9.4 mL (101 mmol) monomer methyl-2-fluoroacrylate **(16)** in a Schlenk flask. The flask was degassed via three cycles of FPT. Separately, a catalyst stock solution of 29 mg (0.201 mmol) Cu(I)Br and 54 µL (0.201 mmol) Me_6TREN ligand prepared in DMF was added into the flask via a syringe under flowing nitrogen gas. The reaction flask was placed in a pre-heated oil bath set at 70 ºC and continued for 46 hours. Upon completion, the reaction medium was diluted with DMF and precipitated in 5x excess chilled methanol. The obtained MeFS diblock **(17)** was washed with chilled methanol twice and dried overnight at 40 ºC under vacuum. The product **(17)** was characterized by ^{1}HNMR (**Figure S27**) and SEC (**Figure S28**). In ^{1}HNMR (**Figure S27**), aromatic protons signal for polystyrene (labeled as a) was observed at 6.5-7 ppm. Peaks responsible for $-CH_3$ (b) from methyl-2-fluoroacrylate appear at 3.75 ppm. Likewise $-CH_2$ (c) from methyl-2-fluoroacrylate was noted at 2.24-2.62 ppm. Moreover, peaks appear at 1.20-2.10 ppm correspond to $-CH_2$ (d), and -CH (h) from polystyrene and protons (e, e') from initiator. Additionally, the protons signal from initiator (peak g and f) was observed at 3.5 ppm. The M_n for the MeFS diblock polymer estimated from this NMR spectrum was 22.6k gmol^{-1}. Clear shift of the monomodal SEC trace (**Figure S28**) for MeFS towards higher molecular weight (lower retention time) suggests the successful synthesis of diblock polymer. The SEC estimated M_n value is 25.7k gmol^{-1} with a narrow dispersity (Đ) of 1.21 suggests a controlled polymerization.

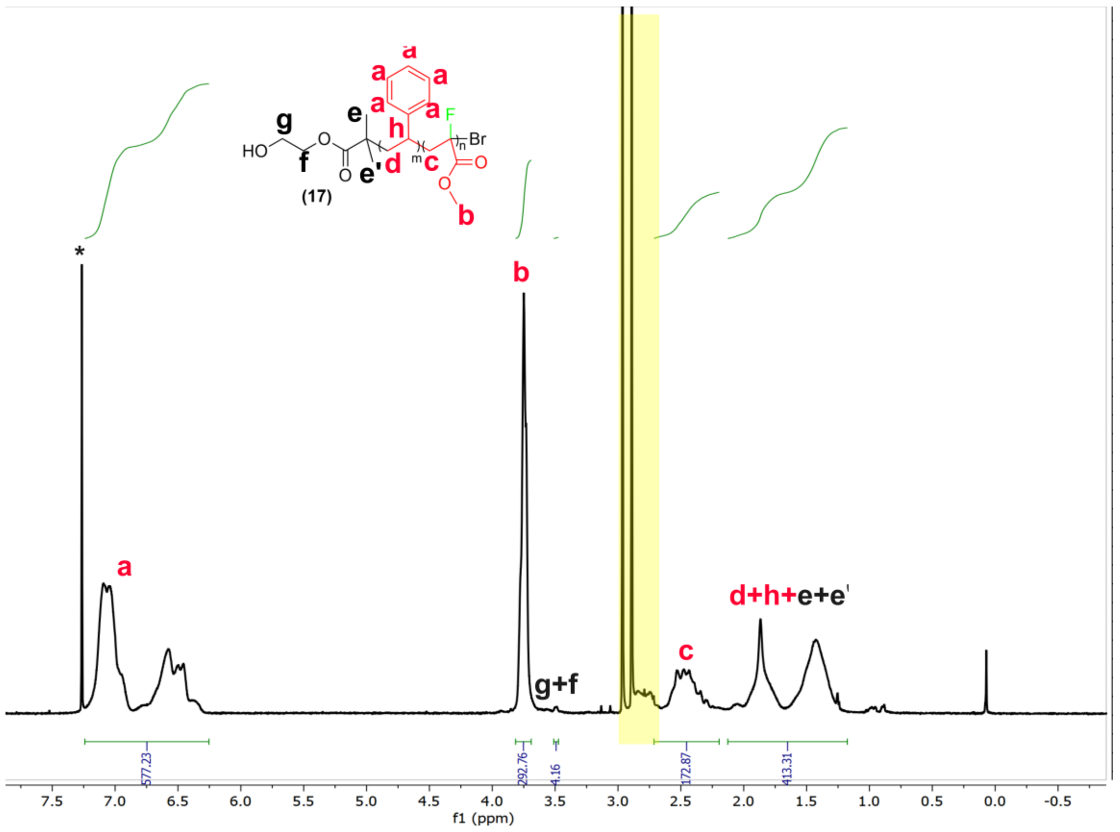

Figure S27. ^{1}HNMR of MeFS diblock polymer (**17**), spectrum was obtained in $CDCl_3$. strong peak at 7.26 ppm was found due to the residual $CHCl_3$ in the NMR solvent. Strong peaks obtained at 2.96 and 2.88 ppm are due to DMF, highlighted in yellow.

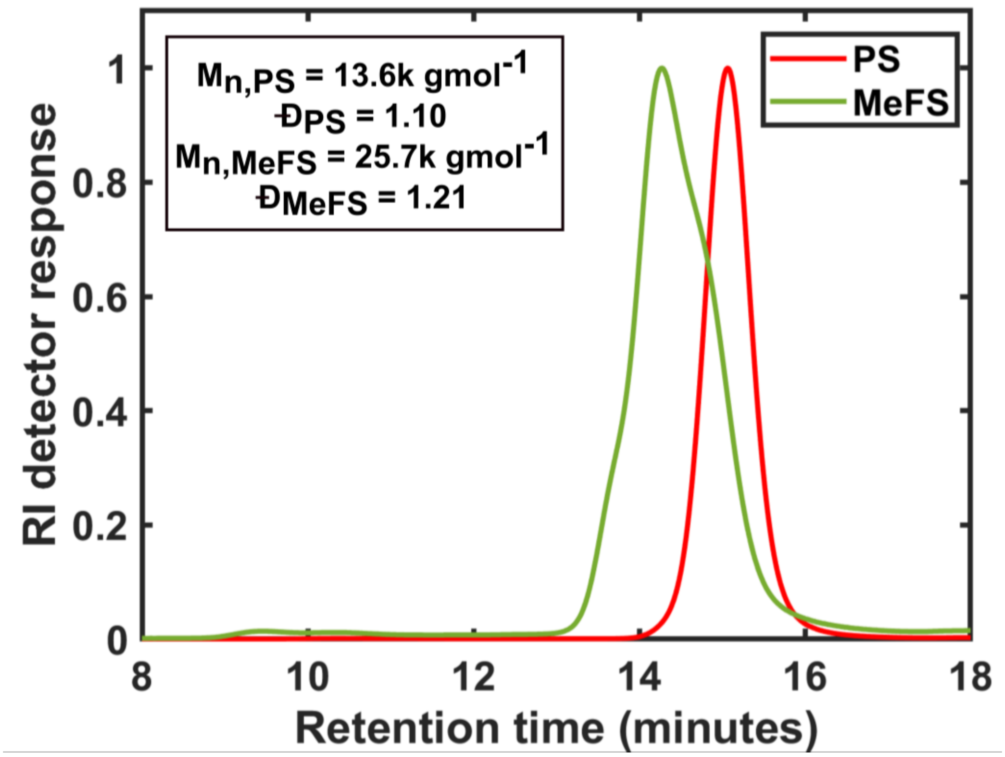

Figure S28. SEC profile for diblock polymer MeFS **(17)** along the macroinitiator PS **(3)**.

Step 3. Poly(methyl-2-fluoroacrylate-*block*-styrene-*block*-lactide) (MeFSL) triblock polymer synthesis

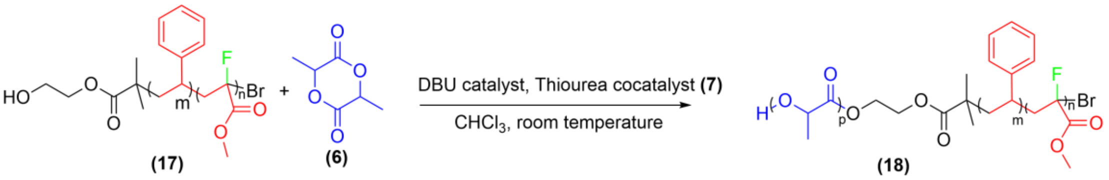

(17) **(6)** **(18)**

Scheme S11. Synthesis of MeFSL triblock polymer **(18)**.

MeFSL triblock polymer **(18)** was synthesized via ROP. Briefly stated, 5g (0.221 mmol) MeFS diblock polymer **(17)** was dissolved in 10 mL anhydrous chloroform. This solution was mixed with dried 3.2 g (22.1 mmol) lactide monomer **(6)** in a flame-dried Schlenk flask. Immediately after that 44 µL 1,8-diazabicyclo[5.4.0]undec-7-ene (DBU) (0.296 mmol) and 110 mg thiourea cocatalyst **(7)** (0.296 mmol) were added into the reaction flask immediately after that. The reaction continued for 1 hour at room temperature under flowing constant nitrogen gas. The reaction was stopped by adding 36 mg benzoic acid (0.296 mmol). The crude mixture was precipitated in 5x excess chilled methanol. The obtained MeFSL triblock polymer **(18)** was washed with chilled ethanol twice and dried overnight at 40 ºC under vacuum. The product was characterized by ^{1}HNMR (**Figure S29**), ^{19}FNMR (**Figure S30**) and SEC (**Figure S31**). In ^{1}HNMR, along the other peaks a broad signal observed at 5.10-5.24 ppm is linked to the -CH protons (peak f) for poly(lactide). The M_n for the MeFSL triblock polymer estimated from the NMR spectrum was 29.1k gmol^{-1}. A series of MeFSL polymers with the M_n range of 25k to 40k gmol^{-1} is synthesized. ^{19}FNMR (**Figure S30**) of MeFSL shows broad signals in the region of -160.5 to -172 ppm, which is typically observed for poly(α-fluoroacrylate).[10,11] SEC estimated molecular weights for MeFSL is unreliable, as polymer aggregate of high molecular weight was found in lower retention time at SEC curve (**Figure S31**). This phenomenon was reported earlier where fluorinated block polymers form micelle structures in SEC solvent THF.[9] Additionally, MeFSL was not fully soluble in SEC eluent THF, please see **Figure S32**.

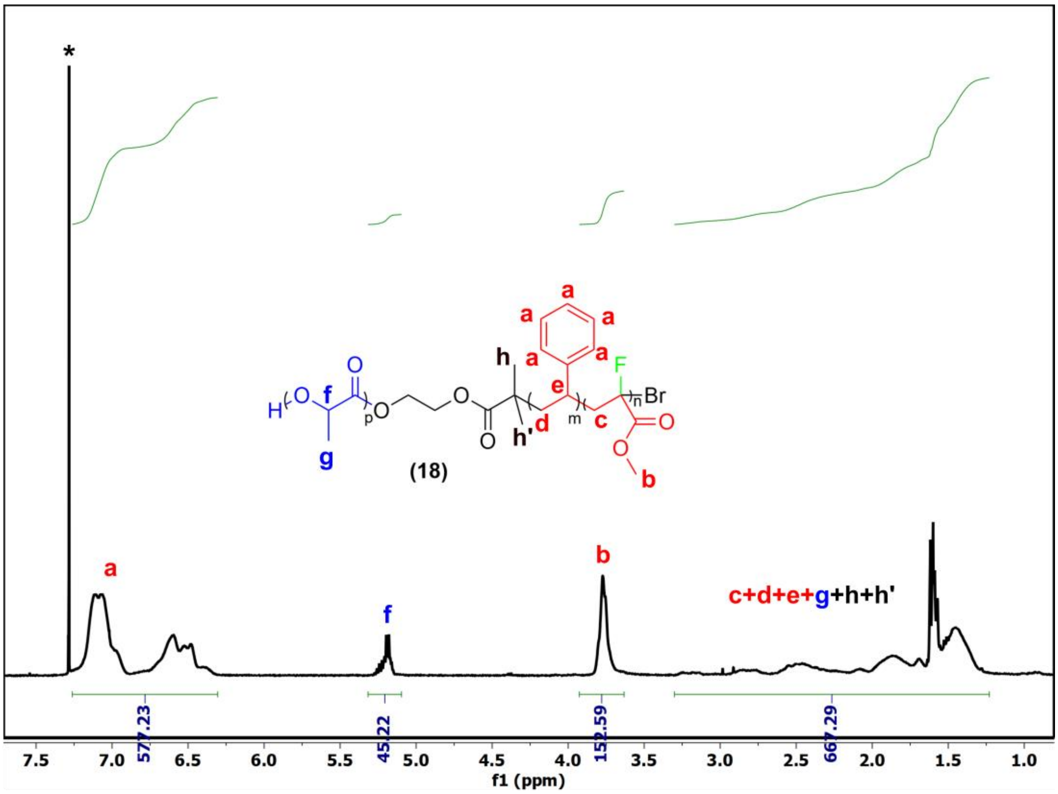

Figure S29. ^{1}HNMR of MeFSL triblock polymer (**18)**, spectrum was obtained in $CDCl_3$. A strong peak at 7.26 ppm was found due to the residual $CHCl_3$ in the NMR solvent.

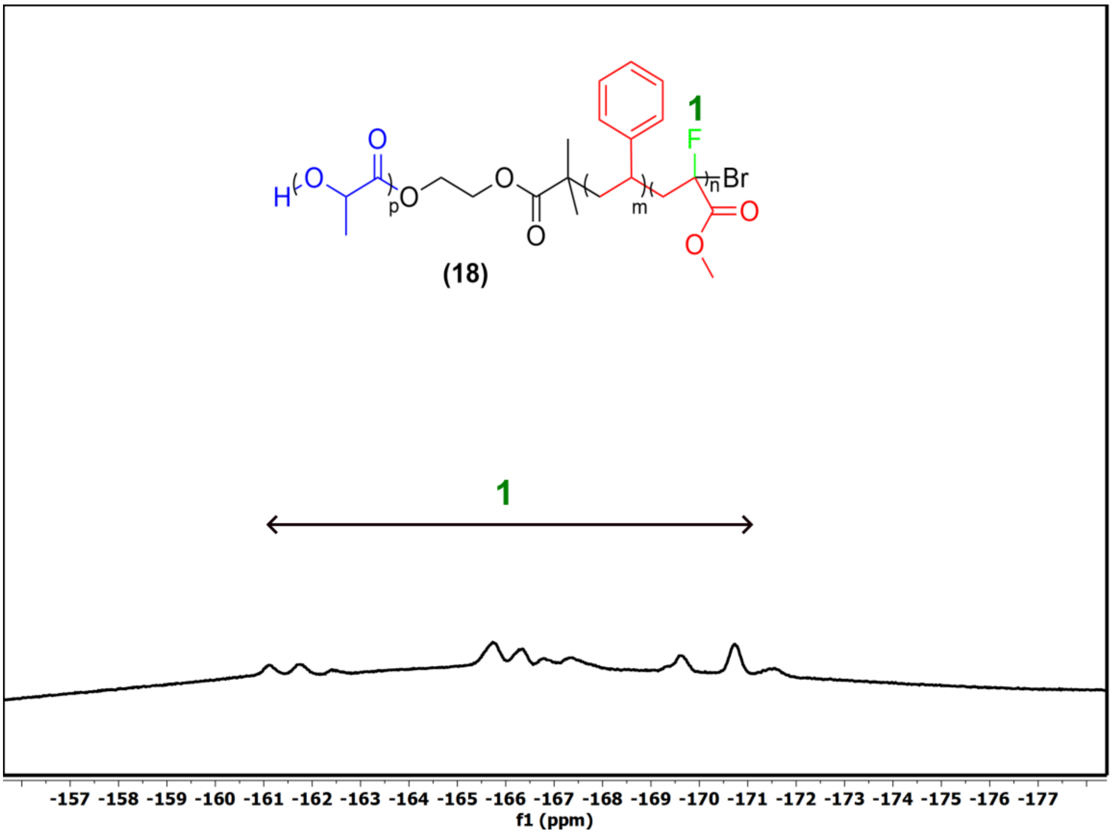

Figure S30. ^{19}FNMR spectrum of **Candidate 4** MeFSL triblock polymer. A couple of different peaks are found in the region of -160.5 to -172 ppm, which is attributed to the strong sensitivity of the F atoms in MeFSL to the stereochemistry.[10,11]

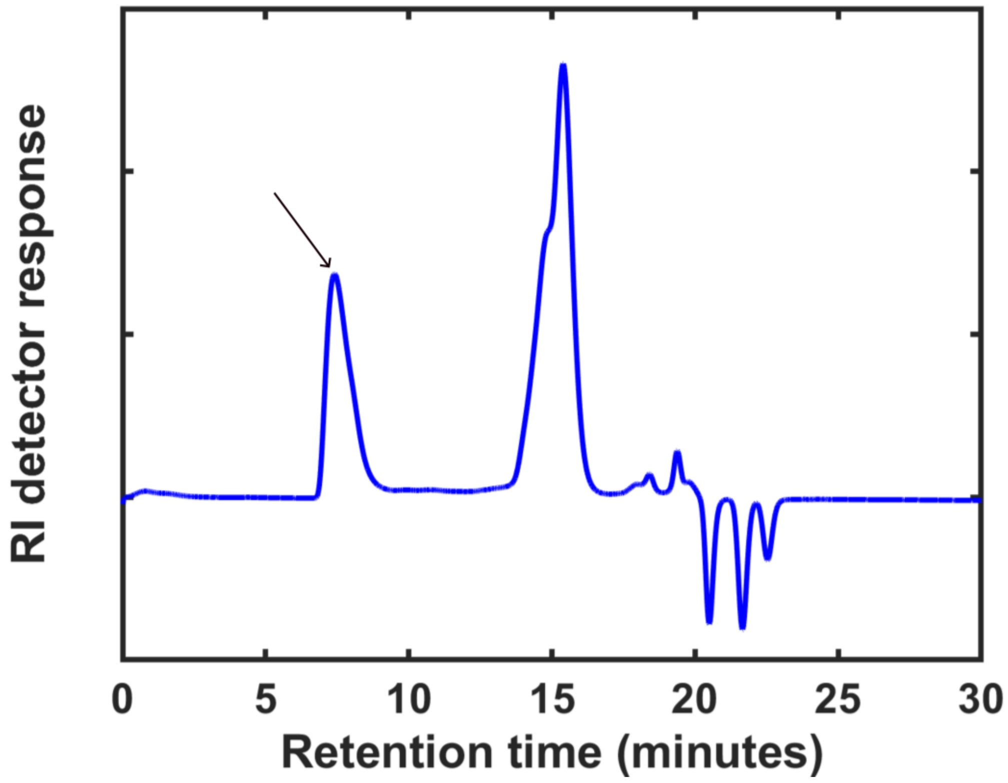

Figure S31. SEC profile for **Candidate 4** MeFSL **(18)**. The peak found at the lower elution time (~8 minutes) possibly originates due to the formation of aggregate or micelles of triblock polymer in the SEC solvent THF (shown by arrow).[9] Thus, the SEC estimated molecular weights for MeFSL is unreliable.

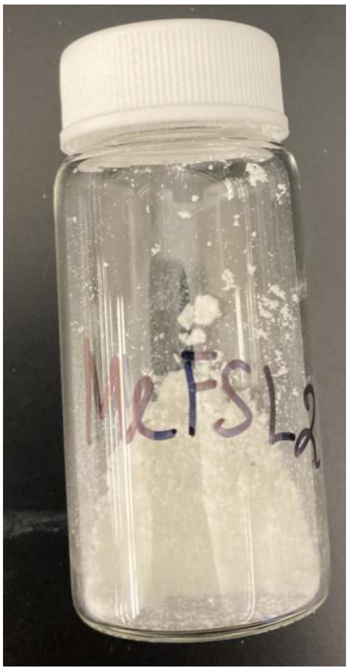

Figure S32. A photograph of the sorbent **Candidate 4** MeFSL2.

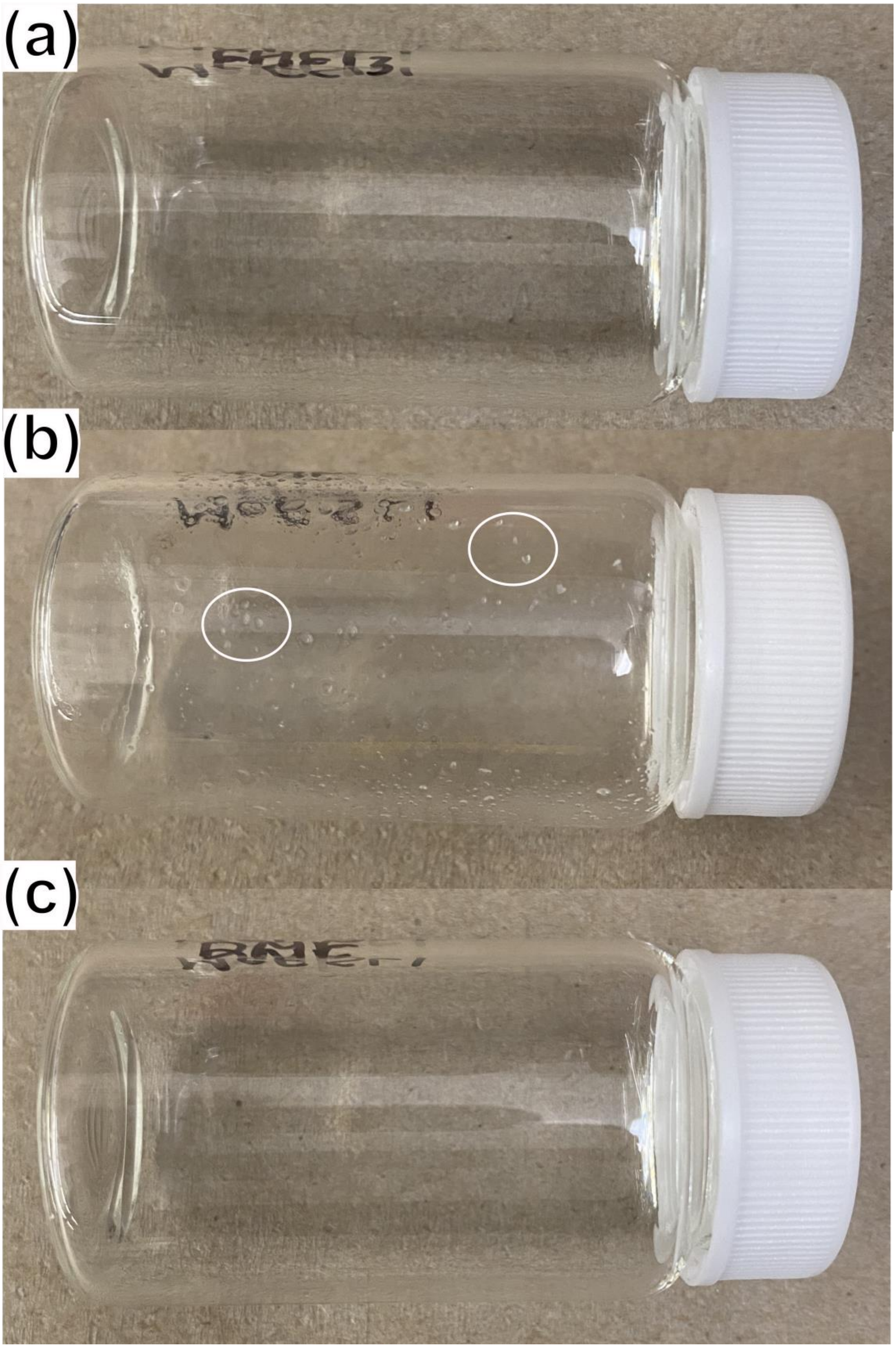

Figure S33. A photograph of 1 wt/vol% solution of **Candidate 4** MeFSL2 in chloroform (a), THF (b) and DMF (c). Photographs were taken after 2 minutes vortex of polymer solutions at room temperature. All solutions demonstrate clear appearances except in THF (b).

Section 3. Thermal Analysis

Weight loss for the below 4 polymer sorbent candidates with a similar range of molecular weights against the temperature was measured by thermogravimetry (TGA), shown in **Figure S34**. The **Candidate 4** MeFSL2 ($M_{n,NMR}$ = 29k gmol^{-1}) (pink) showed a single step decomposition at 340 ºC, whereas the other 3 candidates showed two-stage degradation. For example, **Candidate 1** HSL7 ($M_{n,NMR}$ = 32k gmol^{-1}) (**black**) and **Candidate 2** HFSL5 ($M_{n,NMR}$ = 29k gmol^{-1}) (blue) showed the first weight loss at 260 ºC and 290 ºC, respectively and the second plateau at about 360 ºC and 425 ºC, respectively. The first stage weight loss could be associated with the bond cleavage in ester groups in polylactide and hexyl acrylate, and the second loss might be associated with the degradation of the main chain. It is important to note that **Candidate 2** HFSL5 showed a higher thermal stability compared to **Candidate 1** HSL7, and is attributed to the fluorine substitution in styrene moiety.[5] Lastly we observed two stage decomposition at 180 ºC and 335 ºC for **Candidate 3** HFBuMaSL5 ($M_{n,NMR}$ = 25k gmol^{-1}) (red). Here, the first weight loss could be attributed to the degradation of fluorinated long alkyl chain and the second loss is probably due to the degradation of the main chain.[8,12,13]

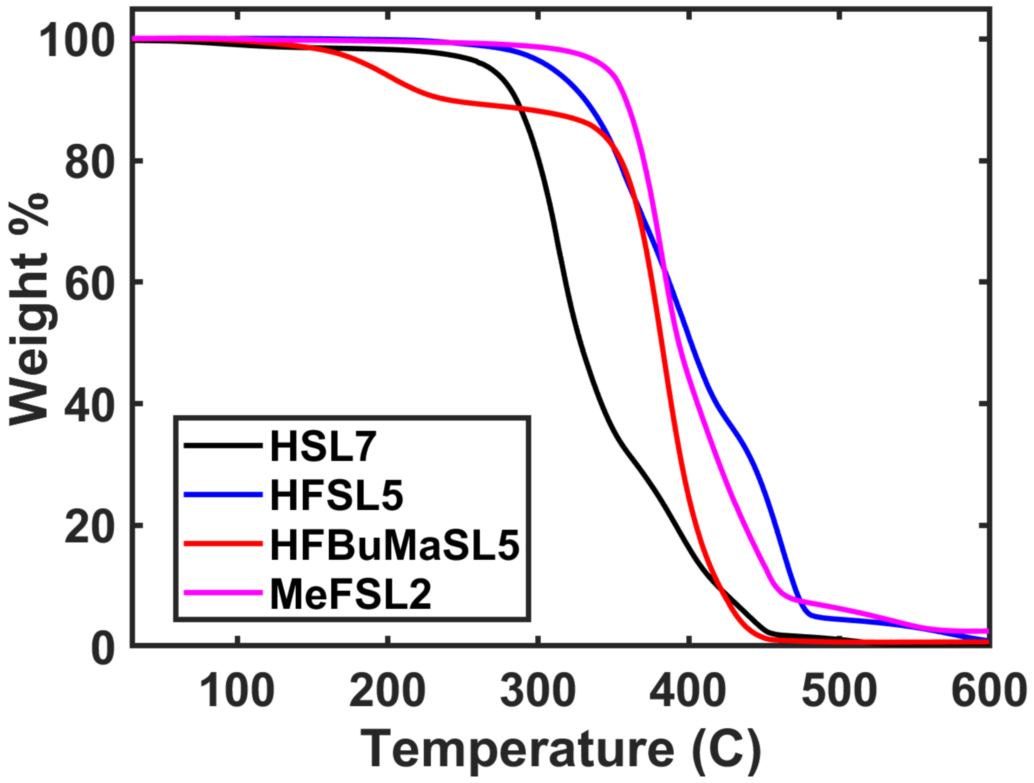

Figure S34. Thermogravimetry (TGA) curves for four polymer sorbent candidates. For TGA analysis, the polymer sample was heated in nitrogen gas environment from 30 to 600 ºC at a heating rate of 10 ºC/min.

Differential scanning calorimetry (DSC) was performed for all polymer sorbent candidates to determine their glass transition temperatures (T_g), as shown in **Figure S35**. As reported in literature,[5,14] the T_g of polystyrene (PS) homopolymer varies from 80-110ºC depending on its molecular weights whereas for polypentafluorostyrene (PFS), its T_g ranges from 77 to 105 ºC. Likewise, T_gs' for polyhexylacrylate (PHA), polylactide (PLA), poly(hexafluorobutyl methacrylate) (HFBuMa) and poly(methyl fluoroacrylate) (MeF) homopolymers are reported to be -57 ºC, 40-60 ºC, 46 ºC and 130.8 ºC, respectively.[15,16,17] The T_g s' for PFS (104.5 °C), PHA (-52 to -56 °C) and PS (96.5–108 °C) in the synthesized polymers were found to be close to the literature reported values. However, the T_g for HFBuMa in **Candidate 3** was hard to extract, as a broad peak was found in the range of 33 to 50 °C and both PLA and HFBuMa peaks overlapped in this temperature range. Similarly, determination of the T_g for MeF in **Candidate 4** was challenging, as a broad peak was found in the range of 80-136 °C and both PS and MeF peaks overlapped in this temperature range. The T_g s' for PLA in the synthesized sorbent candidates were found to be lower (25-34°C) compared to the literature repeated value. Lower T_g is attributed to the shorter PLA chain in our polymer candidates. All the T_g values are listed in **Table 1** and **Figure S35**.

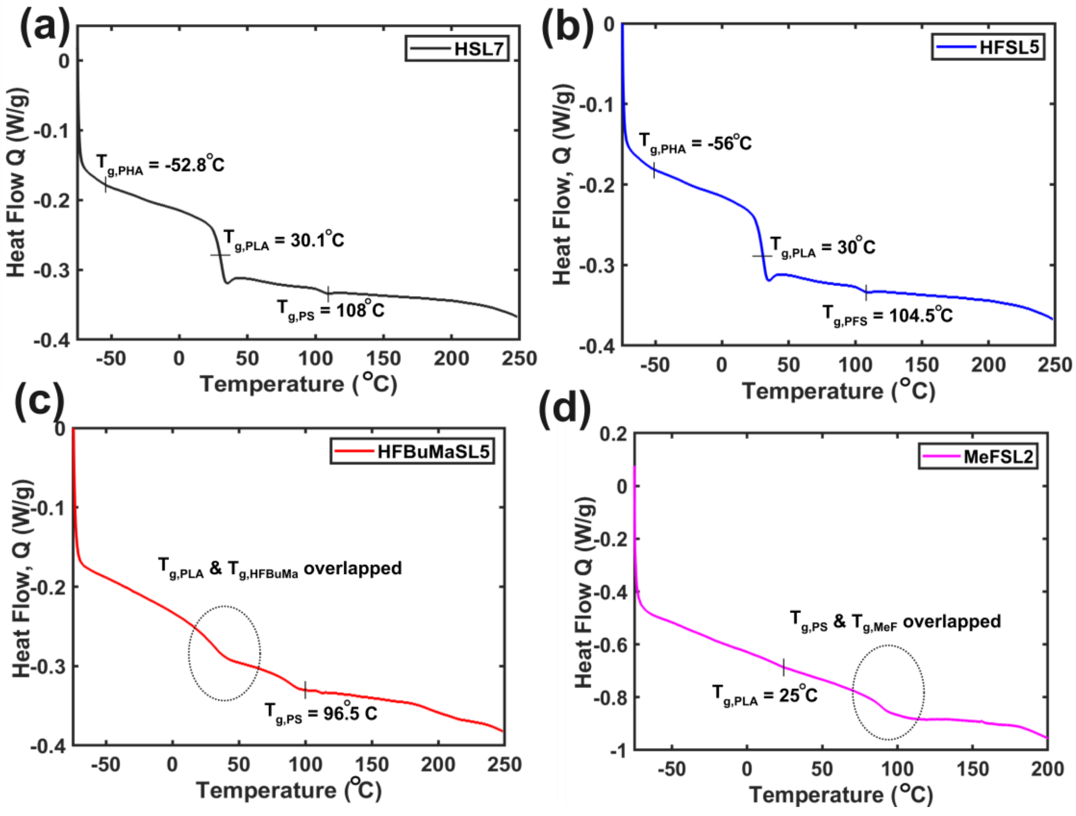

Figure S35. DSC for four polymer sorbent candidates including **Candidate 1** HSL7 (a), **Candidate 2** HFSL5 (b), **Candidate 3** HFBuMaSL5 (c) and **Candidate 4** MeFSL2 (d).

Section 4. Bulk Polymer Self-assembly

Bulk morphologies for each polymer candidate with comparable molecular weights were investigated by transmission SAXS, as shown in **Figure S37**. Polymer films were prepared as follows: a 10 wt% polymer solution was prepared in DMF by stirring the solution at 40 ºC for an hour. The polymer solution was poured into a small Teflon petridish and placed on a hot plate set at 50 ºC for 16-24 hours till the solvent dried off. A glass dome was placed to cover the petridish to ensure a solvent saturated environment throughout the drying process. Dried film was thermally annealed in vacuum oven at 120 ºC for 10 minutes and quenched at room temperature. Free standing films (**Figure S36**) were analyzed by SAXS. Non-fluorinated polymer **Candidate 1** HSL showed poorly ordered SAXS pattern (**a**) which improves to the ordered nanostructure in **Candidate 2** HFSL5 where styrene segment is replaced with pentafluorostyrene (**b**). Higher order reflections with q/q* values of 1, √3, √4 were seen in HFSL5 which suggests hexagonal or cylindrical morphology. Improved ordering for all three fluorinated polymers compared to its non-fluorinated counterpart **Candidate 1** HSL7 suggest an increasing degree of segregation between the chemically different blocks.

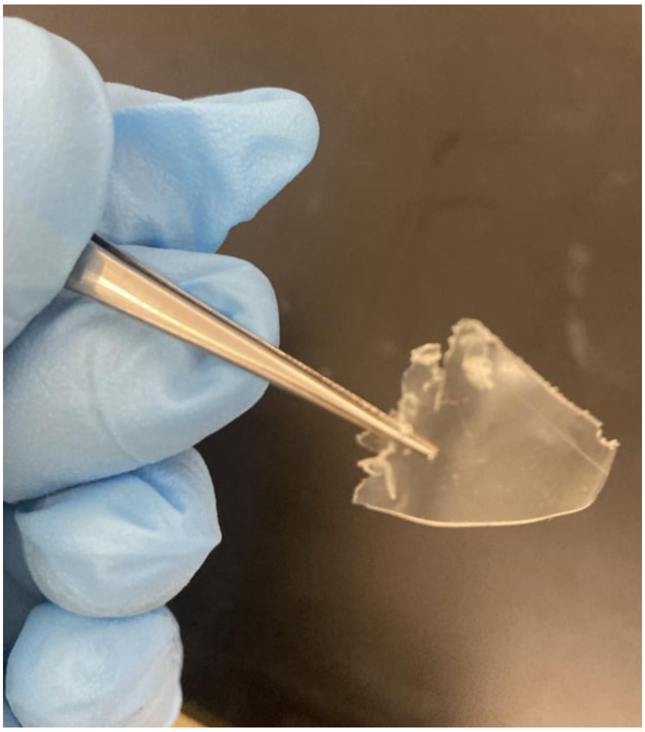

Figure S36. A free-standing HFSL5 film, analyzed by transmission SAXS.

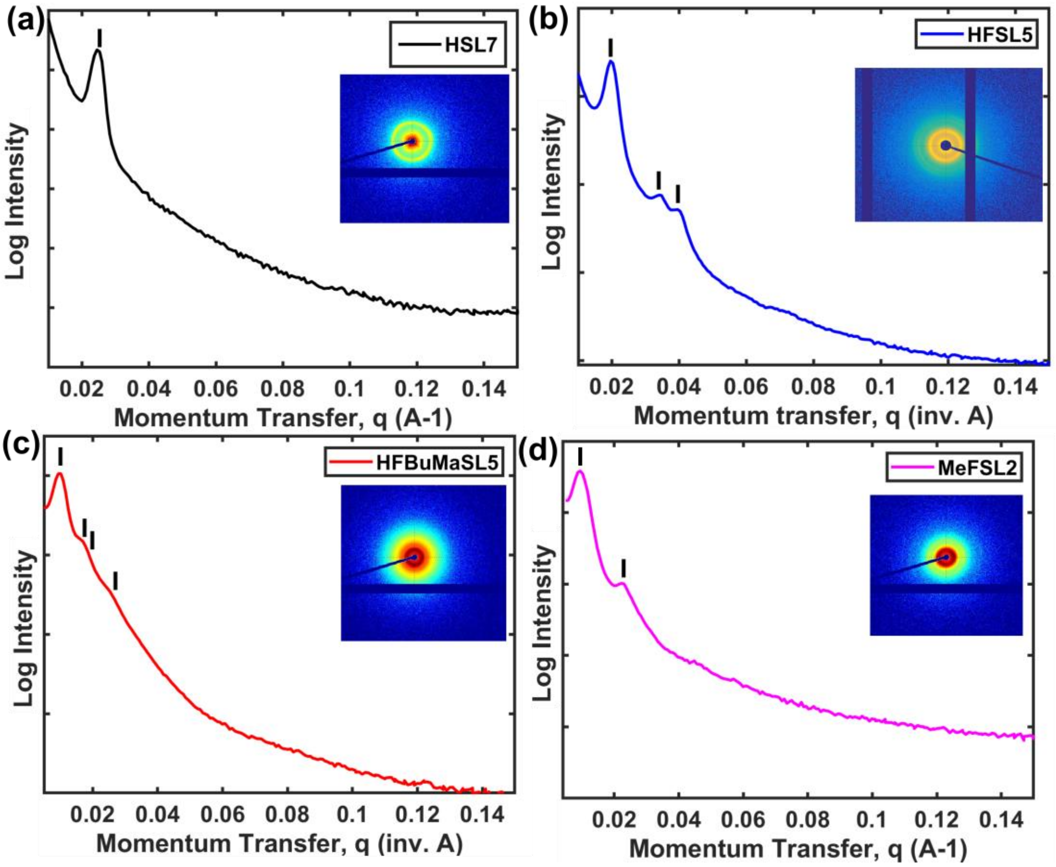

Figure S37. SAXS for bulk polymer films including **Candidate 1** HSL7 (a), **Candidate 2** HFSL5 (b), **Candidate 3** HFBuMaSL5 (c) and **Candidate 4** MeFSL2 (d). No higher order was found for HSL7, whereas a higher reflection of q/q* spacings of 1:√3:√4 were noticed for **Candidate 2** HFSL5. For **Candidate 3** a peak ratio of 1:√3:√4:√7 were found, whereas for MeFSL2 q/q* ratio of 1:√4 were seen.

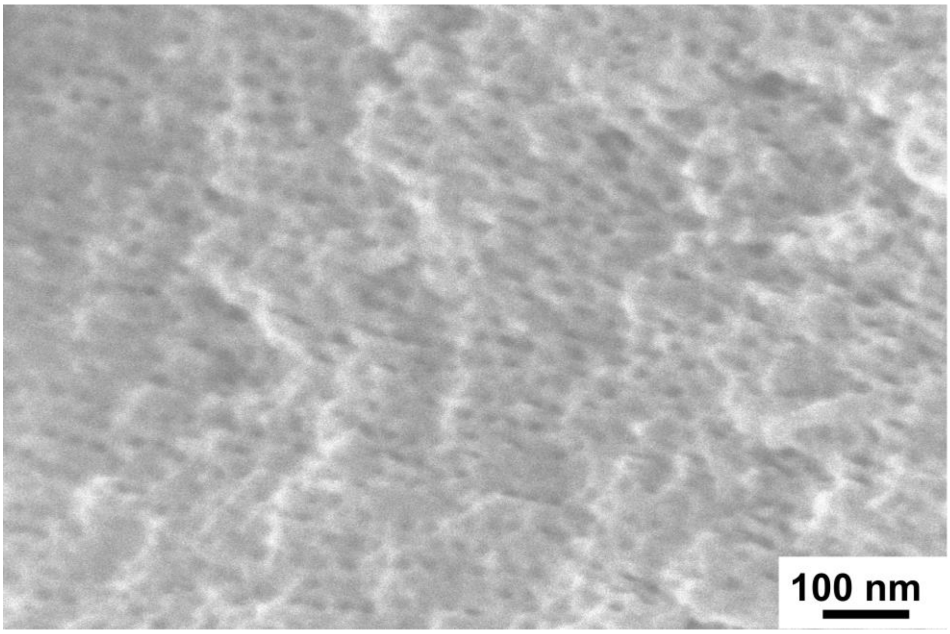

Figure S38. SEM image of porous HFSL5 film after degrading polylactide (PLA) block from it via selective etching by trifluoroacetic acid for 24 hours.[1] The pore-to-pore distance of 31.7±3.6 nm was found after 100 pore size calculations.

Section 5. Polymer Thin Film Phase and Topography Image

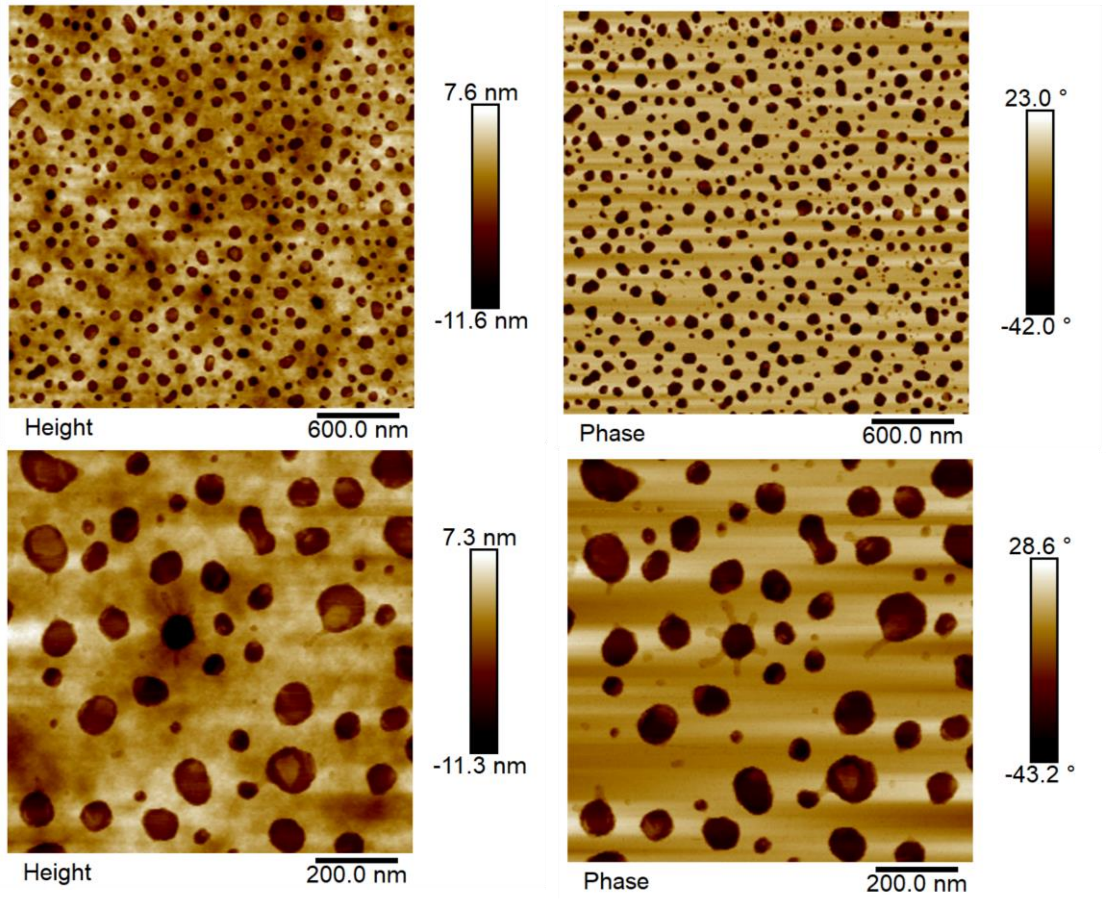

Figure S39. Tapping-mode AFM height and phase images (scan size = 3 x 3 μm, top and 2x2 μm, bottom) of HSL7 thin film (film thickness ~100 nm).

Section 6. Solution Self-assembly

For imaging polymer solution self-assembled structures, 10 mg polymers were first dissolved in 10 mL DMF, and vortexed for 5-10 minutes to get a clear solution (final polymer concentration is 1 mg/mL). Next 2.5 mL deionized water was added dropwise into the polymer solution under stirring condition to induce the formation of aggregates. The solution was shaken for an hour. Next, the solution was dialyzed against DI water for 3 days to remove DMF. Water was changed in every 12-16 hours interval. The resultant solution (one drop) was placed on a carbon coated copper grid (Electron Microscopy Sciences, USA), and then placed on a piece of filter paper to remove excess solvent, and air dried before imaging. Samples were not stained before imaging.

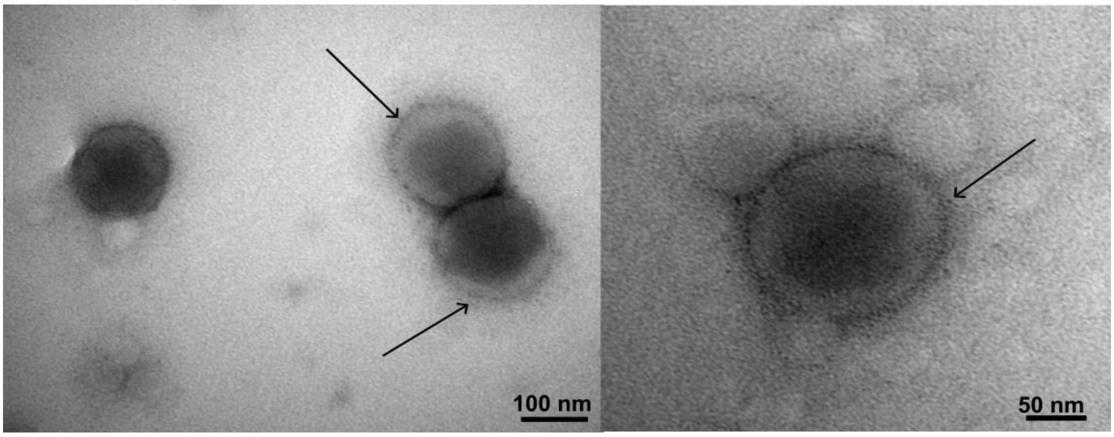

Figure S40. TEM of 25 wt% aqueous solution of HFBuMaSL5, arrows showed the presence of corona.

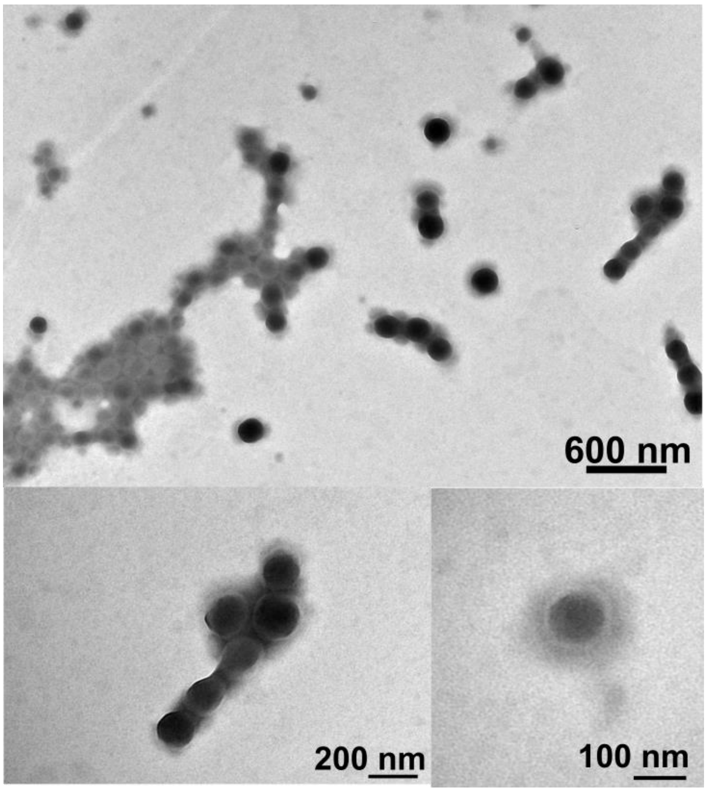

Figure S41. TEM image of 25 wt% aqueous solution of HSL7 polymer with an average diameter of 131±33.2 nm.

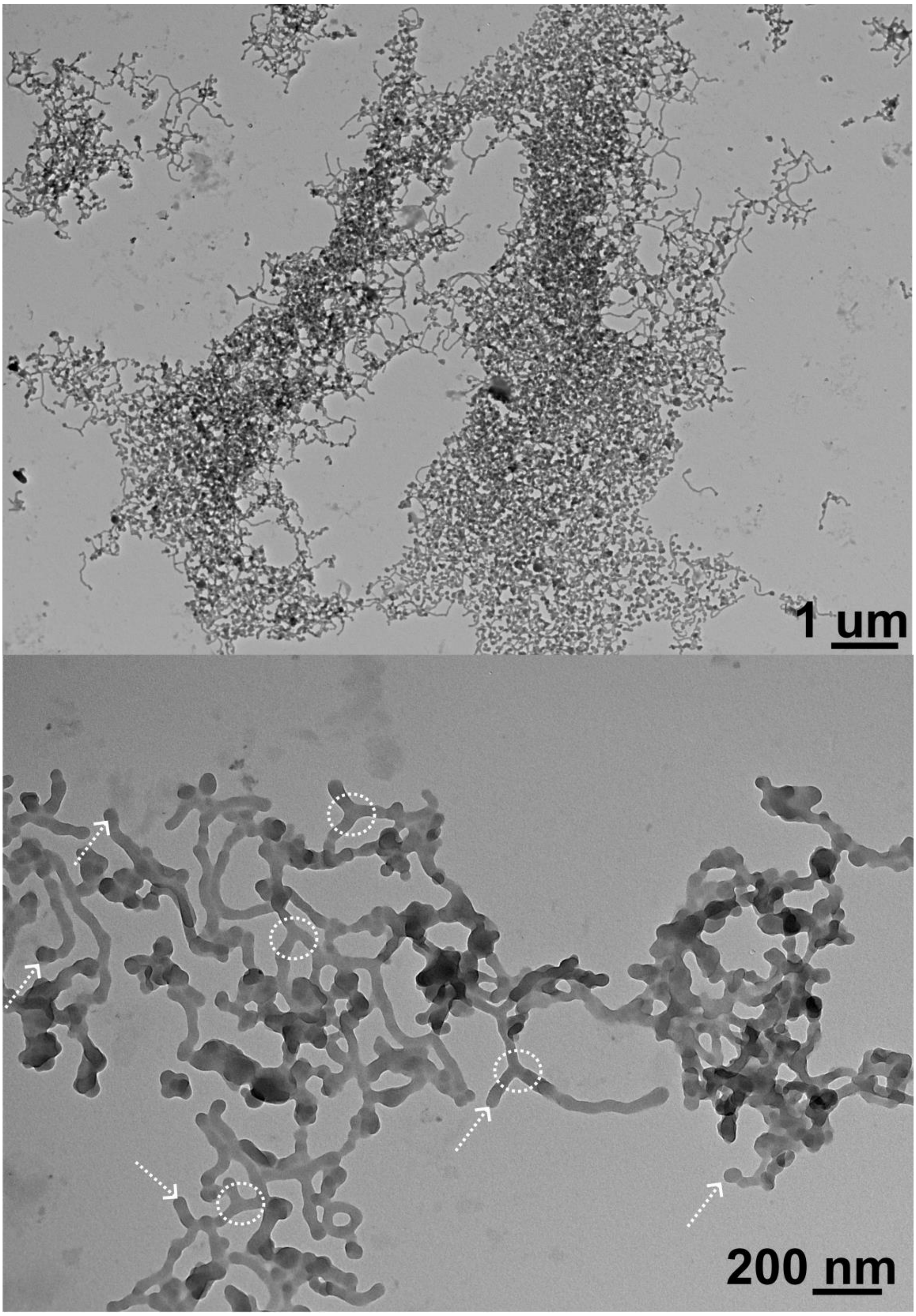

Figure S42. TEM image of 25 wt% aqueous solution of MeFSL2 polymer. Branching points and terminal caps are shown by circles and arrows, respectively.

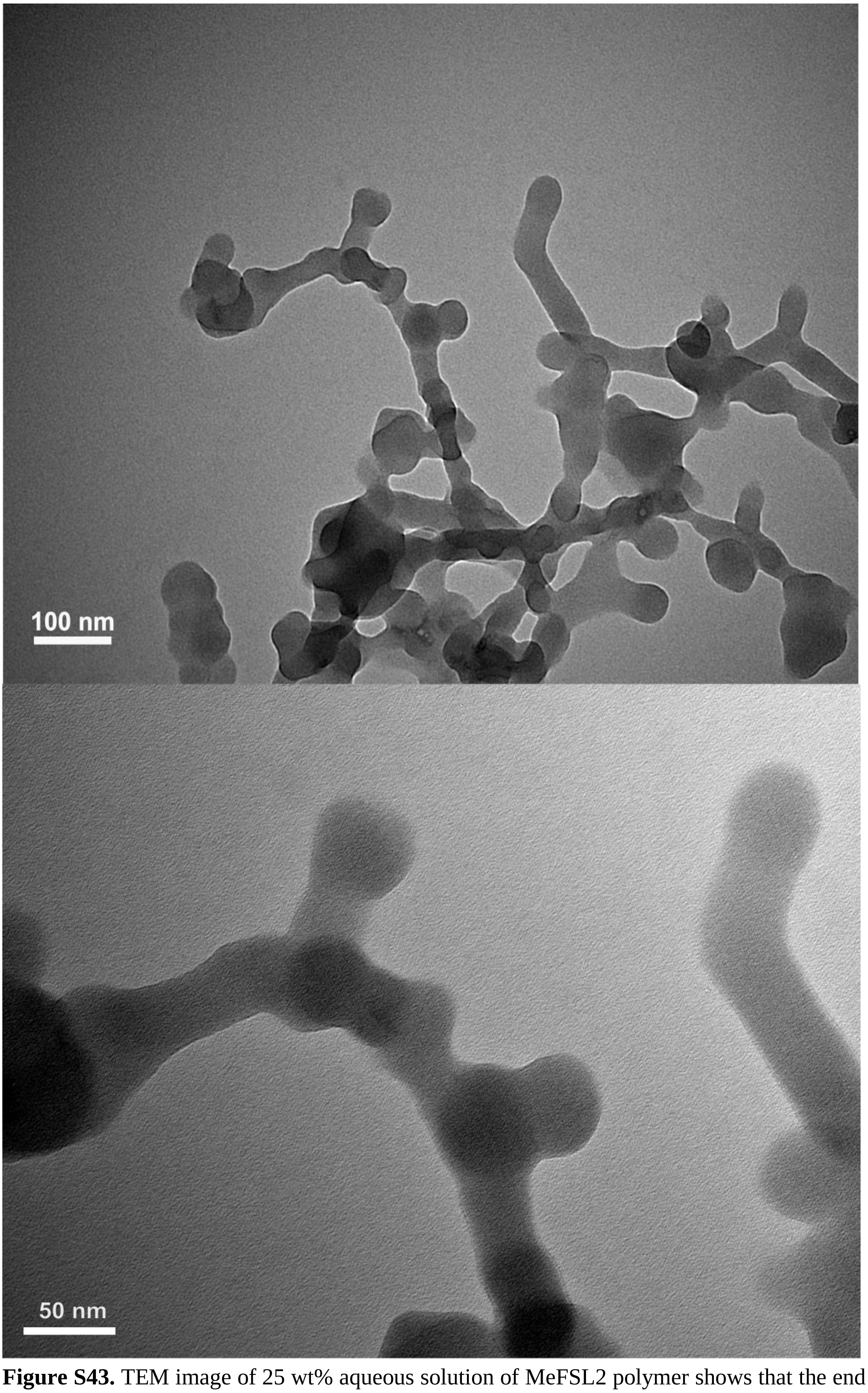

Figure S43. TEM image of 25 wt% aqueous solution of MeFSL2 polymer shows that the end of the cylinders was formed by aggregating several micelles with fluorinated segment as core.

Polymer self-assembled aggregate structures in pure water were captured by TEM. 10 mg each polymer candidate was dispersed in 10 mL water and shaken for 36 hours at room temperature. Resultant mixture was filtered by 0.22 μm filter media and imaged by TEM.

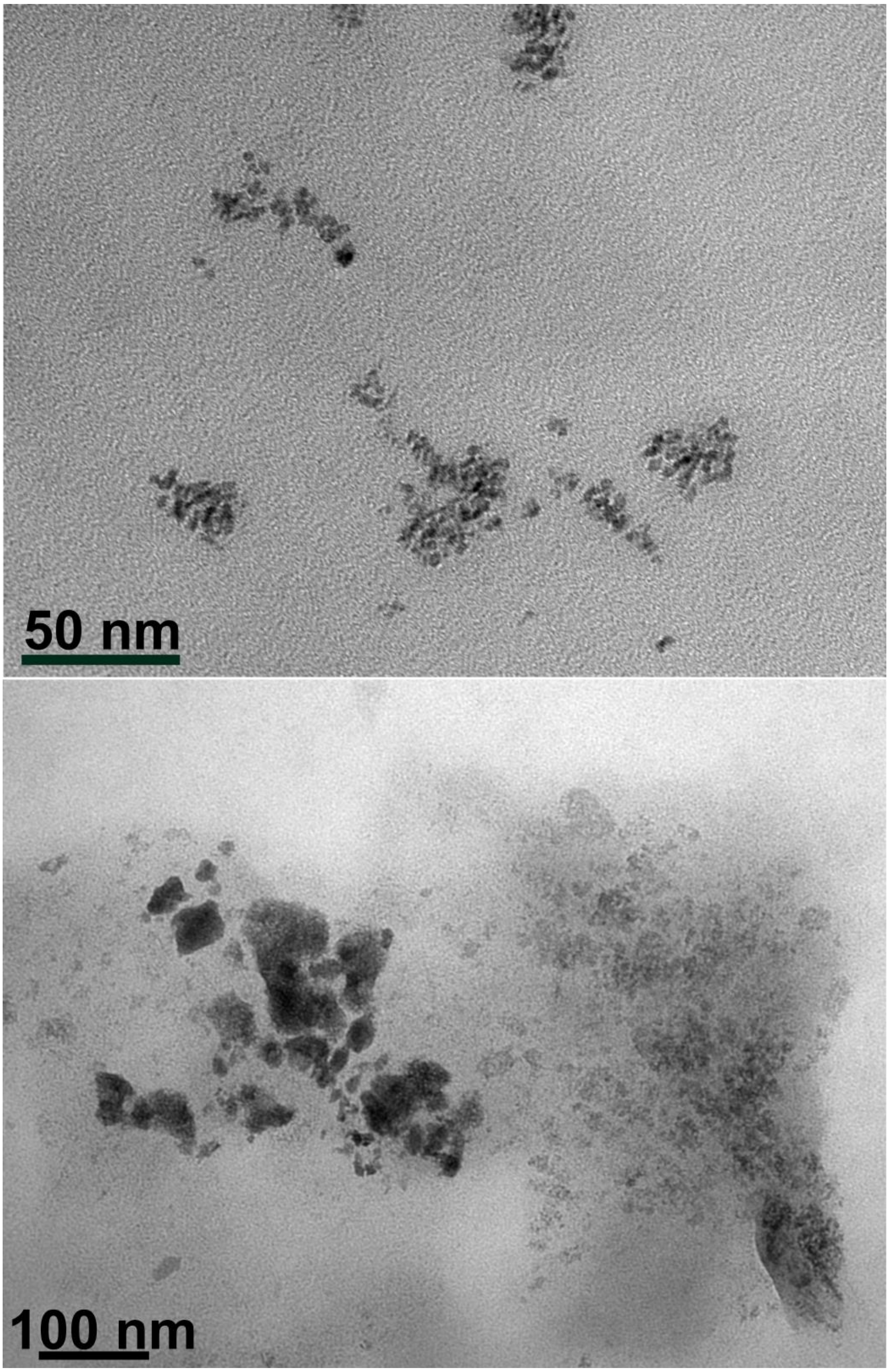

Figure S44. TEM image of MeFSL2 self-assembled aggregates in water (unstained, filtered).

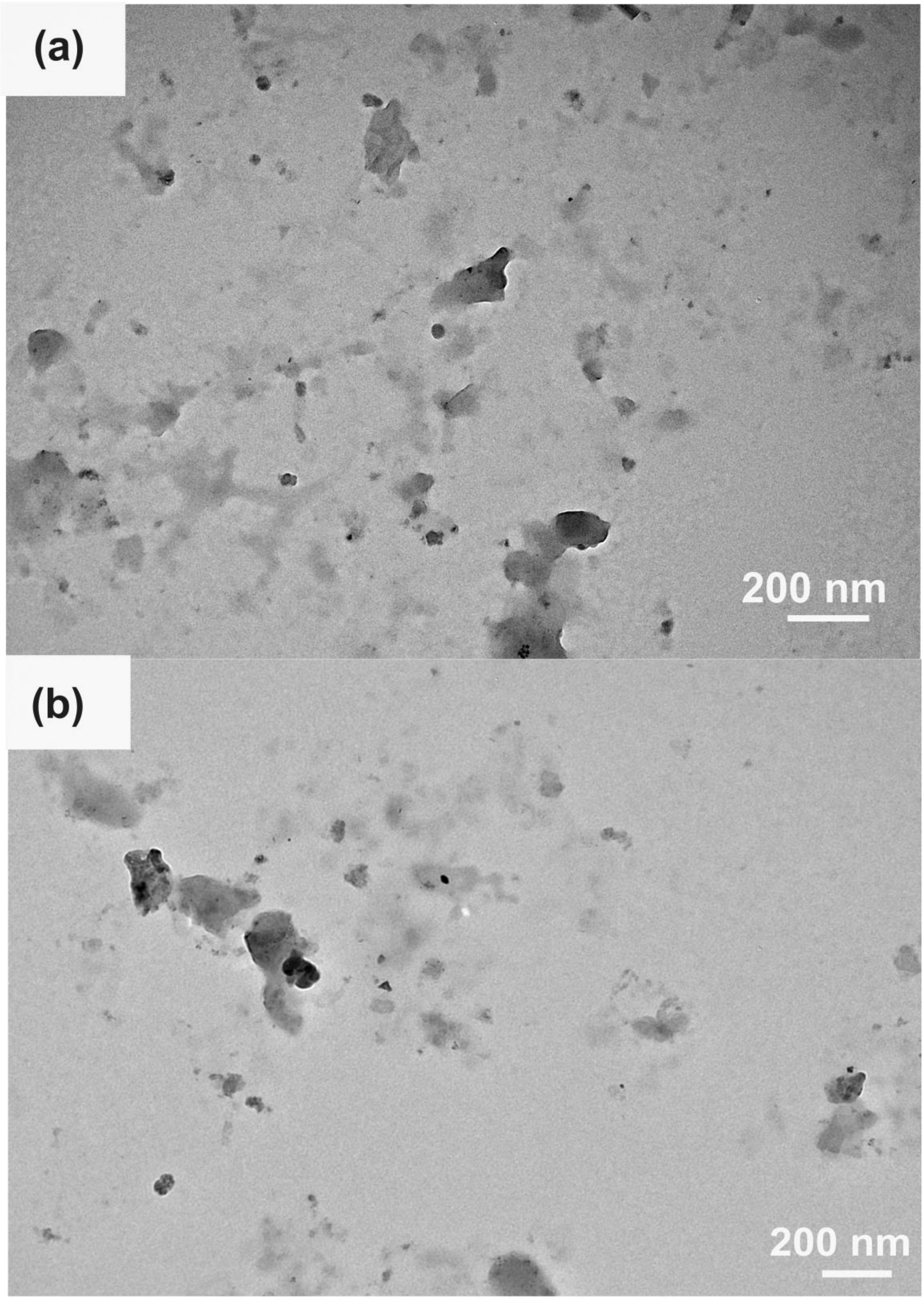

Figure S45. TEM images of **Candidate 2 HFSL5** (a) and **Candidate 3 (HFBuMaSL5)** (b) self-assembled aggregates in water (unstained, filtered).

Section 7. Percentage Removal of PFOA by Proposed Sorbent Candidates

PFOA % Removal

The percentage PFOA removal was determined by equation S1:

$$\text{Removal \%} = [(C_i - C_f) * 100]/C_i \text{ ....(S1)}$$

Here C_i is the initial PFOA concentration in mg L^{-1} (prepared in milliQ water) and C_f is the final concentration of PFOA in mgL^{-1} after each sorbent treatment. All PFOA sorption studies were quantified using PFOA standard solutions for calibration curves. Various concentrations ranging from 0.1 $ngmL^{-1}$ (0.0001 mgL^{-1}) to 25 $ngmL^{-1}$ (0.025 mgL^{-1}) were prepared in methanol and injected in triplicates (to test the reproducibility of the method), such that the peak area was plotted as a function of standard concentrations to generate a quadratic regression. To prepare the sample solution for adsorption study, 1 mgL^{-1} of PFOA aqueous solution with a final volume of 25 mL were individually prepared by dispersing 25 mg of each polymer sorbent candidate into the water samples. The samples were placed on a rocking shaker set at incubator with the constant temperature of 25 °C for 36 hours. After 36 hours polymer-free water samples were decanted to polypropylene centrifuge tubes. All collected samples were diluted to an appropriate concentration in methanol in order to fall within the concentration range of the corresponding calibration curve. An internal standard was added to each dilution at an equal concentration to that in reference standards. The samples were passed through a 0.22 µm glass fiber filters into polypropylene autosampler vials for direct analysis. Each sample was analyzed using high-performance liquid chromatography tandem mass spectrometry (HPLC-MS/MS) in negative ionization mode. Sample dilution procedure is shown below.

Samples	Sample (µL)	Internal standard (µL)	Methanol (µL)	Dilution Factor
1 mgL^{-1} spiked samples	10	25	965	100

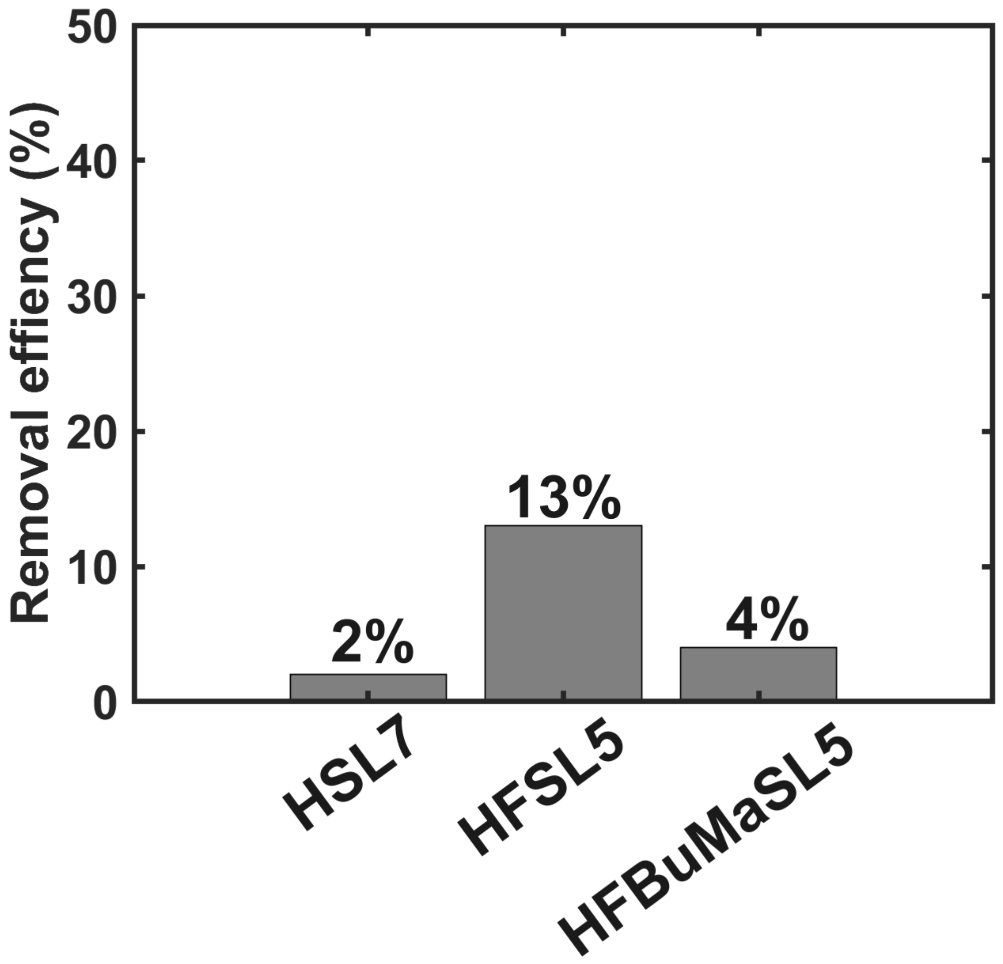

Figure S46. Removal efficiency of 10 mgL^{-1} PFOA by **Candidate 1** HSL7, **Candidate 2** HFSL5 and **Candidate 3** HFBuMaSL5 at ambient temperature. The polymer sorbent concentration was maintained at 1 mg/mL. Sorption tests were conducted for 36 hours.

Section 8.
References

1. Sarkar, A.; Stefik, M. Robust Porous Polymers Enabled by Fast Trifluroacetic Acid Etch with Improved Selectivity for Polylactide. *Mater. Chem. Front.* **2017**, *1*, 1526-1533.
2. Sarkar, A.; Stefik, M. How to Make Persistent Micelle Templates in 24 Hours and Know It Using X-Ray Scattering. *J. Mater. Chem. A* **2017**, *5*, 11840-11853.
3. Sarkar, A.; Evans, L.; Stefik, M. Expanded Kinetic Control for Persistent Micelle Templates with Solvent Selection. *Langmuir* **2018**, *34*, 5738-5749.
4. Sarkar, A.; Thyagarajan, A.; Cole, A.; Stefik, M. Widely Tunable Persistent Micelle Templates via Homopolymer Swelling. *Soft Matter* **2019**, *15*, 5193-5203.
5. Jankova, K.; Hvilsted, S. Preparation of Poly(2,3,4,5,6-pentafluorostyrene) and Block Copolymers with Styrene by ATRP. *Macromolecules* **2003**, *36*, 1753-1758.
6. Remzi Becer, C. R.; Babiuch, K.; Pilz, D.; Hornig, S.; Heinze, T.; Gottschaldt, M.; Schubert, U. S. Clicking Pentafluorostyrene Copolymers: Synthesis, Nanoprecipitation, and Glycosylation. *Macromolecules* **2009**, *42*, 2387-2394.

7. Chen, Z.-C.; Zhu, B.-C.; Li, J.-J.; Zhou, Y.-N.; Luo, Z.-H. Dual-Responsive Copolymer Poly(2,2,3,4,4,4-hexafluorobutylmethacrylate)-*block*-Poly[2-(dimethylamino)ethyl methacrylate] Synthesized via Photo ATRP for Surface with Tunable Wettability. *Journal of Polymer Science, Part A: Polymer Chemistry* **2016**, *54*, 3868-3877.
8. Ji, P.-Y.; Fang, J.; Zhang, Y.-Y.; Zhang, P.; Zhao, J.-B. Novel Single Lithium-Ion Conducting Polymer Electrolyte Based on Poly(Hexafluorobutyl Methacrylate-*co*-Lithium Allyl Sulfonate) for Lithium-Ion Batteries. *ChemElectroChem* **2017**, *4*, 2352-2358.
9. Villarroya, S.; Zhou, J.; Duxbury, C. J.; Heise, A.; Howdle. S. M. Synthesis of Semifluorinated Block Copolymers Containing Poly(Ɛ-Caprolactone) by the Combination of ATRP and Enzymatic ROP in scCO_2. *Macromolecules* **2006**, *39*, 633-640.
10. Otazaghine, B.; Boutevin, B.; Lacroix-Desmazes, P. Controlled Radical Polymerization of n-Butyl α-Fluoroalkylate. 1. Use of Atom Transfer Radical Polymerization as the Polymerization Method. *Macromolecules* **2002**, *35*, 7634-7641.
11. Banerjee, S.; Tawade, B. V.; Ladmiral, V.; Dupuy, L. X.; MacDonald, M. P.; Ameduri, B. Poly(Fluoroacrylate)s with Tunable Surface Hydrophobicity via Radical Copolymerization of 2,2,2-trifluoroethyl-α-Fluoroacrylate and 2-(trifluoromethyl)acrylic Acid. *Polym. Chem.* **2017**, *8*, 1978-1988.
12. Jiang, B.; Zhang, L.; Shi, J.; Zhou, S.; Lioa, B.; Liu, H.; Zhen, J.; Pang, H. Synthesis, Characterization and Bulk Properties of Well-Defined Poly(Hexafluorobutyl Methacrylate)-block-Poly(Glycidyl Methacrylate) via Consecutive ATRP. *Journal of Fluorine Chemistry* **2013**, *153*, 74-81.
13. He, G.; Zhang, G.; Hu, J.; Sun, J.; Hu, S.; Li, Y.; Liu, F.; Xiao, D.; Zou, H.; Liu, G. Low Fluorinated Homopolymer from Heterogeneous ATRP of 2,2,2-Trifluoroethyl Methacrylate Mediated by Copper Complex with Nitrogen-Based Ligand. *Journal of Fluorine Chemistry* **2011**, *132*, 562-572.
14. Atanasov, V.; Kerres, J. Highly Phosphonated Polypentafluorostyrene. *Macromolecules* **2011**, *44*, 6416-6423.
15. Park, P. I. P.; Jonnalagadda, S. Predictors of Glass Transition in the Biodegradable Polylactide and Poly-lactide-co-glycolide Polymers. *Journal of Applied Polymer Science: Part A: Polymer Chemistry* **2006**, *100*, 1983-1987.
16. Miccio, L. A.; Borreon, C.; Casado, U.; Phan, A. D.; Schwartz, G. A. Approaching Polymer Dynamics Combining Artificial Neural Networks and Elastically Collective Nonlinear Langevin Equation. *Polymers* **2022**, *14*, 1573.
17. Hussain, H.; Tan, B. H.; Gudipati, C. S.; Xaio, Y.; Liu, Y.; Davis, T. P.; He, C. B. Synthesis and Characterization of Organic/Inorganic Hybris Star Polymers of 2,2,3,4,4,4-hexafluorobutyl Methacrylate and Octa(aminophenyl)silesquioxane Nano-Cage Made via Atom Transfer Radical Polymerization. *Journal of Applied Polymer Science: Part A: Polymer Chemistry* **2008**, *46*, 7287-7298.
